# First identification of an evolving Middle Stone Age ochre culture at Porc-Epic Cave, Ethiopia

**DOI:** 10.1038/s41598-023-39957-y

**Published:** 2023-09-08

**Authors:** Daniela Eugenia Rosso, Martine Regert, Francesco d’Errico

**Affiliations:** 1 Departament de Prehistòria, Arqueologia i Història Antiga, Grupo de Investigación Prehistoria del Mediterráneo Occidental (PREMEDOC), Universitat de València, Av. Blasco Ibañez 28, 46010 Valencia, Spain; 2 Université Côte d’Azur, CNRS, CEPAM, Nice, France; 3 PACEA UMR 5199, Université de Bordeaux, CNRS, Allée Geoffroy Saint-Hilaire, 33600 Pessac, France; 4 SFF Centre for Early Sapiens Behaviour (SapienCE), University of Bergen, Øysteinsgate 3, Post Box 7805, 5020 Bergen, Norway

**Keywords:** Archaeology, Cultural evolution

## Abstract

The use of mineral pigments, in particular iron-rich rocks, holds significant importance in understanding the emergence and evolution of human cultures. However, sites that have yielded a number of pieces large enough to precisely identify how the use of this material changed through time are rare. In this study, we examine one of the largest known Middle Stone Age (MSA) ochre collections, from Porc-Epic Cave, Ethiopia, consisting of more than 40 kg of ochre (n = 4213 pieces), 21 ochre processing tools and two ochre-stained artefacts. By combining the analysis of the elemental and mineralogical composition of the archaeological material with that of natural ochre collected in the surroundings of the site, and correlating this information with shifts in ochre modification techniques over time, we unveil how MSA inhabitants of Porc-Epic Cave exploited mineral resources. We show that they could predict the properties of different ochre types accessible in their environment, and gradually adapted their technology to cope with changes in raw material availability. Furthermore, the analysis of ochre residues on a painted pebble, likely used to produce red dots on a surface, identifies an ochre type that was specifically employed for symbolic purposes.

## Introduction

In recent years, systematic use of mineral pigments, particularly ochre, has assumed an increasingly important role in the debate on the origin of our species and cultures comparable to ours (Supplementary Information S1)^1,2^. This is mostly due to three factors: first, the earliest use of ochre in Africa coincides with the first appearance of modern anatomical features c. 300 ka^3,4^. Second, ochre gradually increases at sites from East and South Africa to become almost ubiquitous after 160 ka. Third, the deliberate use of ochre to modify the appearance of shell beads suggests that, already at this time and probably earlier, ochre must have been involved in symbolic practices. Ochre, however, remains an ambiguous component of the archaeological record and attempts to understand how it was modified and used, and how practices linked to ochre use evolved over time, are challenging. To address these questions, some studies have focused on the provenance of colouring materials^5–11^ or on their chemical characterisation^12–16^. Others have focused on the techniques used to modify ochre^17–21^. Still others have tried to combine these different approaches at multistratified sites in order to obtain an integrated vision of how the use of this material changed over time^22,23^. However, very few sites have been studied from this perspective since ochre, until recently, was not always systematically documented, and the number of ochre pieces found at most sites is inadequate to establish with certainty whether observed evolutionary trends are real or correspond to random changes in insufficiently documented behavioural variability.

Here we present the integrated study of ochre pieces recovered during the 1975–1976 excavations in the MSA levels at Porc-Epic Cave, Dire Dawa, Ethiopia^24–28^. This site has yielded what is probably the largest collection of African MSA ochre, including around 40 kg of ochre (n = 4213 pieces), 21 ochre processing tools and two ochre-stained artefacts recovered in a more than 2 m deep deposit (Supplementary Information S2). The multiproxy approach applied to the study of this material enables, for the first time, to accurately document how ochre technology gradually evolved over a period of at least 4500 years in a MSA society.

In previous studies on ochre use at Porc-Epic Cave^20,28,29^, ochre types recovered at the site were only distinguished based on macroscopic features, which prevents exploring connections between technology and ochre composition. Furthermore, these first analyses did not provide data regarding ochre availability in the surrounding landscape and its exploitation by MSA Porc-Epic visitors, which complicated inferring how much ochre was brought to the site from distant sources. The first goal of the present study is to identify the elemental and mineralogical composition of the ochre left by MSA people at Porc-Epic Cave and ochre naturally available in erosional deposits close to the site, which originated from upstream formations. The second aim is to determine to what extent differences between ochre types, established in previous studies on the basis of macroscopic features, match variations in elemental and mineralogical composition. This is necessary to determine how Porc-Epic inhabitants exploited available natural resources, whether the composition of ochre played a role in technological and functional choices, whether by choosing visually different ochre types, Porc-Epic inhabitants could predict their properties, and whether such preferences in raw material selection and use changed through time. Previous analyses have shown that a round stone bearing no traces of having been used to process ochre is half covered with ochre residues, as if it had been dipped in a liquid ochred medium to paint the object or use it as a stamp to apply pigment to a soft material^29^. These practices suggest a symbolic rather than functional use of ochre. By comparing the composition and microscopic features of residues present on this object with that of ochre processed at the site, we attempt to determine what kind of ochre was preferentially used for symbolic purposes at Porc-Epic.

## Previous research on ochre use at Porc-Epic Cave

The reappraisal of the ochre collection recovered during Williamson’s excavations in the 1970s has been the subject of three complementary contributions^20,28,29^.

The spatial and stratigraphic analysis of ochre pieces and ochre processing tools^28^ showed that ochre pieces are present throughout the sequence (between 30 and 280 cm below datum), with the highest frequency in MSA levels situated between 60 and 160 cm below datum^28^. The comparison of the distribution of ochre pieces and gastropod opercula dated by ^14^C indicates that ochre use at Porc-Epic appears to have begun around or before 45 cal kyr BP, becoming particularly intense at c. 40 cal kyr BP. Concomitant changes in the location of ochre concentration areas and ochre processing tools suggest areas devoted to ochre processing shifted spatially over time. Two ochre concentrations (S2 Supplementary Fig. 3) were identified: one was located in the northeastern area (NEA) of the cave (squares 08N–07W, 08N–08W, 09N–07W, 10N–07W) at 100 to 190 cm below datum, and the other in the southeastern area (SEA, squares 04N-04W, 04N-05W and 04N-07W) between 60 and 100 cm below datum. The NEA concentration accounts for 50.73% (n = 1373) of the ochre pieces recovered in those layers (100 to 190 cm below datum), and yielded twelve ochre processing tools and one ochre-stained artefact. The SEA yielded 62.27% (n = 558) of the ochre pieces recovered in the same layers (60 to 100 cm below datum), as well as two processing tools.

The analysis of upper and lower grindstones^29^ (Supplementary Information S3) has demonstrated that different types of rocks, sometimes exogenous, were used to process ochre. The chemical analysis of ochre residues revealed that a variety of ochre types were processed and that different processing techniques were involved. Different shades and colours of ochre powder were produced, probably for a variety of utilitarian and symbolic activities.

**Figure 1:**
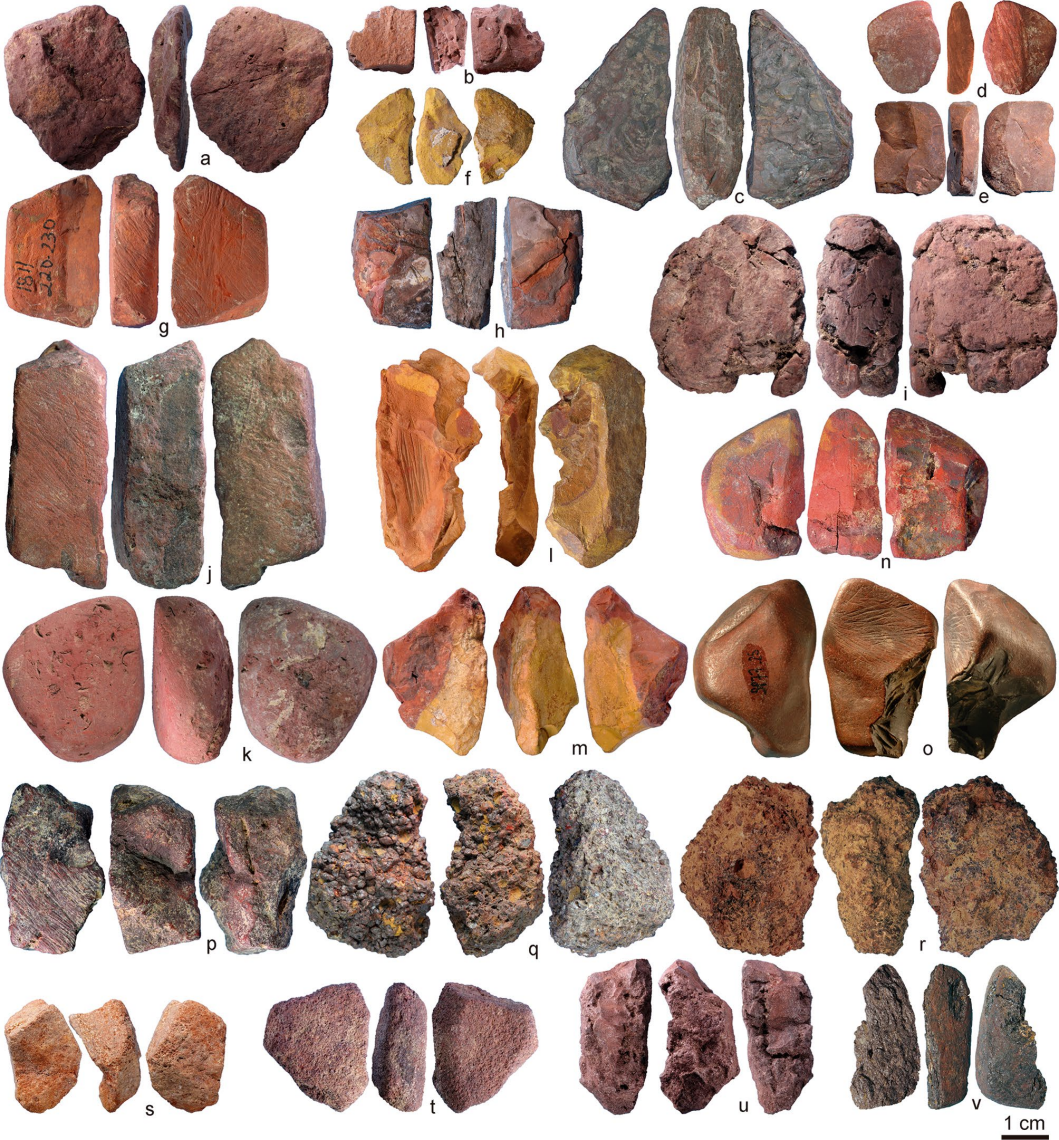
Ochre pieces from Porc-Epic Cave. (**a**) Ochre piece PE17, SFG; (**b**) ochre piece PE295, SFG; (**c**) ochre piece PE538, SFG; (**d**) ochre piece PE913, SFG; (**e**) ochre piece PE919, SFG; (**f**) ochre piece PE930, SFG; (**g**) ochre piece PE987, SFG; (**h**) ochre piece PE994, SFG; (**i**) ochre piece PE1635, SFG; (**j**) ochre piece PE1914, SFG; (**k**) ochre piece PE1927, SFG; (**l**) ochre piece PE306, BFG; (**m**) ochre piece PE521, BFG; (**n**) ochre piece PE102, HFG; (**o**) ochre piece PE1419, HFG; (**p**) ochre piece PE1752, HFG; (**q**) ochre piece PE1510, CG; (**r**) ochre piece PE1628, CG; (**s**) ochre piece PE101, FS; (**t**) ochre piece PE973, FS; (**u**) ochre piece PE436, PFG; (**v**) ochre piece PE1812, PFG. Scale = 1 cm.

**Figure 2:**
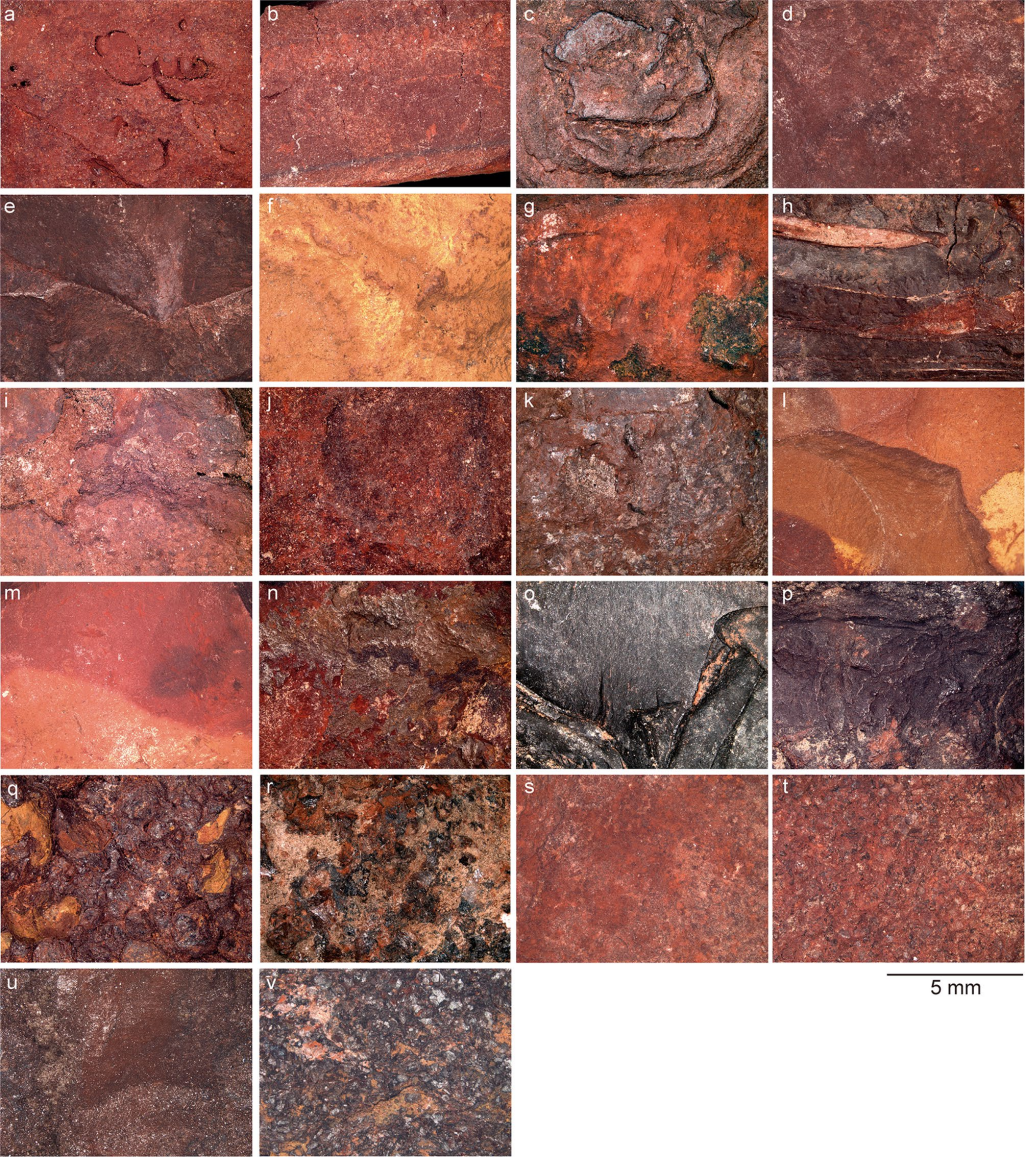
Detailed view of ochre pieces from Porc-Epic Cave. (**a**) Ochre piece PE17, SFG; (**b**) ochre piece PE295, SFG; (**c**) ochre piece PE538, SFG; (**d**) ochre piece PE913, SFG; (**e**) ochre piece PE919, SFG; (**f**) ochre piece PE930, SFG; (**g**) ochre piece PE987, SFG; (**h**) ochre piece PE994, SFG; (**i**) ochre piece PE1635, SFG; (**j**) ochre piece PE1914, SFG; (**k**) ochre piece PE1927, SFG; (**l**) ochre piece PE306, BFG; (**m**) ochre piece PE521, BFG; (**n**) ochre piece PE102, HFG; (**o**) ochre piece PE1419, HFG; (**p**) ochre piece PE1752, HFG; (**q**) ochre piece PE1510, CG; (**r**) ochre piece PE1628, CG; (**s**) ochre piece PE101, FS; (**t**) ochre piece PE973, FS; (**u**) ochre piece PE436, PFG; (**v**) ochre piece PE1812, PFG. Scale = 5 mm.

This is also supported by the analysis of ochre pieces^20^, that showed the presence of a great variety of ochre types. Six raw material types were determined based on colour, texture, inclusions, hardness and density (Supplementary Information S4, S4 Supplementary Table 1). The soft fine-grained type (SFG, Figs. 1a–k, 2a–k) is the most frequent raw material category, followed by coarse-grained type (CG, Figs. 1q,r, 2q,r), banded fine-grained type (BFG, Figs. 1l,m, 2l,m) and hard fine-grained type (HFG, Figs. 1n–p, 2n–p). Ferruginous sandstone (FS, Figs. 1s,t, 2s,t) is systematically present, but in very low proportions. A platy fine-grained type (PFG, Figs. 1u,v, 2u,v) is only recorded sporadically in levels in which ochre is abundant. Proportions of the different raw material types do not change considerably throughout the sequence (S4 Supplementary Fig. 2). This is interpreted as a continuity in the functions fulfilled by different types of ochre through time.

The morphology of the pieces before being modified (slab, pebble, nodule, irregular) was also recorded. This helped determine that the proportion of slabs increases with time, that of irregular pieces decreases, and that of nodules and pebbles does not considerably change throughout the stratigraphy (S4 Supplementary Fig. 3).

The technological analysis of ochre pieces^20^ showed that modified pieces (Supplementary Information S4, S4 Supplementary Fig. 1) are exceptionally abundant at the site, with flaking and grinding representing the most common techniques applied. Modified pieces decrease through time, with flaking, scraping, and pitting becoming increasingly frequent, smoothing remaining stable, and grinding progressively dropping (S4 Supplementary Fig. 4). This gradual shift in preferred processing techniques was identified and interpreted as reflecting gradual cultural drift. It was also observed that the larger the pieces, the more ground facets they bear. This finding suggests curation of large ground objects and is consistent with the idea that large pieces were repeatedly ground to produce small quantities of ochre powder each time.

## Results

### Ochre characterisation

**Figure 3:**
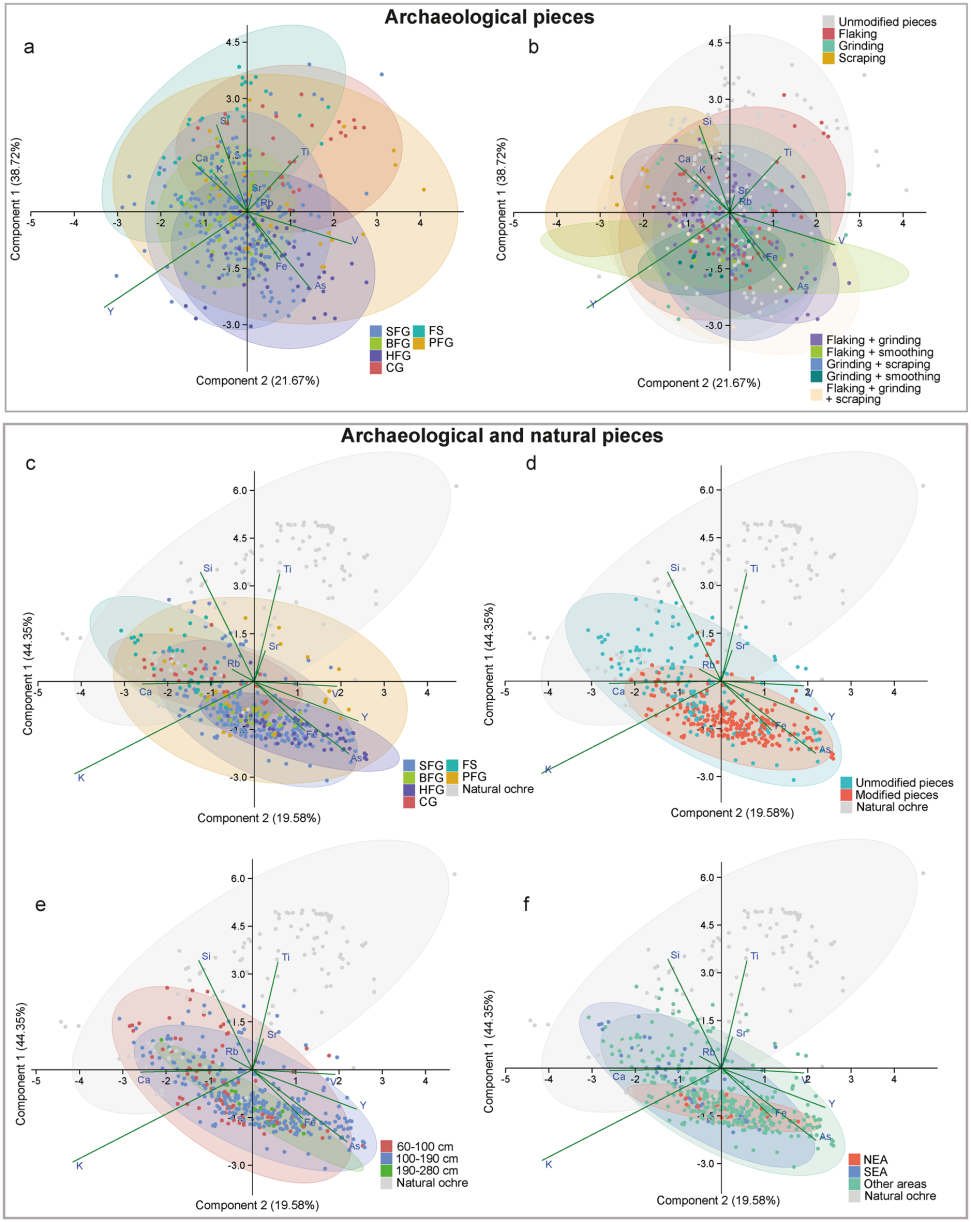
Principal component analysis of the ochre pieces from Porc-Epic Cave and natural ochre from the surrounding area. PCA using a centred logarithm ratio (clr) transformation of the 10 most frequently detected major, minor and trace elements by EDXRF (Si, K, Ca, Ti, Fe, V, As, Rb, Sr, Y) of archaeological pieces (**a,b**) and both archaeological and natural ochre pieces (**c–f**), by raw material type (**a,c**), modification type (**b**), modification occurrence (**d**), level (**e**) and accumulation area (**f**). *SFG* soft fine-grained, *BFG* banded fine-grained, *HFG* hard fine-grained, *CG* coarse-grained, *FS* ferruginous sandstone, *PFG* platy fine-grained, *NEA* northeastern area, *SEA* southeastern area.

Analyses conducted using X-ray fluorescence spectrometry (EDXRF) show that 56% of analysed pieces contain more than 50% Fe, and 75% of the pieces contain more than 36% of this element. Si, K, Ca and Ti are present in all pieces as major or minor elements, with Si being the most represented (Fig. 3, Table 1, S5 Supplementary Table 1). The proportions of the major elements are highly variable, indicating the sample is composed of a wide range of rocks. Mn is present as a minor or trace element. V, Cr, Ga, As, Rb, Sr, Y and Ba are present as trace elements, with As, Sr and Y being the most frequently identified above the limit of detection.

**Table 1:** Proportion of elements from Porc-Epic archaeological ochre pieces and natural ochre collected around the site identified using EDXRF.

	Archaeological ochre pieces*					Natural ochre*				
	Si	K	Ca	Fe	Ti	Si	K	Ca	Fe	Ti
Min	2.01	0.10	0.14	4.15	0.02	3.47	0.02	0.04	3.88	0.03
Max	43.72	3.28	13.44	86.48	10.64	97.60	5.01	40.26	74.30	5.27
Mean	11.12	0.89	1.99	49.33	0.51	51.00	0.70	2.79	20.42	1.68
Std. error	0.42	0.03	0.08	0.95	0.06	2.40	0.13	0.64	1.68	0.12
Variance	69.28	0.29	2.79	356.40	1.46	639.48	1.05	45.13	314.60	1.47
Stand. dev	8.32	0.54	1.67	18.88	1.21	25.29	1.02	6.72	17.74	1.21
Median	8.14	0.77	1.51	54.21	0.23	55.70	0.29	0.67	11.56	1.58

*Values are calculated on results from 80 archaeological ochre pieces and 39 natural ochre pieces.

**Figure 4:**
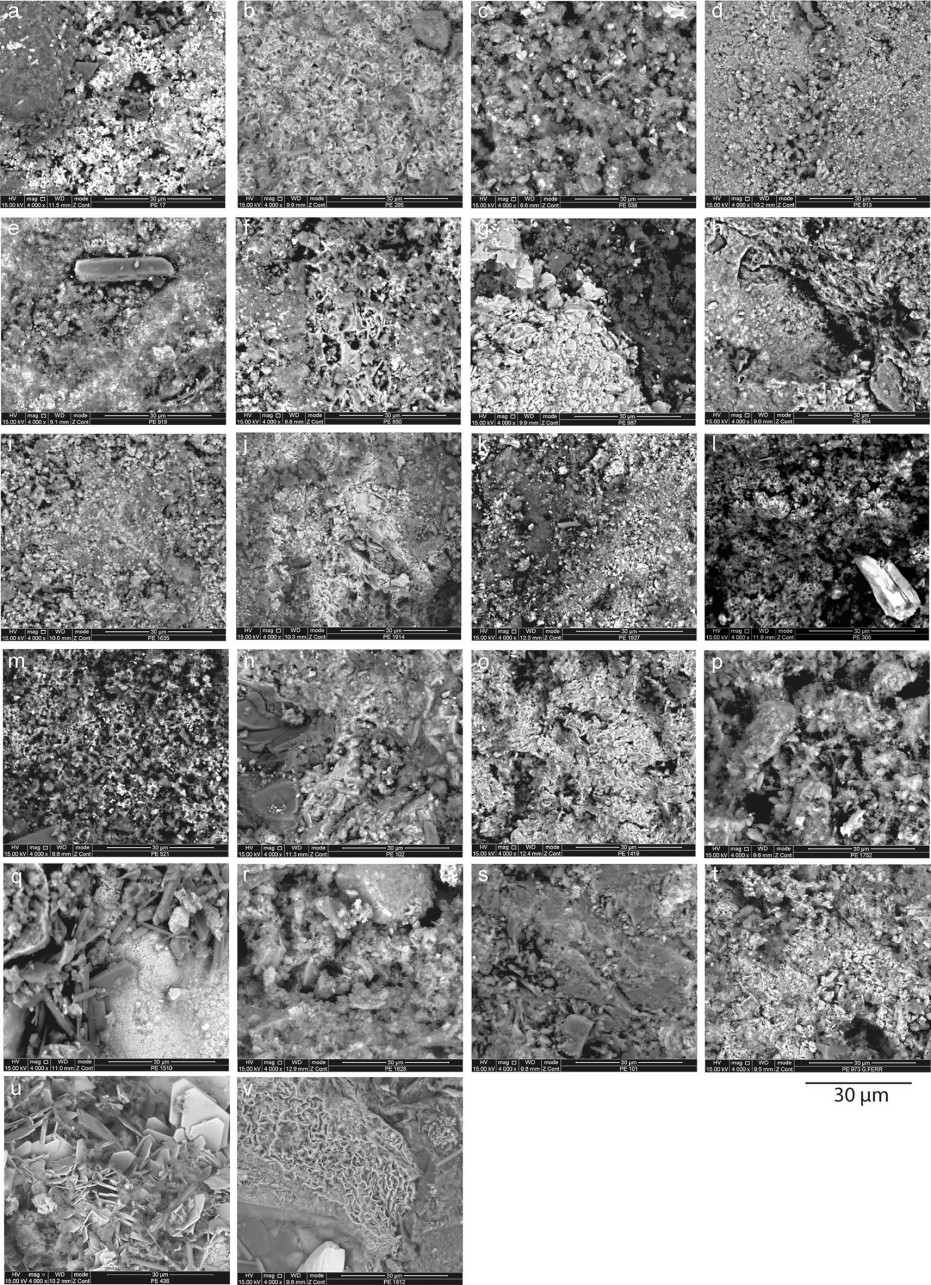
SEM images of ochre pieces from Porc-Epic Cave. All figures are in backscattered electron (BSE) mode. (**a**) Ochre piece PE17, SFG; (**b**) ochre piece PE295, SFG; (**c**) ochre piece PE538, SFG; (**d**) ochre piece PE913, SFG; (**e**) ochre piece PE919, SFG; (**f**) ochre piece PE930, SFG; (**g**) ochre piece PE987, SFG; (**h**) ochre piece PE994, SFG; (**i**) ochre piece PE1635, SFG; (**j**) ochre piece PE1914, SFG; (**k**) ochre piece PE1927, SFG; (**l**) ochre piece PE306, BFG; (**m**) ochre piece PE521, BFG; (**n**) ochre piece PE102, HFG; (**o**) ochre piece PE1419, HFG; (**p**) ochre piece PE1752, HFG; (**q**) ochre piece PE1510, CG; (**r**) ochre piece PE1628, CG; (**s**) ochre piece PE101, FS; (**t**) ochre piece PE973, FS; (**u**) ochre piece PE436, PFG; (**v**) ochre piece PE1812, PFG. Scales = 30 μm.

Analyses conducted using scanning electron microscopy coupled with energy dispersive X-ray spectroscopy (SEM–EDS) identify all major elements detected by EDXRF as well as minor elements (Mn and Ba) (Fig. 4, Table 2, S5 Supplementary Fig. 1, S5 Supplementary Table 2). SEM–EDS also identifies six elements falling out of the range of detection of EDXRF because of their low atomic weight (Na, Mg, Al, P, S, Cl). The presence of these elements confirms the identification of mineral phases detected by X-ray diffraction (XRD), micro X-ray diffraction (µ-XRD) and micro-Raman spectroscopy (µ-RS): clays, feldspars, micas, sulphates and phosphates.

**Table 2:** Summarised results of SEM–EDS analyses conducted on ochre pieces from Porc-Epic Cave.

Num	Raw mat	Clay-sized particles (< 4 µm)		Silt and sand-sized particles (> 4 µm)	
		Shape	Composition*	Shape	Composition*
17	SFG	Irr	Fe (iron oxide)	–	–
		Plat	Si, Al, Ca, K, Mg (clay min)		
		Irr	Si (silicate)		
295	SFG	Aggl/irr	Fe (iron oxide)	Irr	Si (silicate)
				Subcirc	Ca, S (calcium sulphate)
538	SFG	Plat	Fe (iron oxide)	Irr/ang	Fe (iron oxide)
		Aggl	Si, Al, Fe, Na, K, Ca (clay min)	Irr	Si (silicate)
		Subcirc	Ni (undet Ni-rich particle)	Plat	Si, Al, Fe, K, Ca (mica)
913	SFG	Irr	Fe (iron oxide)	Irr/aggl	Fe (iron oxide)
				Plat	Si (silicate)
				Aggl	Si (silicate)
				Irr	Si, Al, Ca, K, Mg (mica)
				Ang	Ca, S (calcium sulphate)
919	SFG	Irr/subcirc	Fe (iron oxide)	Irr	Si (silicate)
		Aggl	Ba, S (barium sulphate)	Ang	Ca, S (calcium sulphate)
930	SFG	Irr	Fe (iron oxide)	Aggl	Fe (iron oxide)
		Acic	Fe (iron oxide)	Irr	Si, Al, K, Ca (K-rich feldspar)
		Irr/aggl	Si, Al, Ca, K, P (clay min)		
987	SFG	Aggl	Fe, Mn (iron oxide)	Ang	Fe, Mn (iron oxide)
				Ang/plat	Si (silicate)
				Tab	Ba, S (barium sulphate)
994	SFG	Plat/aggl	Fe (iron oxide)	Plat	Si, Al, K, Ca (K-rich mica)
		Irreg	Fe (iron oxide)		
		Irreg	Si, Al, K, Ca (clay min)		
		Plat	Si, Al, K, Ca (clay min)		
1635	SFG	Irr/aggl	Fe, Mn (iron oxide)	Irr	Fe, Mn (iron oxide)
		Aggl	Si, Ca, Al, K, P (clay min)	Irr	Si (silicate)
		Aggl	Ca, S (calcium sulphate)		
1914	SFG	Aggl	Fe (iron oxide)	Aggl	Fe (iron oxide)
		Irr	Si (silicate)		
		Plat/aggl	Ba, S (barium sulphate)		
1927	SFG	Aggl	Fe (iron oxide)	Irr/aggl	Fe (iron oxide)
		Aggl	Ca (carbonate)		
306	BFG	Irr/plat	Fe (iron oxide)	Tab	Fe (iron oxide)
		Irr/plat	Si, Al, Ca, K, Mg (clay min)		
521	BFG	Aggl	Fe (iron oxide)	Plat	P, Ca (calcium phosphate)
		Irr	Fe (iron oxide)		
		Plat	Si, Al, Ca (clay min)	Irr	Ca (carbonate)
		Plat	Si (silicate)		
		Subcirc	Si (silicate)		
102	HFG	Irr	Fe (iron oxide)	Irr	Fe (iron oxide)
				Irr	Si (silicate)
				Ang/plat	Si, Al (mica)
1419	HFG	Irr	Fe (iron oxide)	Plat	Fe (iron oxide)
				Ang	Si (silicate)
1752	HFG	Aggl	Fe (iron oxide)	Plat	Fe (iron oxide)
		Aggl	Si, Ca, Al, K, P, Mg (clay min)	Aggl	Si (silicate)
1510	CG	Aggl/subcirc	Fe (iron oxide)	Plat	Fe (iron oxide)
				Subcirc	Si (silicate)
				Plat	Ca, S (calcium sulphate)
1628	CG	Plat	Fe (iron oxide)	Subcirc	Si (silicate)
		Plat	Si, Al, Ca, K, Mg (clay min)	Plat	Si, Al, Ca, Na (Ca-rich feldspar)
101	FS	Aggl	Fe (iron oxide)	Irr/plat	Si (silicate)
		Irr/plat	Fe (iron oxide)	Tab	Si (silicate)
973	FS	Plat	Fe (iron oxide)	Aggl	Fe (iron oxide)
		Aggl	Si, Ca, Fe, Al, K (clay min)	Irr/ang	Si (silicate)
				Ang	Ca, S (calcium sulphate)
436	PFG	Plat	Fe (iron oxide)	Plat	Fe (iron oxide)
		Aggl	Si, Ca, Al (clay min)		
		Irr	Si (silicate)		
1812	PFG	Irr/aggl	Fe (iron oxide)	Plat/tab	Fe (iron oxide)
				Acic	Fe (iron oxide)
				Tab	Ba, S (barium sulphate)

*Num* number, *raw mat* raw material, *SFG* soft fine-grained, *BFG* banded fine-grained, *HFG* hard fine-grained, *CG* coarse-grained, *FS* ferruginous sandstone, *PFG* platy fine-grained, *irr* irregular, *plat* platy, *aggl* agglomerate, *subcirc* subcircular, *acic* acicular, *ang* angular, *tab* tabular, *clay min* clay mineral.

*Interpretation in brackets.

**Figure 5:**
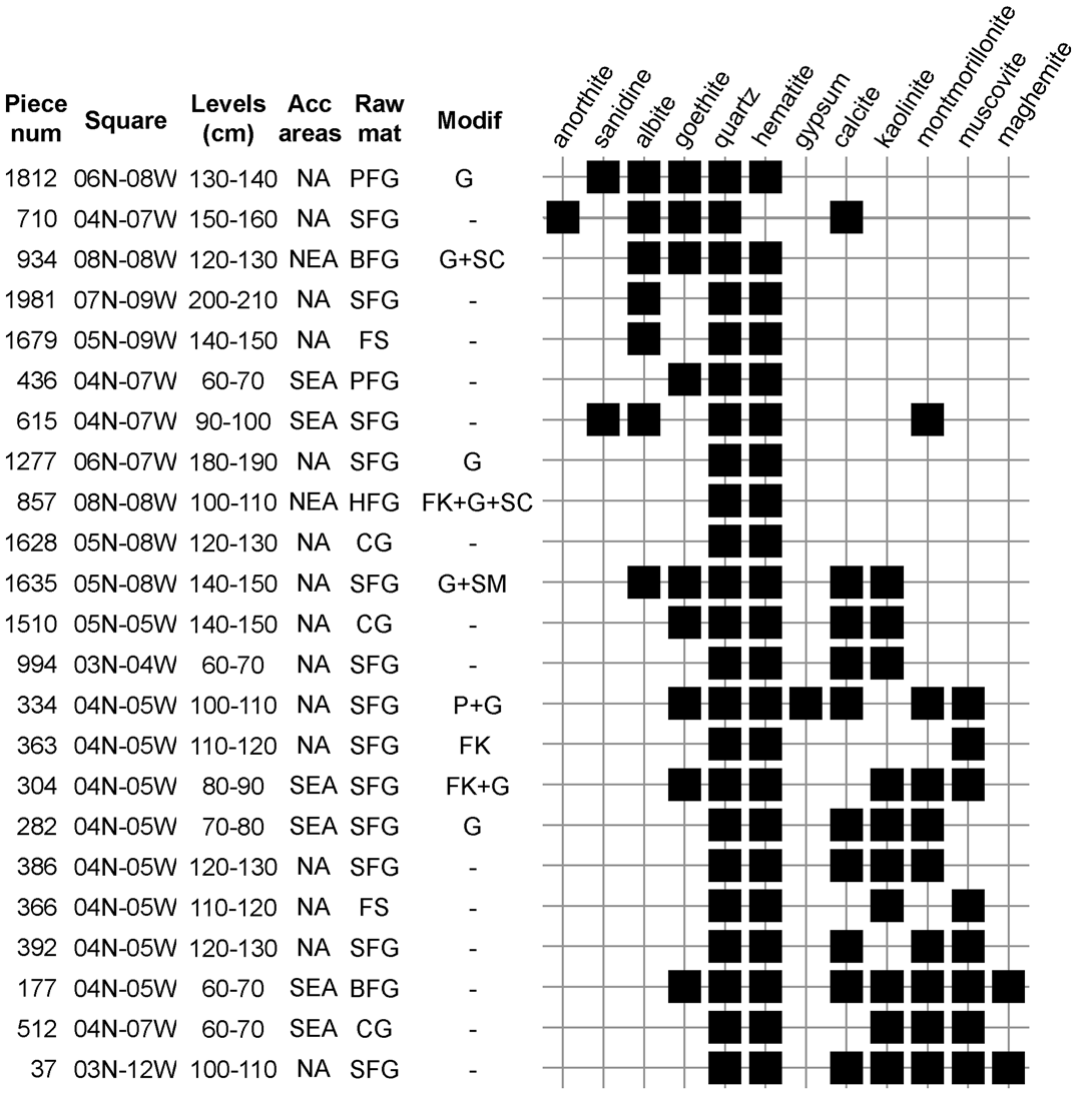
Seriation analysis of minerals identified by XRD and µ-XRD on ochre pieces from Porc-Epic Cave. *Num* number, *acc* accumulation, *mat* material, *modif* modifications, *SEA* southeastern area, *NEA* northeastern area, *NA* not applicable, *SFG* soft fine-grained, *BFG* banded fine-grained, *HFG* hard fine-grained, *CG* coarse-grained, *FS* ferruginous sandstone, *PFG* platy fine-grained, *FK* flaking, *SC* scraping, *G* grinding, *SM* smoothing, *P* pitting.

**Figure 6:**
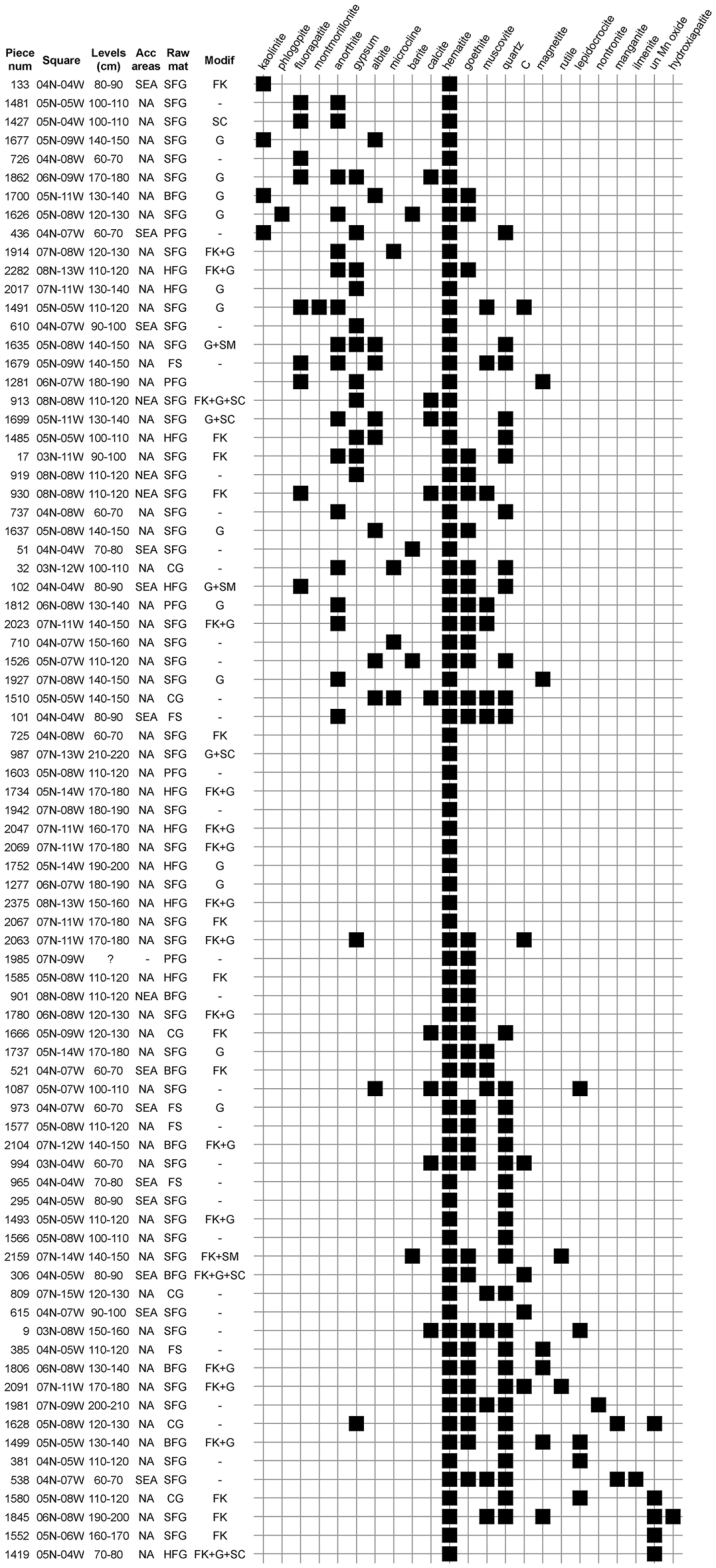
Seriation analysis of minerals identified by micro-Raman spectroscopy on ochre pieces from Porc-Epic Cave. *Num* number, *acc* accumulation, *mat* material, *modif* modifications, *SEA* southeastern area, *NEA* northeastern area, *NA* not applicable, *SFG* soft fine-grained, *BFG* banded fine-grained, *HFG* hard fine-grained, *CG* coarse-grained, *FS* ferruginous sandstone, *PFG* platy fine-grained, *FK* flaking, *SC* scraping, *G* grinding, *SM* smoothing, *C* carbon, *un* undetermined.

XRD, µ-XRD (Fig. 5, S6 Supplementary Table 1, S6 Supplementary Fig. 1) and µ-RS analyses (Fig. 6, S6 Supplementary Tables 2, 3, S6 Supplementary Fig. 2) indicate that hematite, goethite and quartz are found in almost all pieces. Additional iron oxides include magnetite, maghemite, ilmenite and lepidocrocite. The other minerals that were most often identified using XRD and µ-XRD are calcite, kaolinite, montmorillonite, muscovite, albite, and more rarely sanidine, anorthite and gypsum. Using µ-RS, anorthite, muscovite, gypsum, albite, calcite, fluorapatite, magnetite, and, to a lesser degree, undetermined Mn oxides, barite, kaolinite, microcline, manganite, rutile, hydroxiapatite, ilmenite, montmorillonite, nontronite and phlogopite were also identified.

### Relationship between elemental composition and macroscopic ochre types

The PCA exploring the variability of the ochre pieces’ elemental composition (Fig. 3a) identifies clear differences between raw materials identified on macroscopic criteria (Supplementary Information S4), but also marked overlapping.

Measurements for CG and HFG on the one hand, and for FS and HFG on the other hand, cluster separately. Pieces attributed to CG exhibit a relatively high content of Ti and to a lesser degree Si, which distinguishes them from measurements obtained on pieces attributed to HFG, characterised by a higher content in Fe, As and V. Conversely, FS markedly differs from HFG for its higher content of Ca, K and Si and a lower content in Fe, As and V. A clear overlap is observed in the composition of SFG and BFG. Such a similarity does not come as a surprise considering that the distinction between these two raw materials is only the presence of coloured bands in BFG. In contrast, a marked overlap is seen between measurements obtained on pieces attributed to PFG with CG, SFG, HFG and to a lesser extent FS. The above pattern indicates that some raw materials identified qualitatively on the basis of their colour, texture, hardness, inclusions and density correspond to rocks that show clear differences in elemental composition, while other raw material types encompass rocks of distinct elemental composition.

This implies that by choosing some raw materials, Porc-Epic visitors were able to predict the properties of the collected ochre for the functions for which it was intended, while in other cases this may have been more problematic.

### Elemental composition of ochre by layer and accumulation area

The compositional variability of the archaeological pieces by depth identifies an increased range of variation moving from the lower to the upper layers (Fig. 3e). Such increase does not appear to be due to sample size. In spite of being much less numerous, measurements on objects from 60 to 100 cm deep layers display a broader compositional variation and a tendency to contain, comparatively, more Ca and Si rich ochre, and less Fe and As than those from lower layers. This is consistent with results of Kruskal–Wallis tests, that indicate that the presence of Ca (K–W chi-square = 8.915, p-value = 0.0115), Si (K–W chi-square = 13.414, p-value = 0.001223), Fe (K–W chi-square = 9.5647, p-value = 0.008376), and As (K–W chi-square = 23.925, p-value = 6.379e−06) at different depth intervals (60–100; 100–90 and 190–280 cm) differs significantly. A noticeable difference also appears between accumulation areas. While the variability of ochre composition from SEA largely overlaps with that from other areas, the NEA includes pieces with a higher content of K, Fe and As that fall outside the compositional variability (see below) of the raw material available locally (Fig. 3f).

### Elemental composition of ochre by modification techniques

Pieces bearing no traces of modification show a higher degree of compositional variability that entirely encompasses that of modified pieces (Fig. 3b,d). The latter are comparatively richer in Fe and, among trace elements, As, V and Y. They have a lower content of Si, Ti, Rb and Sr. Although a marked overlap is observed when exploring the relationship between elemental composition and type of modification, pieces with traces of grinding or grinding associated with other modification types are those richer in Fe, V, and As. The ochre pieces with the highest proportion of Si and Ti do not bear traces of modification or only exhibit flaking. In summary, good quality raw materials, richer in Fe, were processed using a variety of techniques, while coarse-grained ochre, richer in Si, were left unmodified or were modified with techniques such as pounding that left diagnostic marks of percussion and flake scars on some of them, or no archaeologically visible evidence when the ochre was fully powdered. Interestingly, many good quality pieces are unmodified, suggesting that raw materials of all types and qualities were brought to the site and curated or disposed of.

### Elemental composition and texture

SEM–EDS analyses reveal to a degree that each raw material identified according to morphological features bears diagnostic textural and compositional features (Fig. 4, Table 2, S5 Supplementary Fig. 1, S5 Supplementary Table 2). For a detailed description of each raw material type see Supplementary Information S5. The features that enable differentiating ochre types are: (1) the proportion of Fe and the morphology of iron oxide particles and (2) the proportion, size and shape of associated particles, particularly coarse Si-rich grains, but also feldspars, micas, barium sulphates, calcium sulphates and clay minerals.

FS (Fig. 4s,t) and CG (Fig. 4q,r) differ from the other raw material types in that they are richer in Si grains. These are surrounded by agglomerates of Fe-rich grains, clay minerals and calcium sulphates. In CG, feldspars were also detected and Si grains are more variable in size and sometimes larger than that of FS.

HFG (Fig. 4n–p) and PFG (Fig. 4u,v) ochre pieces are characterised by a high proportion of Fe. In the case of HFG, they appear in the form of agglomerates of submicrometric iron oxides and in the case of PFG, in the form of agglomerates of platy Fe-rich grains, comparable to those characteristic of specularite.

Finally, SFG (Fig. 4a–k) and BFG (Fig. 4l,m) differ from the rest of raw material types due to the presence of agglomerates of Fe-rich, usually submicrometric grains, mixed with clay minerals and other features such as silicate grains, smaller than those observed in CG. Some of the SFG pieces also contain feldspars, micas, Ba sulphates and calcium sulphates.

In sum, differences between ochre types, established in previous studies based on macroscopic features, match variations in elemental composition and microscopic texture. Clayish Fe-rich ochre types (SFG, BFG), ochre types with very high proportions of Fe (HFG, PFG) and Si/Ti–rich (CG and FS) ochre types can be clearly distinguished when combining elemental and morphological data. This shows that the composition of ochre played a role in technological and functional choices and that, by selecting different types of ochre, Porc-Epic inhabitants could in some cases predict the properties of the ochre.

### Mineralogical composition by raw material, modification techniques and layers

Seriation analyses of minerals detected by XRD and µ-XRD (Fig. 5) and µ-RS (Fig. 6) in the ochre pieces reveal that hematite, goethite and quartz are the only minerals present in almost all pieces (S6 Supplementary Tables 1–3). Associated with them are two cohorts of minerals that are only seldom found together. To the first group belong manganite, undetermined Mn oxides, fluorapatite, anorthite, sanidine, albite, nontronite and microcline. To the second group belong muscovite, calcite, kaolinite, montmorillonite and maghemite.

No clear associations are observed between mineral phases and the attribution of ochre pieces to a given raw material, nor between techniques used to modify ochre and minerals composing them (Figs. 5, 6). No detectable change in the mineral content of the ochres is observed throughout the stratigraphy.

The multivariate analysis of the elemental composition (Fig. 3b) shows that coarse ochre containing Si-rich grains was preferentially processed by crushing the ochre, which has occasionally left traces of flaking on the pieces. However, this pattern does not appear when investigating the correlation between modification techniques and phase composition because quartz is also present in finer-grained raw materials in the form of smaller particles.

The presence of Si-rich minerals in the ochre pieces is not necessarily the result of a deliberate choice since quartz is found in all natural samples analysed by µ-XRD (S6 Supplementary Table 1) and is present in high proportions in the ochre pieces from the wadi Laga Dächatu (S2 Supplementary Fig. 1) analysed using EDXRF (see below). Considering that the more intensively modified and fine-grained ochre pieces are those with low Si content, this pattern indicates that Porc-Epic inhabitants were looking for pieces with a high content in hematite and goethite and a relatively low content in quartz.

### Elemental composition of locally available ochre

EDXRF analyses (Table 1, S5 Supplementary Table 1) show that ochre available locally is rich in Si and also contains Fe, K, Ca, Ti as major or minor elements. As observed for the archaeological pieces, Mn is present as a minor or trace element. V, Cr, Ga, As, Rb, Sr, Y and Ba are present as trace elements.

The PCA exploring differences between the elemental composition of natural and archaeological ochre (Fig. 3c) shows that a large proportion of the natural pieces do not fall within the variability of the archaeological pieces. Most natural ochre is characterised by a higher content in Ti and Si. However, part of the measurements taken on natural pieces overlap with the FS and CG archaeologically documented ochre types. A large part of PFG, SFG and BFG fall outside the variability of the natural pieces, and HFG pieces cluster apart.

Most modified pieces, rich in Fe, As and Y, clearly differ in composition from the natural pieces. Among the archaeological pieces whose composition matches that of the natural pieces, most are unmodified (Fig. 3d). They come indistinctly from different archaeological levels (Fig. 3e). Some were found in the SEA accumulation, but none comes from the NEA accumulation (Fig. 3f). This, however, may be due to sample size.

Results show that coarse-grained ochre, richer in Si and Ti, may have been collected by Porc-Epic Cave inhabitants in erosional deposits close to the site, or in the upstream formations from which they originate. However, most of the good quality Fe-rich ochre, particularly pieces that were processed by grinding, found in the NEA accumulation and in the lower layers, does not match the composition of natural ochre available locally. This could either imply that (1) during the MSA the drainage basin of the wadi differed from the present and that, as a result, finer-grained Fe-rich ochre was available locally or that (2) good-quality Fe-rich ochre not available locally was collected at distant sources by Porc-Epic inhabitants and brought to the site or traded with human groups who had access to those sources.

### Composition of ochre residues on ochre processing tools and ochre-stained artefacts

SEM–EDS and mineralogical analysis from eleven ochre processing tools (OPT) or ochre-stained artefacts (OSA)^29^ revealed that six (OPT 3, 6, 7, 9, 12, 13) include element associations and morphological features indicating the presence of a single type of ochre per tool; five (OPT 1, 2, 4 and OSA 5, 15) display associations suggesting the presence of two types of ochre (Supplementary Information S3).

Three main groups of ochre residues were identified on the OPT and OSA at Porc-Epic Cave^29^. The first group (identified on OPT 1–4, 12, 13, and OSA 5 and 15) is characterised by the presence of agglomerates of submicrometric or acicular Fe-rich grains, sometimes also rich in Ti, or large Fe aggregates. They are often mixed with clay minerals, sometimes feldspars, and systematically feature quartz grains. Residues belonging to this category, particularly those that are richer in Fe (OPT 1 and 3), are similar in composition to the SFG and BFG ochre types, and possibly HFG. However, we cannot disregard that they may belong to coarse-grained ochre types FS or CG, particularly when rich in Ti, as in the case of OSA 15.

The second group (identified on OPT 7 and 9) features characteristic agglomerates of Fe-rich platelets that show clear similarities in morphology and composition to those identified on PFG ochre pieces.

The third group displays large iron oxide particles, sometimes composed of smaller platelets (OPT 6), also is rich in Ti (OPT 4 and OSA 15) and is in some cases associated with clay minerals (OSA 5). The coarser nature of these particles and the presence of Ti seem to match CG and FS ochre types, but it could also represent an extreme in variation of ferruginous clay rocks (such as SFG or BFG).

As indicated elsewhere^29^, no obvious relationship is observed between the type of processed ochre and the raw material from which the ochre processing tools are made. However, residues identified on OSA 5 provide interesting data on which type of ochre was potentially used for symbolic purposes. Our analysis shows that ochre covering this object includes at least two types: ferruginous clay rocks, likely belonging to good-quality Fe-rich ochre types (such as SFG, BFG or HFG), and isolated coarse Fe-rich particles, probably belonging to the coarsest types of ochre (such as CG or FS). This implies that a complex ochre recipe, requiring a mixing of different ochre types, probably to obtain a specific colour or texture, was used for symbolic purposes, and that both good quality Fe-rich ochre and coarser-grained ochre may have played a role in these activities.

## Discussion

Porc-Epic Cave represents one of the few Palaeolithic sites with a long stratigraphic sequence, spanning a period of at least 4500 years, that has yielded a continuous and extensive record of ochre use. Its analysis is therefore key to document cultural practices associated with the selection, transport, processing and use of these materials, explore diachronic changes in these domains, and propose hypotheses for their technological, cultural, and cognitive drivers.

The chemical characterisation of Porc-Epic ochre confirms that MSA inhabitants collected a wide variety of ochre types, and brought them to the site to produce ochre powder of different textures and shades, probably in accordance with different activities. The ubiquitous presence of hematite, and to a lesser degree goethite and other iron oxides in all samples indicates hematite is the mineral Porc-Epic inhabitants were the most interested in when collecting ochre in the environment or exchanging it with neighbouring populations. Comparison between the elemental variability of ochre available in the local environment and ochre found at the site (Fig. 3c–f) identifies three categories: (1) coarse-grain pieces containing a high proportion of Si, Ti and a low proportion of Fe, available in the vicinity, but not considered suitable by the site inhabitants when collecting ochre close to the site, (2) ochre presenting, albeit to a lesser degree, these same characteristics but richer in Ca and K, available locally, and collected and brought to the site, (3) mostly fine-grained ochre rich in iron oxides, As, Y, V and occasionally K and Ca, very rare or absent locally, and probably collected from distant sources or traded with neighbouring groups. Available information on the evolution of the Laga Dächatu drainage basin does not identify substantial changes to the eroded geological formations upstream of the site^30–33^. This suggests that the third ochre category was probably rare. The ochre pieces included in this category are more friable and less prone to survive fluvial transport, a supplementary reason for thinking that they were collected at primary sources or close to them. Although there is no unambiguous association of modification techniques and ochre types, and many pieces were modified with multiple techniques, the pieces belonging to the third category are those that more often bear traces indicating that they were abraded on a grindstone, or scraped, to produce a fine powder. Those belonging to the second category more often bear traces of having been crushed or are unmodified, the latter likely representing objects curated to be modified with this technique, which may leave little or no archaeological trace when pursued to exhaustion. The third category was more intensively sought at the beginning of the site occupation, and particularly during one of them (NEA), and less so toward its end. Such a gradual tendency is paralleled by a gradual decrease in the application of grinding and scraping, and an increase in flaking/crushing.

Identified by bringing into play the stratigraphic origin and spatial distribution of the ochres, their appearance and colour, the techniques used to modify them, the elemental composition of archaeological and natural ochre, the trends revealed by these complementary data depict a technical system in gradual evolution. This technical system has the peculiarity of not losing during its evolution any of the characteristics present at its beginning. It has the particularity of excluding some local raw materials and using some exotic ones, probably more difficult to obtain. It entailed the storage of all types of raw materials on site, an indication of their continuous, perhaps habitual, use. It is also characterised by the application to the selected raw materials of techniques appropriate to each one, while maintaining a high degree of flexibility in this application. The resulting picture is that of a culture in slow transition, gradually replacing to some degree exotic high-quality rocks with more locally available ones and applying to them the techniques best adapted to their exploitation. This gradual transition could be the consequence of an increasing need for coarser powder linked to the importance of the related activities or of a reduced access to high quality ochre due to a reduction in the mobility of the group. Identifying for what purpose these different ochre types were used is a notoriously elusive endeavour. However, the comparison of the chemical composition of ochre residues identified on OSA 5^29^ with that of ochre pieces identified on the site brings new interesting data on which type of ochre may have been used for symbolic purposes. Residues identified on this object include both clayish Fe-rich ochre and ochre similar to coarser Ti–rich ochre types. This could mean that complex compounds, produced by mixing different types of ochre to obtain specific colours and textures, were used for symbolic purposes.

Recent studies^1^ have defined three phases of increasing ochre use in the African continent during the MSA: initial (500–330 ka), emergent (330–160 ka) and habitual (160–40 ka). During the latter, the use of ochre becomes regular and systematic in large regions of the African continent. However, our results indicate that during this time span, there may have been differences in the way ochre was processed and used. Although situated at the end of this trajectory, the Porc-Epic record reflects a deeply-rooted yet constantly evolving ochre culture. These variations still need to be fully explored at other sites in order to understand the mechanisms behind the transmission of cultural practices related to ochre use.

## Methods

Our study concerns the ochre assemblage recovered at Porc-Epic Cave during the 1975–1976 excavation campaigns^25^. Here we define the term “ochre” as a variety of rocks, from soil lumps to ore minerals, containing a high proportion of iron oxides, and characterised by yellow, orange, red or brown colours or streaks^9,15,19,34–36^. This assemblage includes 4213 ochre pieces (39.97 kg) kept at the National Museum of Ethiopia in Addis Ababa, analysed by one of us (DR) between 2011 and 2014. Approximately 10% of the ochre pieces (n = 421) lack contextual information and were excluded from the analysis. Our study also includes 40 natural ochre pieces collected by 8 people during a three-hour survey, over a distance of 3 km along the upstream course of the c. 50 km-long wadi Laga Dächatu, above which is situated the cave opening (S2 Supplementary Fig. 1). The collected ochre nodules show a rounded morphology indicating that these pieces were eroded from upstream formations into the bed of the wadi. The Ethiopian Authority for Research and Conservation of Cultural Heritage (ARCCH) issued us with permits to study the material, temporarily export ochre pieces and analyse loose microfragments (1–3 mm in length) present in the plastic bags containing each ochre piece (permit numbers and issue dates: 11/KT-34/011, 01/01/2013; 011/KT-41/066, 09/01/2013; 011/KT-120/008, 10/01/2013 and 011/17-26-2/007, 16/01/2014).

### Macroscopic observation and technological analysis

We recorded contextual, technological and morphometric information on 3792 ochre pieces: square, 10 cm spit in which the object was found, length, width, thickness and weight of complete objects, raw material type (S4 Supplementary Table 1), colour and morphology of the piece (slab, pebble, nodule, irregular). Anthropogenic modifications (traces of flaking, striations, facets, smoothed areas, incisions, pits) were identified macro- and microscopically based on experiments conducted on local raw material and the literature^19,20,37,38^. For precise descriptions of each modification, see Supplementary Information S4 and S4 Supplementary Fig. 1. All ochre types and anthropogenic modifications were photographed with a Leica Z6 APO macroscope.

### Chemical characterisation of ochre pieces

In order to determine the composition of the Porc-Epic ochre, we selected 92 ochre pieces representative of the raw materials and modifications recorded in the whole collection (Supplementary Information S7). This sample includes objects found throughout the stratigraphy. Analyses were also conducted on 40 natural ochre pieces collected in erosional deposits close to the site that originated from upstream formations.

Non-destructive surface analyses (micro-Raman spectroscopy, scanning electron microscopy coupled with energy dispersive X-ray spectroscopy and X-ray fluorescence spectrometry) were conducted on fresh fractures or flat surfaces, avoiding areas covered with patina or concretions. When necessary, sediment was removed from the surface with a soft brush, leaving no marks or striations on the pieces. Analyses requiring powder samples (X-ray and micro X-ray powder diffraction) were only conducted on loose ochre microfragments originally attached to larger ochre pieces present in the same plastic bags. Sampling therefore did not affect or modify the original ochre pieces from which the microfragments came.

Results of chemical analyses were compared with previously published data on technology, raw material and stratigraphic provenance^20,28,29^. Elemental and structural analysis of ochre residues from nine ochre processing tools (OPT) and two ochre-stained artefacts (OSA) were compared to the composition of ochre pieces coming from the same archaeological layers^29^. Analyses conducted on ochre residues from OPTs and OSAs, published elsewhere^29^, are indicated in Supplementary Information S7 and results of the analyses are presented in Supplementary information S3. Details on the conditions of the analyses can be found elsewhere^29^.

### Elemental analysis

The elemental composition was determined by using two non-destructive methods: scanning electron microscopy coupled with energy dispersive X-ray spectroscopy (SEM–EDS) and portable energy dispersive X-ray fluorescence spectrometry (EDXRF). SEM–EDS allows one to view and record images of the morphology of analysed areas, observe how different components are arranged by using elemental mapping, and estimate the relative abundance of elements. EDXRF identifies and quantifies major, minor and trace elements in a fast and effective way, enabling the analysis of many pieces.

SEM–EDS analyses were conducted on 22 archaeological pieces using a FEI Quanta 200 with SiLi detector, and SDD-EDAX detector. EDS analyses were performed at the same working distance (10 mm) and with the same acquisition time (100 s). Backscattered electron images (BSE) and elemental analyses were obtained under a low vacuum mode with an accelerating voltage of 10 kV and 15 kV.

EDXRF measurements were carried out on 80 archaeological ochre pieces and 39 natural ochre pieces from the wadi Laga Dächatu using an Ametek portable SPECTRO xSORT X-ray fluorescence spectrometer equipped with a silicon drift detector (SDD), a low power WX-ray tube with an excitation source of 40 kV and an X-ray beam of 8 mm. Measurements were performed with a constant working distance by setting the spectrometer on a lead receptacle. Four to five measurements were taken on each of the archaeological pieces and one to six on natural ochre samples with a spectra acquisition time of 60 s. The spectrometer is internally calibrated by an automated measure of the elemental content of a standard metal shutter. A supplementary calibration^22,39^, based on the Lucas-Tooth and Price methodology^40^ was applied. This calibration, developed with X-LabPro software (Ametek, Berwyn, USA) adjusts the mass attenuation coefficient and calibration slopes for major and trace elements by using certified standards and reference samples analysed by ICP-OES and ICP-MS^41^. Results of EDXRF measurements were treated with CoDaPack software^42^. This software treats raw concentration data by replacing values below the detection limit^43^ and performing centred log ratio transformation (clr)^44^. We performed principal component analyses (PCA) using PAST v. 4.08 software^45^ for ten major, minor and trace elements (Si, K, Ca, Ti, Fe, V, As, Rb, Sr, Y). Elements with more than 10% of measurements below the limit of detection (Ba, Cr, Ga, Mn) were not taken into account. In the PCAs, we plotted individual measurements instead of means for each analysed piece considering that some raw materials are not homogeneous and EDXRF measurements were taken in different areas to account for such variability. A Kruskal–Wallis test was conducted to compare the EDXRF measurements from pieces found at different depth intervals (60–100; 100–90 and 190–280 cm) using R v. 4.2.2 software^46^.

### Structural analysis

To characterise the mineral composition of the ochre from Porc-Epic, we conducted both micro-Raman (µ-RS) and X-ray (and micro X-ray) powder diffraction analyses (XRD and µ-XRD). The first technique allows one to characterise single grains. Since it is a non-destructive method, we analysed all (n = 80) exported pieces. The second method allows a more comprehensive identification of minerals, especially when conducted on powder samples.

µ-RS analyses were conducted on 80 ochre pieces using a SENTERRA Dispersive Raman Microscope (Bruker), equipped with an internal calibration system. The working area was examined through the integrated colour camera. Spectra were acquired with a 785 nm laser, in a spectral range from 50 to 1500 cm^−1^. Laser power varied from 1 to 10 mW in order to avoid thermal transformation of mineral phases. The integration time was set to 20 s, with a number of co-additions ranging between 20 and 40 depending on the presence of fluorescence radiation and signal-to-noise ratio. In order to identify mineral phases, OPUS 7.2. software (Bruker, Germany) was used to compare the obtained spectra with those from the RRUFF spectra library^47^.

XRD analyses were performed on twelve loose ochre microfragment samples ground with an agate mortar. Nine were analysed using a PANalytical X’pert MPD-PRO diffractometer (Bragg Brentano Theta-Theta geometry) equipped with a graphite secondary monochromator and a copper anticathode (mean lambaKalpha = 15,418 Å). The working tension and intensity were set at 45 kV and 40 mA, respectively, and the time of analysis was of 6.47 h and 14.56 h, depending on the sample. Additional XRD surface analyses were conducted on three samples (samples 436, 1277 and 1628) using a PANalytical X’Pert-Pro MPD Diffractometer system (Bragg Brentano geometry, Cu anticathode, KAlpha1 and KAlpha2 rays), with a diffraction angle 2θ from 5° to 70° and analyses set to 11 min. Non-destructive μ-XRD surface analyses were conducted on 11 archaeological ochre pieces and six natural ochre pieces found along the wadi Laga Dächatu using a C2RMF laboratory-made μ-XRD device (Rigaku monochromatic source, λ = 1.54186 Å, 200 μm collimator). Mineralogical phases were identified by using the routine DIFFRAC.SUITE™ EVA software package (Bruker AXS GmbH, Germany), combined with the specific powder diffraction file (PDF2) database (International Centre for Diffraction Data—ICDD, Pennsylvania, USA) and the Match! © software (version 3.8.3.151, Crystal impact, Bonn, Germany).

Seriation of a presence-absence matrix of minerals (columns) identified by XRD, µ-XRD and micro-Raman spectroscopy was performed using PAST v. 4.08 software^45^. The seriation routine reorganised the data matrix such that the occurrences are concentrated as much as possible along the diagonal. We used the unconstrained mode, where both rows and columns are free to move.

### Supplementary Information

Supplementary Information.

## Data Availability

Most data supporting the findings of this study are available within the paper and its Supplementary Information file. The rest of the data produced and used in this paper are available from the corresponding author upon reasonable request.
