# Supplementary material for: First identification of an evolving Middle Stone Age ochre culture at Porc-Epic Cave, Ethiopia
Source: Sci Rep. 2023 Sep 8;13:13261. doi: 10.1038/s41598-023-39957-y (PMC10491814; doi:10.1038/s41598-023-39957-y)
Supplement: Supplementary file 1 — Supplementary Information. [file 41598_2023_39957_MOESM1_ESM.pdf]

Supplementary online material

First identification of an evolving Middle Stone Age ochre culture at Porc-Epic Cave, Ethiopia

Daniela Eugenia Rosso[1,2*], Martine Regert[2], Francesco d'Errico[3,4]

[1.] Departament de Prehistòria, Arqueologia i Història Antiga, Grupo de Investigación Prehistoria del Mediterráneo Occidental (PREMEDOC), Universitat de València, Valencia, Spain.

[2.] Université Côte d'Azur, CNRS, CEPAM, Nice, France.

[3.] PACEA UMR 5199, Université de Bordeaux, CNRS, Pessac, France.

[4.] SFF Centre for Early Sapiens Behaviour (SapienCE), University of Bergen, Bergen, Norway.

* Corresponding author
Email: daniela.rosso@uv.es

Table of contents

S1 - Theoretical background and early pigment use in eastern Africa 2
S2 – Archaeological context 5
S3 – Previous research: ochre processing tools 9
S4 – Previous research: ochre pieces 14
S5 – Ochre pieces: elemental data 20
S6 – Ochre pieces: mineralogical data 32
S7 – Methods 42
Supplementary references 46

S1 - Theoretical background and early pigment use in eastern Africa

Over the past few years different criteria have been proposed to assess the behavioural complexity of early hominins [1–3]. Among these, systematic use of ochre is considered as a key behavioural marker to infer cultural complexity [4–6]. Many interpret the use of ochre as a proxy for the origin of symbolic thinking [4,6–17]. However, this view remains controversial as ochre may also have been used for utilitarian functions such as an ingredient in adhesives, a constituent of hide tanning compounds, an insect repellent, a sunscreen or an antiseptic [18–31]. Today, the idea that the earliest use of ochre fulfilled multiple functions, supported by ethnographic evidence [32,33], has become widespread in the archaeological literature, though criteria to distinguish functional from symbolic uses or establish that both were in existence remain largely elusive [1,34].

Evidence for ochre processing is reported in numerous Middle Stone Age (MSA) sites from Africa [1,3,7,10,13,35–43] and Middle Palaeolithic sites from Europe [17,44–48] and Western Asia [49–52]. In East Asia, only a few sites have yielded ochre pieces older than 50,000 years [53,54] and some of these instances still need an in-depth evaluation to verify their anthropogenic origin. Recent discoveries have revealed clear evidence for ochre processing around 40 ka in this region [55].
However, as already mentioned, the way in which ochre was used at early sites and the role it played in these societies remains unclear. This is partly due to the rarity of comprehensive analyses of ochre collections. Only few studies combine technological and morphometric analysis with physico-chemical characterisation of ochre, and even fewer include the study of associated ochre-stained objects and artefacts [13,37,56–59]. This problem is particularly evident in eastern Africa, where systematic studies of ochre assemblages have only started to be conducted in the past few years [7,60,61].

The earliest possible evidence for pigment use in eastern Africa comes from the sites of Gadeb, Ethiopia [62] and Olduvai Gorge, Tanzania [63], and dates back to the Early Stone Age, around 1.5-1.0 Ma BP. By "eastern Africa", we refer to the area including modern-day countries of Eritrea, Ethiopia, South Sudan, Sudan, Somalia, Djibouti, Kenya, Uganda and Tanzania [64,65]. At Gadeb, Ethiopia, evidence consists of fragments of weathered basalt producing red streaks [62]. At Olduvai Gorge, Tanzania, two lumps of red

ochre were, according to the discoverer, clearly brought to the site by *Homo erectus* [63]. At Garba I, Melka Kunture, Ethiopia, an area of two to three square metres, identified in levels dated to c. 500 ka BP, was stained red and associated with small elongated and rounded ochre lumps [66]. In the Kapthurin Formation, GnJh-15 site, Kenya, >70 pieces of red ochre, with a weight of >5 kg and possible grindstones stained with ochre were reported in layers dated 500–284 ka [3,65,67,68]. Excavations conducted at Olorgesailie, Kenya, have revealed the presence of 86 lumps of potential black pigment and two red ochre pieces in levels dated from ~320 to ≥295 ka [7]. Geochemical analyses show that these rocks are exogenous. The red pieces bear striations produced by grinding. One shows anthropogenic notches and the other a possible perforation attempt.

MSA levels of site Sai 8-B-11, at Sai Island, Sudan [69,70], dated to c. 180 ka, yielded yellow and red ochre pieces associated with ochre processing tools (sandstone mortars shaped by knapping and small chert pebbles that show the presence of yellow and red residues). From 160 ka, systematic use of ochre becomes ubiquitous [42,43]. Numerous late MSA sites from eastern Africa have yielded ochre pieces that are sometimes associated with grindstones. Their number is probably underrepresented. Interest in pigment use is relatively recent, and so it is likely that an undetermined number of finds were not systematically reported in the past. Except for a few cases, ochre assemblages from this region are only briefly described in the literature.

In Tanzania, evidence for MSA ochre use was found in Mumba [65,71–75] and Nasera rock shelters [65,74,76]. In Mumba, it consists of worked pieces of yellow and red ochre from levels dated to c. 100–50 ka [71]. Grindstones were also found at both sites but it is unclear whether they preserve ochre residues. At Kisese II Rockshelter, Tanzania, red and more rarely orange and yellow ochre was identified in levels >45 ka cal BP [77–79]. Ochre pieces showing wear-facets were also recovered from levels dated to >43 ka cal BP. “Stone palettes” with ochre-stained surfaces are reported in levels dated 39.6–34.3 ka cal BP.

Recent excavations at Panga ya Saidi, Kenya, have revealed the presence of 17 ochre fragments probably used to produce ochre powder in layers dated to c. 48–14.5 ka [41,60]. Two of them, recovered from layers dated c. 48–25 ka, bear traces of modification. Two flakes with traces of red ochre and one small ochre-stained lower grindstone were recovered at Enkapune Ya Muto, Kenya, in levels probably older than 41,400±700 BP [80]. At Mochena Borago Rock Shelter, Ethiopia [81,82], ongoing research [83] has revealed the presence of more than 260 red, yellow, pink, purple, and brown ochre pieces, weighing nearly two kilograms, associated with ochre processing tools, in levels dated between c.

53 and 38 ka cal BP. More than half bear traces of modification, including rubbing, grinding and less frequently scoring and engraving marks. Red and grey pigments were found in MSA levels at Gorgora Rock Shelter, Ethiopia [84–86]. The presence of ochre was also reported at Aduma [87] and Goda Buticha [88–90], Ethiopia. Porc-Epic Cave, Ethiopia, has yielded the largest late MSA ochre assemblage in the region [61,91,92], which is the subject of the present study.

S2 – Archaeological context

Porc-Epic Cave is located 3 km south of Dire Dawa in Ethiopia, near the top of the Garad Erer hill, between the Afar Depression and the Somali Plateau (S2 Supplementary Fig. 1). It opens at the base of a Jurassic limestone cliff, 140 m above the wadi Laga Dächatu, whose main channel course has a length of over 50 km.

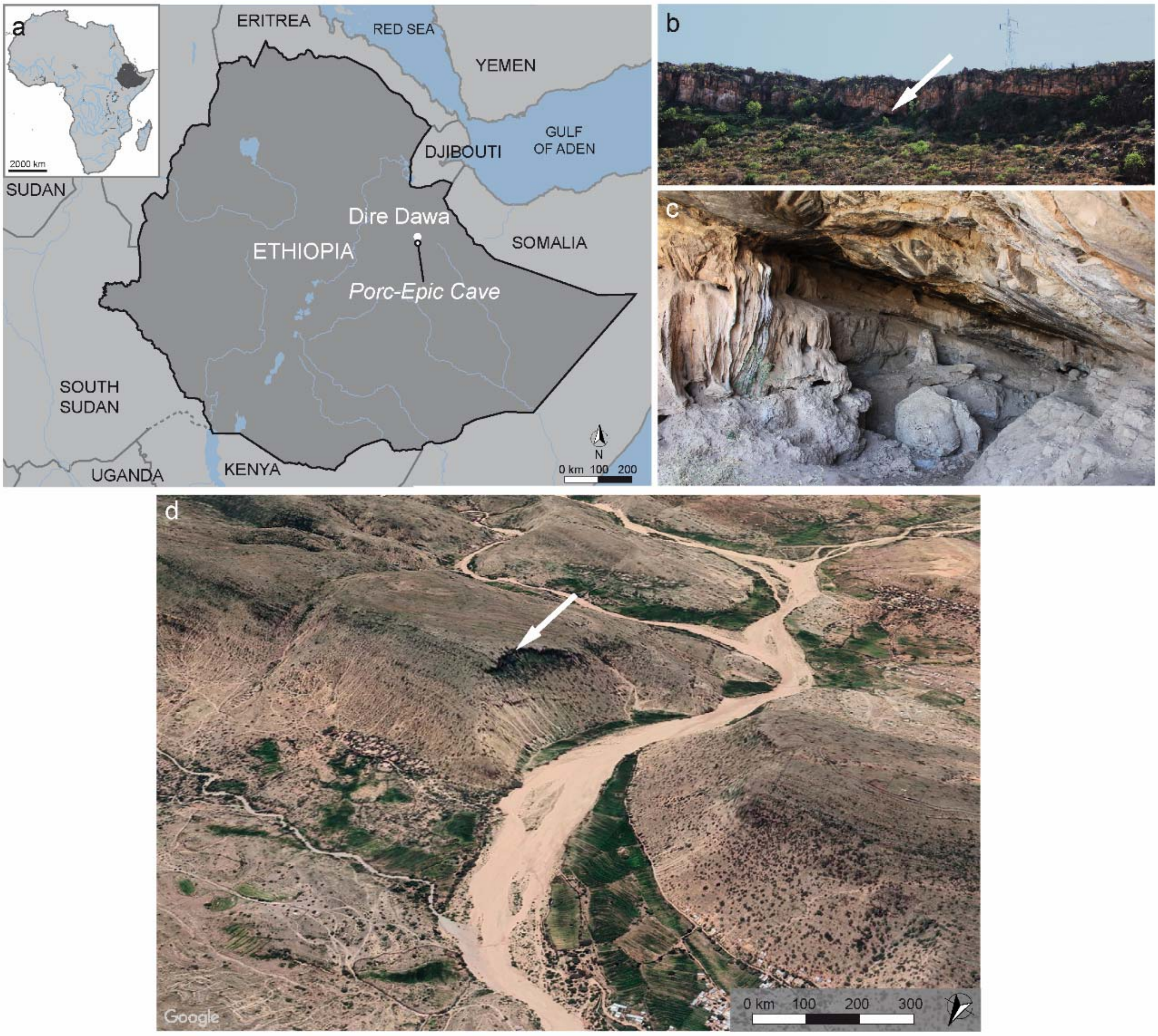

S2 Supplementary Figure 1. Location of Porc-Epic Cave.
(a) Location of the site. (b) View of the top of Garad Erer hill. The arrow indicates the cave entrance. (c) View of the cave (photo A. Herrero). (d) View of Garad Erer hill and wadi Laga Dächatu. The arrow indicates the cave entrance (copyright: Google Earth). Modified after [91] under a CC BY license, with permission from PLOS ONE, original copyright 2016.

The cave was discovered in 1929 by Pierre Teilhard de Chardin and Henry de Monfreid, who excavated a test pit to evaluate the archaeological potential of the site [93]. In 1933, Henri Breuil and Paul Wernert extended the excavation [94] and described the "later schematic style" of the cave's rock art [95,96]. John Desmond Clark excavated a trench in 1974 [95,97], and in 1975–1976 Kenneth D. Williamson directed an excavation over a surface of approximately 49 m^2 and a depth of 3 m. In 1998, fieldwork conducted by a team from the *Muséum National d'Histoire Naturelle*, Paris, France, and the Authority for Research and Conservation of Cultural Heritage (ARCCH) of Ethiopia helped clarify the Porc-Epic stratigraphy [98].

The stratigraphy of the cave comprises a succession of clayish levels, sandy levels and breccia and was divided into seven units (S2 Supplementary Fig. 2) that are described in detail elsewhere [92,95]. MSA material was recovered from levels 2, 3C/D and 4A/B [95], approximately 60 to 220–230 cm below datum. The overlying layers (6, 7A and 7B) are composed of fine sands and loam with interstratified hearth material containing LSA and Neolithic artefacts [99].

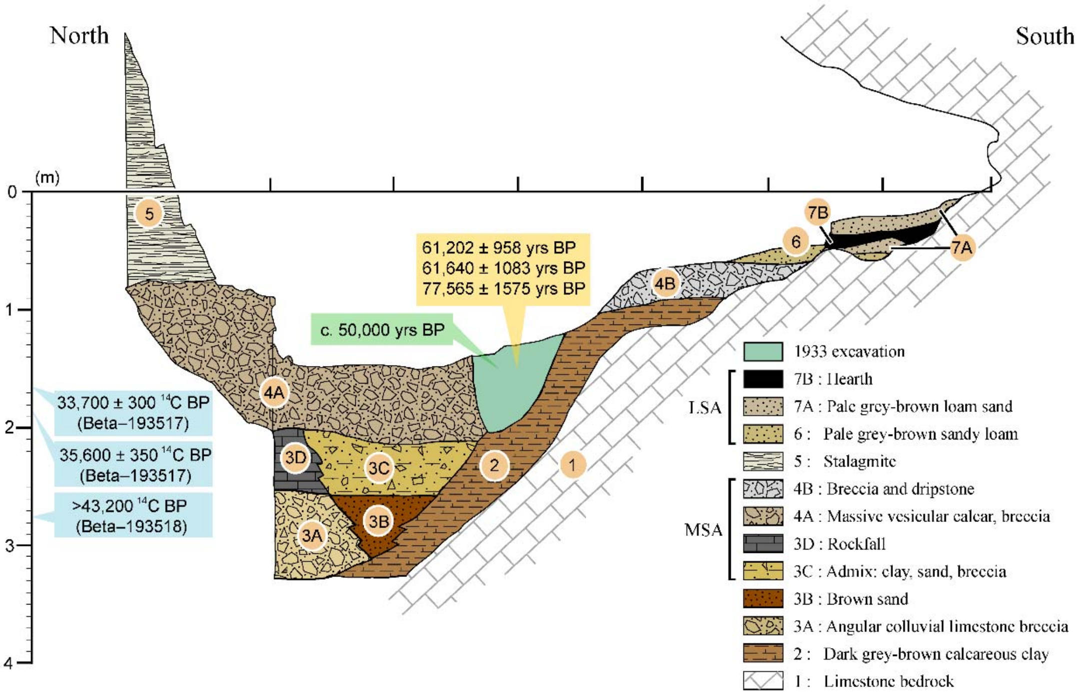

S2 Supplementary Figure 2. Porc-Epic Cave's stratigraphy.
Eastern profile (09W-10W) at the end of the 1974 excavation. The gamma-spectrometry age of a human mandible and the obsidian hydration ages for artefacts recovered during the 1933 excavation are indicated in green and orange, respectively. ^{14}C ages obtained from gastropod opercula are indicated in blue. Their stratigraphic position is approximate, as only the depth and square from which these objects were recovered is known, and therefore cannot be correlated with a specific layer. Reprinted from [91] under a CC BY license, with permission from PLOS ONE, original copyright 2016.

Different attempts have been made to date the sequence, but the chronology of the site remains unclear [92]. Three artefacts were dated by obsidian hydration to 61,202 ± 958, 61,640 ± 1083, and 77,565 ± 1575 [100], but this dating method is now considered unreliable [101,102]. High-resolution, low-background gamma-ray spectrometry analyses of a human mandible produced an age of c. 50 ka [88]. Accelerator mass spectrometry (AMS) radiocarbon determinations for three *Revoilia* gastropod opercula [99] yielded uncalibrated ^{14}C ages of 33,700 ± 300 (Beta–193517), 35,600 ± 350 (Beta–193516), and >43,200 (Beta–193518), suggesting that the sequence may have accumulated over at least 4,500 years [92,103]. The 95.4% probability range of the two finite ages [104] are 38,800–37,049 cal BP, and 41,084–39,421 cal BP (IntCal13; OxCal 4.2). The stalagmite that seals the breccia containing the main MSA levels yielded a ^{14}C age of 4,590 ± 60 BP and U–Th age of 6,270 ± 1020 BP [95]. Charcoal fragments recovered in the uppermost breccia have been dated to 5,700 ± 110 BP.

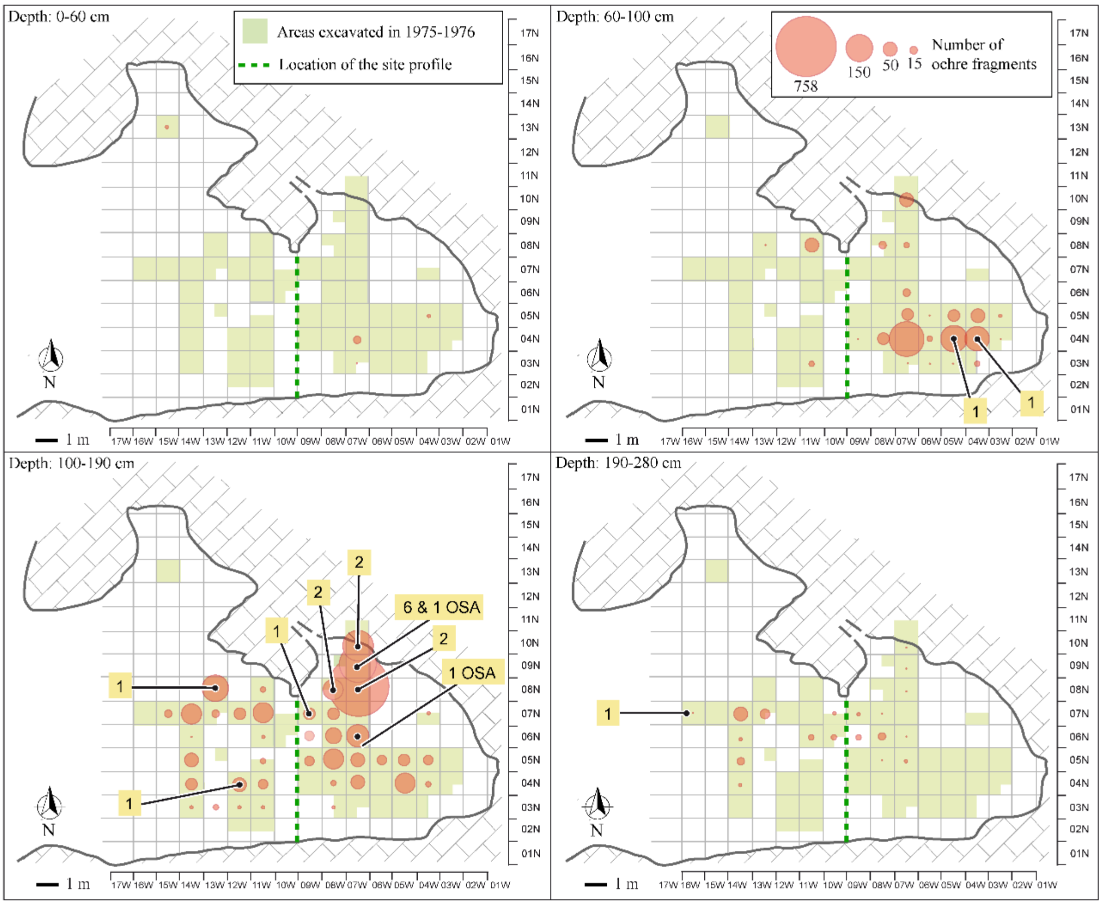

S2 Supplementary Figure 3. Spatial distribution of ochre pieces, ochre processing tools and ochre-stained artefacts.
Bubble sizes reflect the frequency of ochre pieces per grid unit. Numbers indicate ochre processing tools and ochre-stained artefacts (OSA) when indicated. Reprinted from [61] under a CC BY license, with permission from PLOS ONE, original copyright 2017.

Finds from the MSA layers at Porc-Epic Cave include stone tools [88,95,98,105–112], faunal remains [99,113,114], a human mandible [114,115] and gastropod opercula [103], ochre and ochre processing tools [61,91,92]. For a summary of previous research, see [61].

Evidence for ochre use at Porc-Epic Cave was first reported by Breuil and Wernert [105]. Clark and Williamson [95] recovered 214 ochre pieces and one limestone grindstone during the 1974 excavations. Ochre pieces and ochre-stained artefacts were also collected during the 1975–1976 excavations by Williamson but were not mentioned in the literature before their reassessment by us [92]. They include 4213 pieces (c. 40 kg) of red, brown and yellow iron-rich ochre pieces [61], as well as 21 ochre processing tools and two ochre-stained artefacts [91] found throughout the sequence, in different areas of the cave (S2 Supplementary Fig. 3).

S3 – Previous research: ochre processing tools

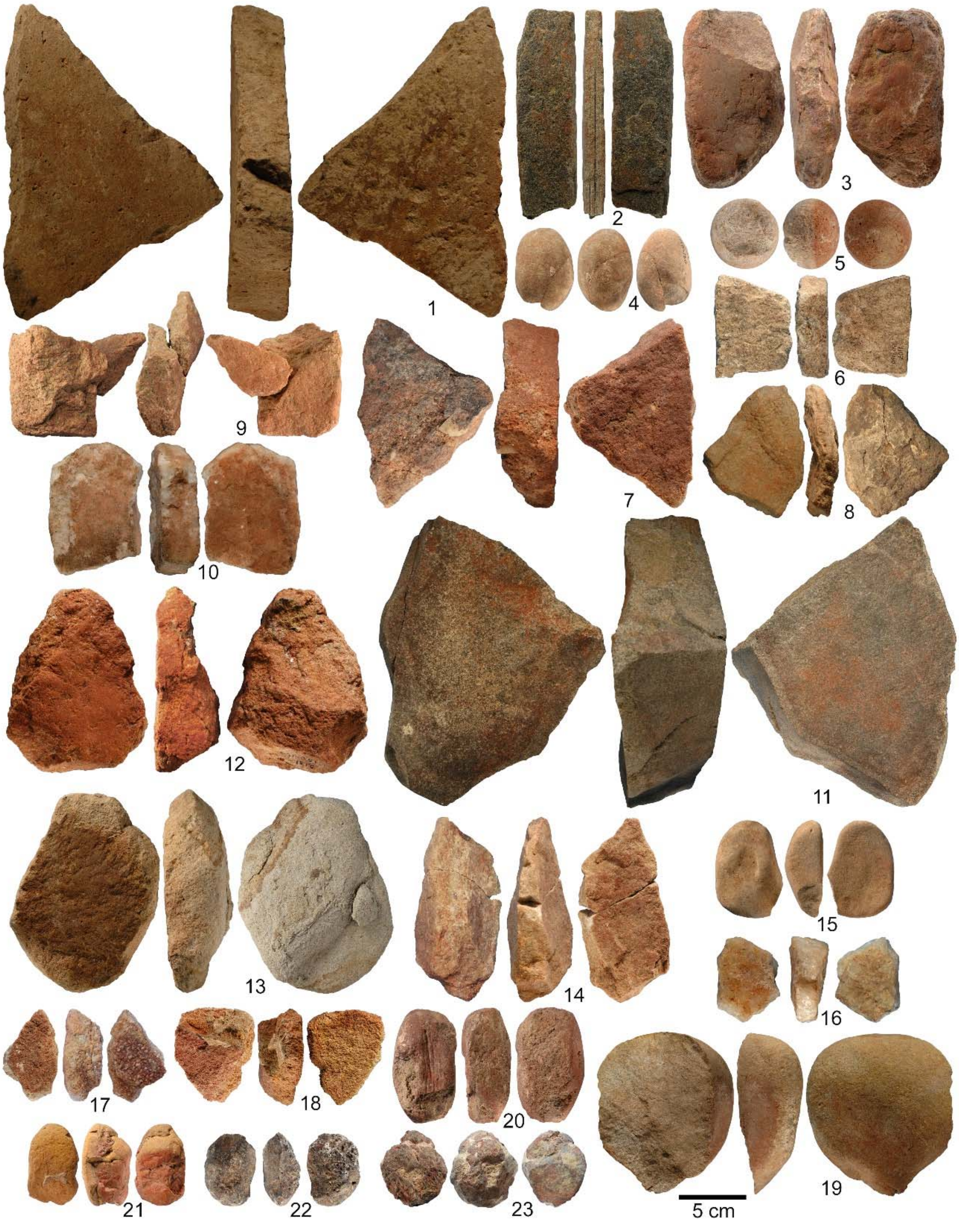

S3 Supplementary Figure 1. Ochre processing tools and ochre-stained artefacts found at Porc-Epic Cave.
Numbers correspond to the objects' identification number. Reprinted from [91] under a CC BY license, with permission from PLOS ONE, original copyright 2016.

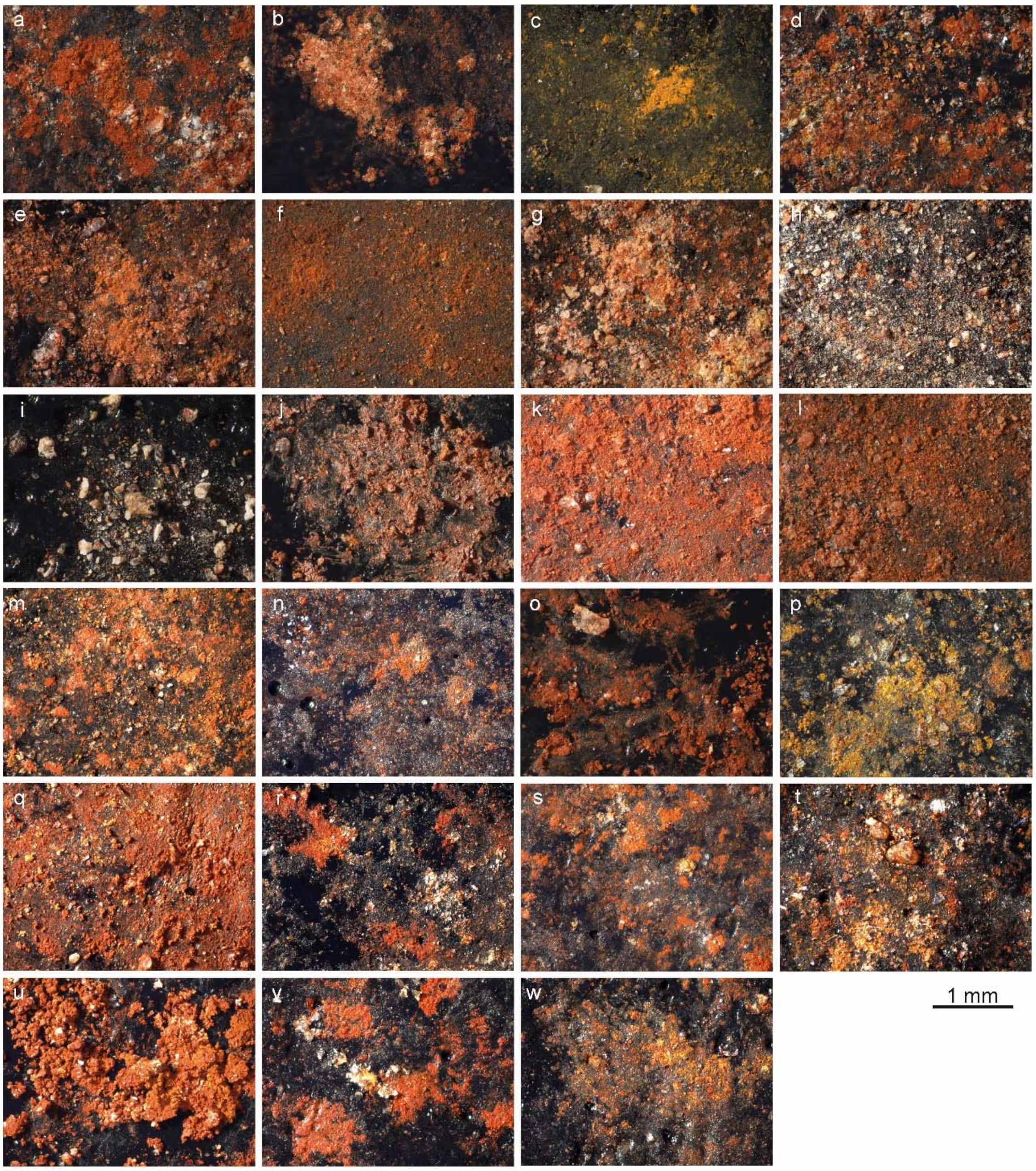

S3 Supplementary Figure 2. Ochre residues from ochre processing tools on carbon adhesive tabs. a: sample AT1A (OPT 1); b: sample AT1B (OPT 1); c: sample AT2A (OPT 2); d: sample AT2B (OPT 2); e: sample AT3 (OPT 3); f: sample AT4 (OPT 4); g: sample AT5 (OSA 5); h: sample AT6A (OPT 6); i: sample AT6B (OPT 6); j: sample AT7 (OPT 7); k: sample AT8 (OPT 8); l: sample AT9 (OPT 9); m: sample AT10 (OPT 10); n: sample AT11 (OPT 11); o: sample AT12 (OPT 12); p: sample AT13 (OPT 13); q: sample AT14 (OPT 14); r: sample AT15 (OSA 15); s: sample AT16 (OPT 16); t: sample AT17 (OPT 17); u: sample AT18 (OPT 18); v: sample AT19 (OPT 19); w: sample AT22 (OPT 22). Reprinted from [91] under a CC BY license, with permission from PLOS ONE, original copyright 2016.

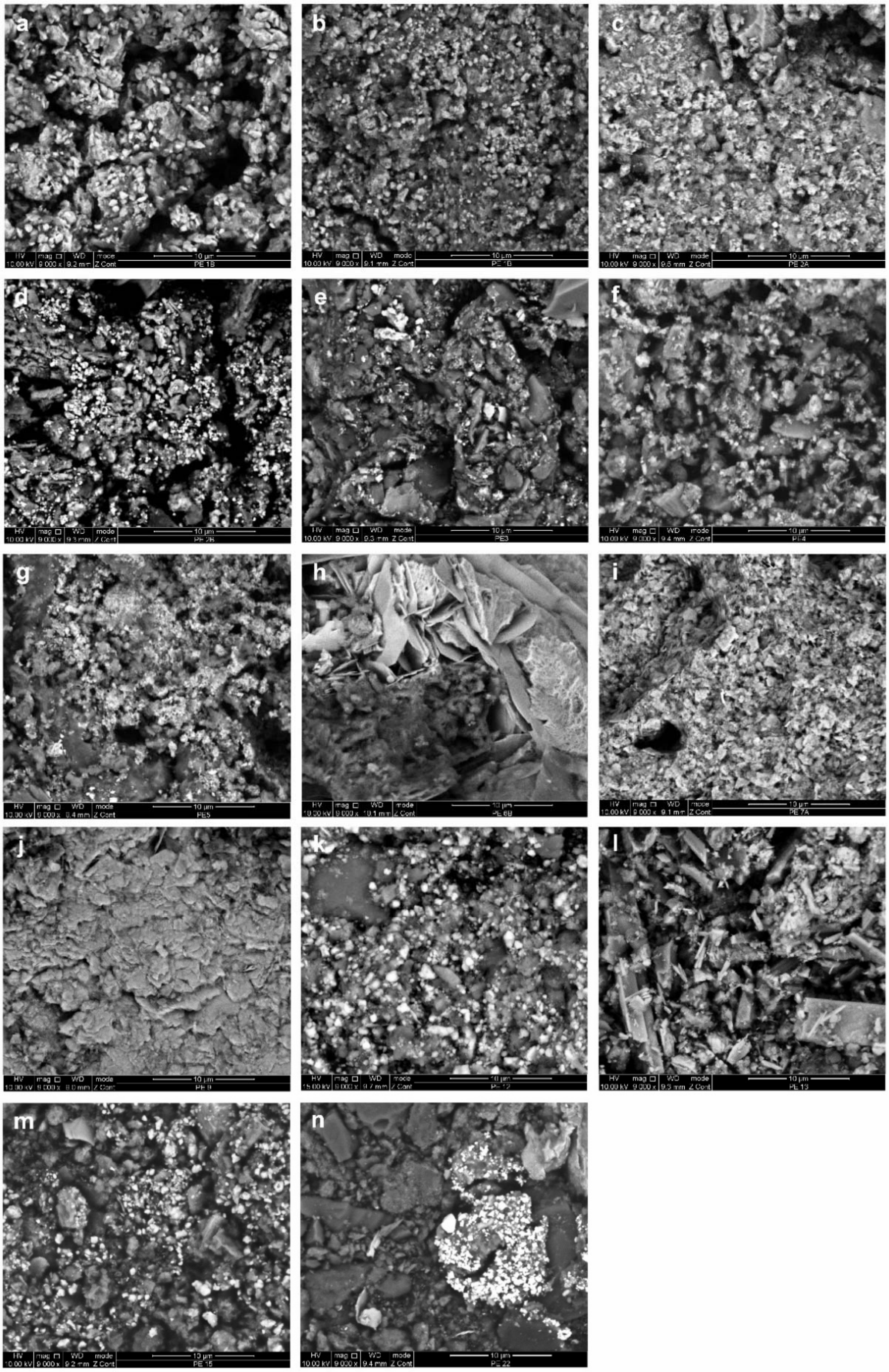

S3 Supplementary Figure 3. SEM images of ochre residues from Porc-Epic Cave's ochre processing tools and ochre-stained artefacts.

All figures are in backscattered electron (BSE) mode. a: OPT 1, sample AT1 zone 1; b: OPT 1, sample AT1 zone 2; c: OPT 2, sample AT2A; d: OPT2, sample AT2B; e: OPT 3, sample AT3; f: OPT 4, sample AT4; g: OSA 5, sample AT5; h: OPT 6, sample AT6; i: OPT 7, sample AT7; j: OPT 9, sample AT9; k: OPT 12, sample AT 12; l: OPT 13, sample AT13; m: OSA 15, sample AT15; n: OPT 22, sample AT22. Scales = 10 µm. Reprinted from [91] under a CC BY license, with permission from PLOS ONE, original copyright 2016.

S3 Supplementary Table 1. Results of SEM-EDS analyses on residues from Porc-Epic Cave's ochre processing tools and ochre-stained artefacts.

Num	Clay-sized particles (<4µm)		Silt and sand-sized particles (>4µm)	
	Shape	Composition*	Shape	Composition*
1	Aggl	Fe *(iron oxide)*	Irreg	Fe, Mn *(iron oxide)*
			Plat	Si, Al, K, Na *(K-rich mica)*
	Plat	Si, Al, Ca, K *(clay minerals)*	Ang	Ca, Mg *(carbonate)*
			Irreg	Ba, S *(Ba-rich sulphate)*
2	Aggl	Fe *(iron oxide)*	Irreg	Fe, Cr, Ni (*undet*)
			Ang	Si *(silicate)*
	Plat	Si, Al, Ca, K, Mg *(clay minerals)*	Irreg	Si, Al, Ca, K, Na *(Ca-rich feldspar)*
3	Aggl	Fe *(iron oxide)*	Irreg	Fe, Cr, Ni (*undet*)
			Subcirc	Fe *(iron oxide)*
	Plat	Si, Al, Ca *(clay minerals)*	Irreg/ang	Si *(silicate)*
4	Aggl	Fe, Si, Al, Ca, K, Mg, Na, Ti *(mixture of iron oxides and aluminosilicates)*	Oct	Fe, Ti *(undet oxide)*
	Plat	Si, Al, Ca *(clay minerals)*	Ang	Si *(silicate)*
5	Irreg/ acic	Fe *(iron oxide)*	Irreg	Fe *(iron oxide)*
			Irreg	Si, Al, Na, K *(feldspar)*
	Aggl	Si, Al, K, Ca *(clay mineral)*	Irreg	Si *(silicate)*
6	Aggl	Si, Al, Mg, K *(clay mineral)*	Irreg/plat	Fe, Ti *(undet oxide)*
			Ang	Si *(undet silicate)*
7	Aggl	Fe *(iron oxide)*	Irreg	Fe (*iron oxide*)
			Aggl	Si, Al, Ca, Na *(Ca, Na-rich feldspar)*
			Aggl	Si, Al, Ca *(undet aluminosilicate)*
			Irreg	Si *(silicate)*
9	Aggl	Fe *(iron oxide)*	Irreg	Si *(silicate)*
	Aggl	Si, Al, Ca, K, Na *(clay minerals)*	Irreg	Si, K, Al *(feldspar)*
12	Irreg	Fe *(iron oxide)*	Irreg	Fe, Si, Al, Mg, K, Cl, Ca (*undet*)
			Subcirc	Si, Ca, Al, K *(Ca-rich feldspar)*
	Plat	Si, Al, Ca, K, Mg *(clay minerals)*	Plat	Si, Al, K *(K-rich mica)*
13	Aggl	Fe, Si, Al, Ce, La, Nd, Na *(mixture of iron oxides and clay minerals)*	Tab	Si, Na, Al, K *(Na-rich feldspar)*
15	Aggl	Ba, S *(Ba-rich sulphate)*		
	Aggl	Si, Fe, Al, Ca, K, S (*mixture of iron oxides and clay minerals*)	Irreg	Fe, Ti *(undet oxide)*
22	Aggl	Fe, Si, Al *(mixture of iron oxides and aluminosilicates)*	Ang	Fe *(iron oxide)*
			Irreg	Fe, Si, Al *(Fe-rich silciate)*
			Irreg/ang	Si, Al *(undet aluminosilicate)*

Num: number; aggl: agglomerate; irreg: irregular; plat: platy; ang: angular; subcirc: subcircular; oct: octahedral; acic: acicular; tab: tabular. (*) Interpretation in brackets. The objects' identification number is the same as presented in S3 Supplementary Fig. 1. Reprinted from [91] under a CC BY license, with permission from PLOS ONE, original copyright 2016.

S3 Supplementary Table 2. Results of μ-RS analyses on residues from Porc-Epic Cave's ochre processing tools and ochre-stained artefacts.

Num	Sample	ab	an	aug	cal	dol	gth	gp	hem	kln	lep	mag	man	mnt	ms	qz	un. alum.	un. Mn ox.	C
1	AT1A,B						•		•						•		•		
2	AT2A,B						•		•						•	•	•	•	
3	AT3								•							•			
4	AT4						•	•	•							•	•		
5	AT5				•				•							•			
6	AT6A						•		•										
7	AT7						•		•		•					•			•
8	AT8						•	•	•							•			
9	AT9						•	•	•							•			
10	AT10				•		•		•				•			•	•		
11	AT11		•					•	•			•				•	•		
12	AT12	•							•							•	•		
13	AT13	•					•		•							•			•
14	AT14				•		•		•					•		•	•		•
15	AT15					•	•		•					•			•		
16	AT16						•	•	•							•			
17	AT17						•	•	•	•						•			
18	AT18						•	•	•			•				•			
19	AT19			•			•		•							•			
22	AT22						•		•					•		•			

Num: number; un: undetermined; alum: aluminosilicate; ox: oxide; C: carbon; abbreviations of minerals are based on the nomenclature suggested by [116] except for lepidocrocite (lep), and manganite (man). The objects' identification number is the same as presented in S3 Supplementary Fig. 1. Reprinted from [91] under a CC BY license, with permission from PLOS ONE, original copyright 2016.

S3 Supplementary Table 3. Results of XRD analyses on residues from Porc-Epic Cave's ochre processing tools and ochre-stained artefacts.

Num	Sample	ab	an	ant	aug	ber	cal	clc	gth	gp	hal	hem	kln	lmt	mc	mgh	mnt	or	qz	ram	sa	sap
1	T1		•				•					•	•						•			
3	T3					•						•	•						•			
5	T5									•		•		•			•		•	•	•	
6	T6	•	•		•			•			•	•							•			•
7	T7											•							•			
8	T8			•								•	•			•			•			
9	T9	•	?									•			?		•		•			
10	T10						•					•		•					•		•	
12	T12								•			•							•			
17	T17						•					•	•					?	•			
18	T18											•	•						•			

Num.: number; abbreviations of minerals are based on the nomenclature suggested by [116], except for bernalite (ber), halloysite (hal) and ramsdellite (ram). The objects' identification number is the same as presented in S3 Supplementary Fig. 1. Reprinted from [91] under a CC BY license, with permission from PLOS ONE, original copyright 2016.

S4 – Previous research: ochre pieces

Visual characterisation

Six types of raw material were identified macroscopically [61]:

Soft, fine-grained ferruginous rocks (SFG): homogeneous, fine-grained, clayish rocks with very few or no inclusions.

- Colour: mostly red and dark red, but also grey, brown, orange or yellow and only rarely bear small black spots.
- Morphology: most are slabs (n = 339) or irregular pieces (n = 218), although nodules (n = 117) and pebbles (n = 107) are also observed.
- Texture and density: several examples have a compact structure; others are laminated or show small cavities. Generally light.

Banded, fine-grained ferruginous rocks (BFG): rocks with the same texture and appearance as type SFG but with clearly differentiated layers of colours.

- Colour: mostly red and yellow, or red and orange but also dark red, grey or brown.
- Morphology: mostly found as nodules (n = 90), followed by smaller numbers of slabs (n = 23) and irregular pieces (n = 19) or pebbles (n = 14).
- Texture and density: generally light, these materials usually have a compact structure.

Hard, fine-grained ferruginous rocks (HFG): very hard and heavy iron oxides.

- Colour: dark colours, mostly grey and red, but also black. They rarely show brown, yellow, dark red, grey and orange spots.
- Morphology: usually occur as nodules (n = 51) and pebbles (n = 45) and more rarely as slabs (n = 33) and irregular pieces (n = 24).
- Texture and density: hard and heavy.

Coarse-grained ferruginous rocks (CG): heterogeneous agglomerates of grains of different colours and shapes.

- Colour: mostly red, dark red and grey grains, and more rarely yellow, orange, and brown grains.

- Morphology: generally irregular in shape (n = 49) or occur in the form of nodules (n = 42) and, to a lesser degree, pebbles (n = 25) or slabs (n = 28).

Ferruginous sandstone (FS): agglomerates of translucent grains (probably quartz) in a fine iron oxide matrix.

- Colour: mostly red and orange, sometimes with dark red, grey, brown and yellow spots.
- Morphology: commonly found as slabs (n = 5), nodules (n = 2) or pieces with irregular morphologies (n = 2).

Platy fine-grained ferruginous rocks (PFG): agglomerates of platelets characterised by a shiny or metallic-like appearance.

- Colour: usually greyish with red veins,
- Morphology: can be irregular (n = 2) or flat in shape (n = 1).

S4 Supplementary Table 1. Criteria for the determination of ochre types.

Raw material type	Colour	Texture		Inclusions	Hardness	Density
Soft fine - grained (SFG)	G, Y, BR, BL, O, R, DR	VF	Hom	None or few	Soft to hard	Light
Banded fine - grained (BFG)	Layers of Y, O, R, DR, G, BR	VF	Hom	None or few	Soft to hard	Light
Hard fine - grained (HFG)	G, Y, BL, O, R, DR, BR	VF	Hom	None	Very hard	Heavy
Coarse - grained (CG)	G, Y, BR, O, R, DR	C	Het	Subcirc / irreg	Soft to hard	Normal
Ferruginous sandstone (FS)	G, Y , BR, O, R, DR	C	Hom	Subcirc / irreg	Soft to hard	Normal
Platy fine - grained (PFG)	G, R	F + C	Het	Platelets	Soft	Light

G: grey; Y: yellow; BR: brown; BL: black; O: orange; R: red; DR: dark red; VF: very fine; C: coarse; F: fine; hom: homogeneous; het: heterogeneous; subcirc: subcircular; irreg: irregular. Reprinted from [61] under a CC BY license, with permission from PLOS ONE, original copyright 2017.

Anthropogenic modifications

Anthropogenic modifications, including traces of flaking, striations, facets, smoothed areas, incisions, and pits, were identified macro- and microscopically [61]:

- Pieces bearing traces of flaking include objects with simple or multiple flake scars and flakes.
- Striations (S4 Supplementary Fig. 1a, b) produced by grinding the piece against an abrasive surface are present as linear parallel marks arranged in groups [21,117,118]. Facets refer to areas flattened by grinding and covered with striations.

- Incisions (S4 Supplementary Fig. 1c, d) are present as sub-parallel, slightly curved marks displaying multiple grooves (or micro-striations defined as microscopically visible parallel striations) produced by the asperities of lithic cutting edges or other sharp tools during scraping or scoring [21,57,118].
- Smoothed areas (S4 Supplementary Fig. 1e) refer to homogeneous surfaces lacking irregularities and projections in comparison to neighbouring unmodified areas or those on which modification marks have been partially or fully erased [118].
- Percussion pits (S4 Supplementary Fig. 1f) take the form of depressions produced by a pounding action [91,119,120].

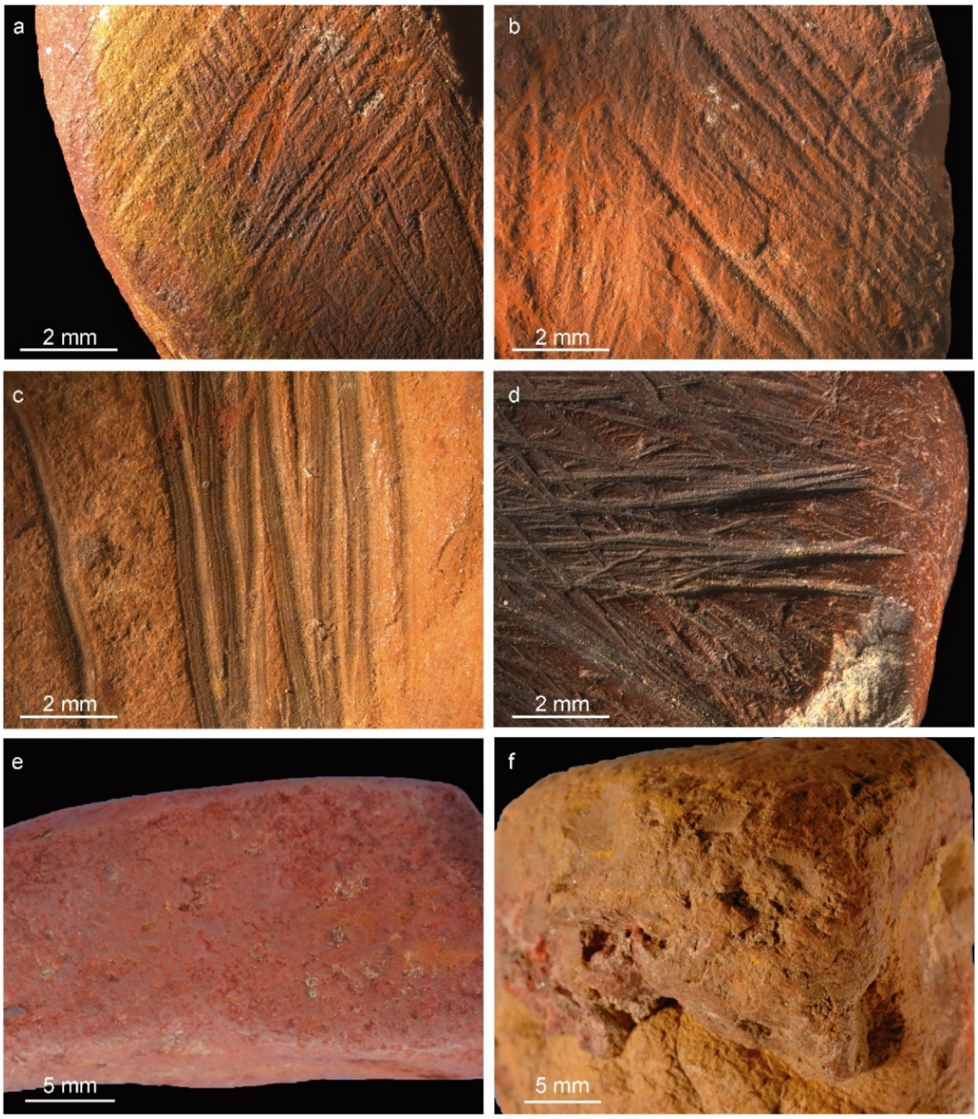

S4 Supplementary Figure 1. Modifications on ochre pieces.
a, b: Striations produced by grinding (ochre pieces PE102 and PE987); c, d: incisions produced by scraping/scoring (ochre pieces PE306 and PE1419); e: Smoothed areas (ochre piece PE3067); f: Pits produced by a pounding action (ochre piece PE931, OPT21). Reprinted from [61] under a CC BY license, with permission from PLOS ONE, original copyright 2017.

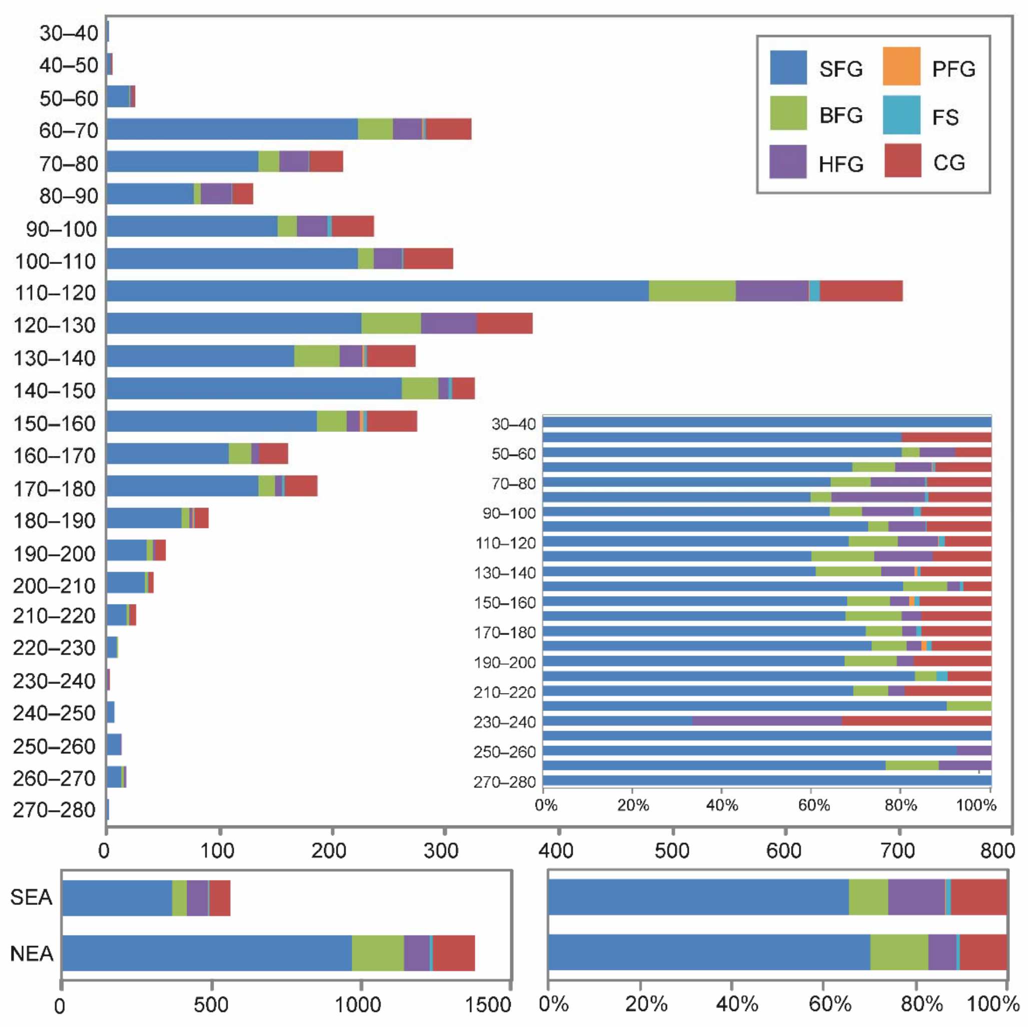

S4 Supplementary Figure 2. Vertical distribution of ochre pieces per raw material type.
Data are presented as number of pieces and percentages. Separate histograms are presented for ochre from the northeastern (NEA) and southeastern (SEA) accumulations. SFG: Soft fine-grained; BFG: banded fine-grained; HFG: hard fine-grained; PFG: platy fine-grained; FS: ferruginous sandstone; CG: coarse-grained. Reprinted from [61] under a CC BY license, with permission from PLOS ONE, original copyright 2017.

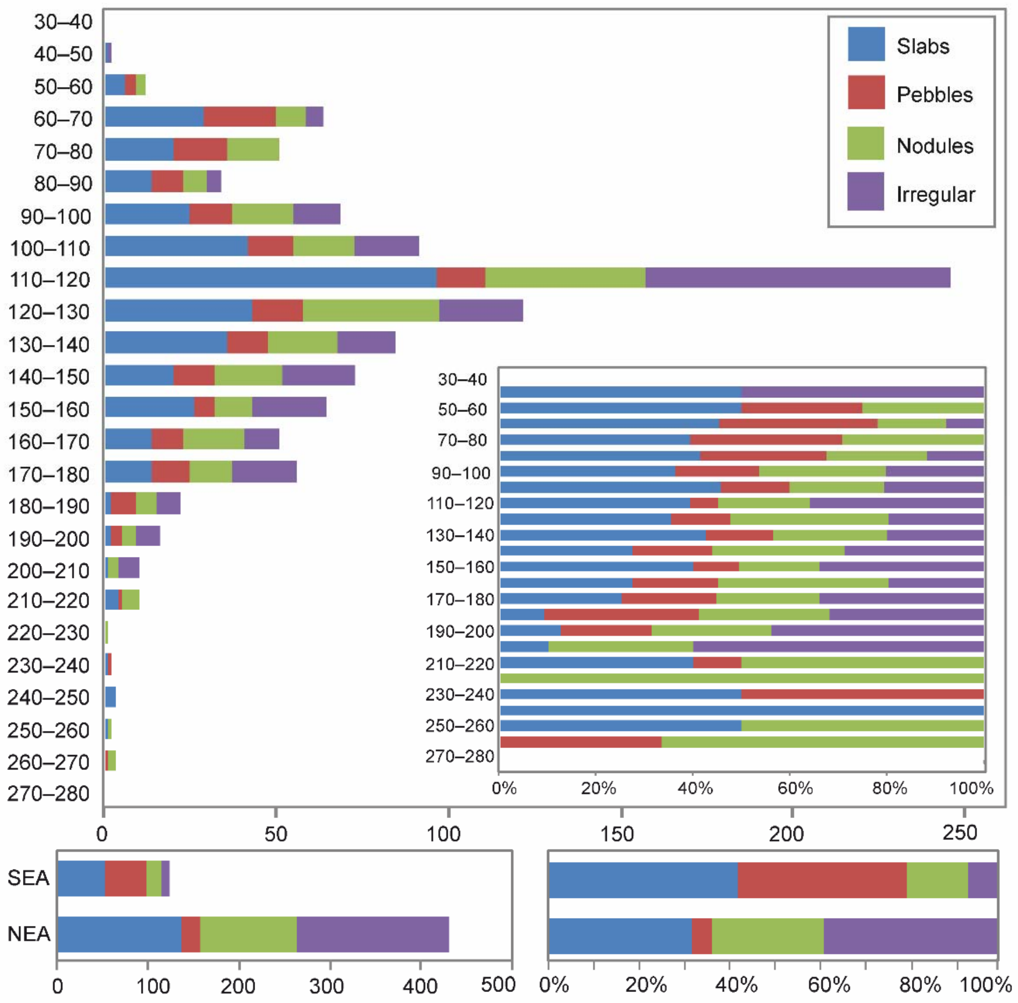

S4 Supplementary Figure 3. Vertical distribution of ochre piece morphology.
Data are presented as number of pieces and percentages. Pieces with undetermined morphologies were not included. Separate histograms are presented for ochre pieces from the northeastern (NEA) and southeastern (SEA) accumulations. Reprinted from [61] under a CC BY license, with permission from PLOS ONE, original copyright 2017.

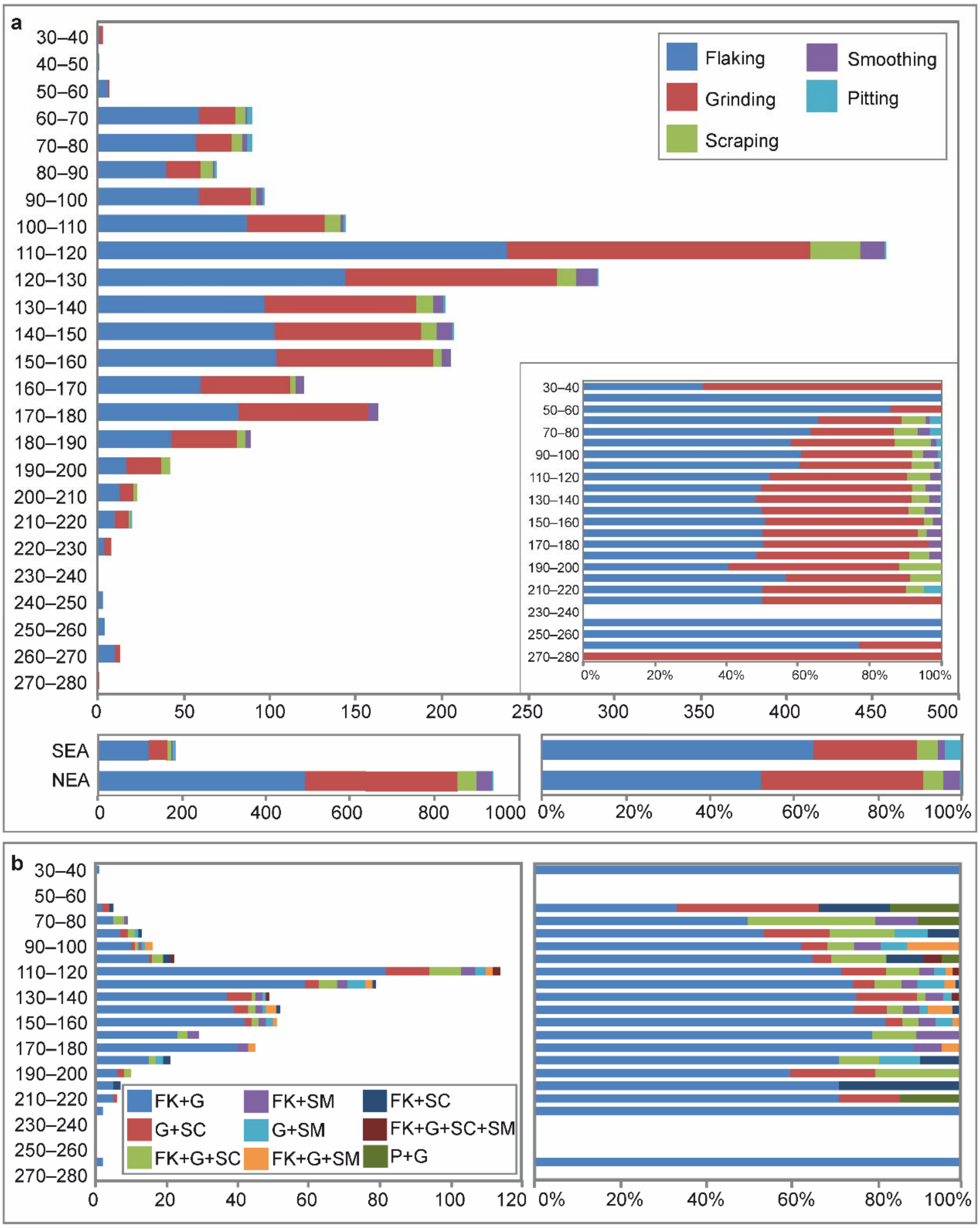

S4 Supplementary Figure 4. Vertical distribution of modifications identified on ochre pieces.
Data are presented as number of pieces and percentages; a: occurrence of each modification type throughout the sequence. Separate histograms are presented for ochre pieces from the northeastern (NEA) and southeastern (SEA) accumulations; b: occurrence of main combinations of modifications. Ochre pieces with only one modification or combinations that appear on less than 4 pieces were excluded. FK: flaking, G: grinding; SC: scraping; SM: smoothing; P: pitting. Reprinted from [61] under a CC BY license, with permission from PLOS ONE, original copyright 2017.

S5 – Ochre pieces: elemental data

S5 Supplementary Table 1. Results of XRF analyses conducted on ochre pieces from Porc-Epic Cave and natural pieces found in the surrounding area.

Ochre piece num	Raw mat	Analysis num	Si (%)	K (%)	Ca (%)	Ti (%)	Mn (%)	Fe (%)	V (ppm)	Cr (ppm)	Ga (ppm)	As (ppm)	Rb (ppm)	Sr (ppm)	Y (ppm)	Ba (ppm)
9	SFG	1	2,46	0,1605	0,4828	0,112	< 0,0041	85,21	559	3,7	9	88,4	64	65,5	64	93
9	SFG	2	3,17	0,1275	0,3136	0,0848	< 0,0038	74,94	392	< 45	13,3	66,7	56	54	56,6	< 77
9	SFG	3	3,61	0,1645	0,3816	0,1022	< 0,0039	78,39	443	< 46	13,8	68,3	48	56	75,6	< 80
9	SFG	4	4,31	0,187	0,4952	0,1459	0,0077	79,49	483	< 47	8,1	103,1	60	65,8	74,5	< 81
9	SFG	5	2,694	0,2098	0,5461	0,157	< 0,0043	83,01	498	< 48	13,1	105,6	45	63,4	82,6	< 83
17	SFG	1	11,6	2,147	3,219	0,2108	0,22	41,96	81	< 31	< 4,8	27,7	42	89,4	29,6	< 88
17	SFG	2	12,68	1,833	2,54	0,1856	0,2576	38,8	102	< 30	< 4,7	20,8	40	80,4	28,7	< 86
17	SFG	3	12,78	1,941	2,782	0,1945	0,1631	37,23	116	< 30	< 4,8	20,2	44	85,7	27,9	< 91
17	SFG	4	10,42	1,978	2,951	0,1993	0,2875	45,23	234	< 34	< 5,0	29,3	41	89,1	33,2	< 94
17	SFG	5	10,42	2,077	2,901	0,2002	0,3001	44,76	211	< 33	< 5,1	24	38	86,9	22,1	< 93
51	SFG	1	7,27	0,3209	0,9909	0,2143	< 0,0033	57,16	354	< 39	27,6	22,6	38	60,7	42,3	102
51	SFG	2	17,26	0,5599	2,526	0,4982	0,0994	31,64	77	148	25,5	13,5	43	187,5	478,1	< 90
51	SFG	3	12,19	0,7723	0,8894	2,237	0,111	37,59	3786	75	14,3	33,4	38	686,4	73	19730
51	SFG	4	13,83	0,3088	1,553	0,3141	0,01877	35,03	488	79,2	33,8	13,1	38	95,9	312,3	108,7
51	SFG	5	6,5	0,5234	0,8056	0,2526	0,0215	69,03	672	< 43	16,3	56,8	45	69,2	86,9	< 87
133	SFG	1	10,88	2,201	1,882	0,3841	0,7438	50,86	299	< 37	< 5,0	42,2	66,4	117,4	98,1	< 96
133	SFG	2	13,39	2,387	2,334	0,3879	1,117	39,49	293	41,9	< 4,8	32	61,9	145,9	104,9	< 92
133	SFG	3	11,76	2,009	2,539	0,3256	0,7012	39,68	221	41	< 4,7	26,5	48	124,8	97,4	< 91
133	SFG	4	13,02	2,567	1,807	0,4101	1,425	41,22	290	36	< 4,9	34,2	56,4	148,6	108,3	< 93
133	SFG	5	8,09	1,307	1,494	0,1903	0,4081	56,36	101	6,6	< 4,6	31,1	45	109,6	77,4	< 88
295	SFG	1	7,34	1,356	1,643	0,287	0,1433	55,19	241	< 38	< 4,5	30,7	50	61,4	40,3	< 81
295	SFG	2	9,64	1,098	1,3	0,2157	0,1754	33,61	284	6,9	< 3,4	15,1	38	58	19,1	< 1,0
295	SFG	3	7,74	1,395	1,424	0,3001	0,1377	58,22	222	< 39	< 4,7	28,3	46	54,6	29,6	< 84
295	SFG	4	8,37	1,313	1,746	0,2514	0,4901	55,9	567	8,2	< 4,7	31,6	38	65,7	28,3	< 87
295	SFG	5	8,07	1,381	1,307	0,2953	0,1132	55,87	189	< 38	< 4,5	22,8	50	63	34,1	< 83
381	SFG	1	< 2,2	0,2635	0,412	0,1027	0,3596	84,57	480	< 48	< 5,2	159,8	56	82,3	55,4	< 91
381	SFG	2	2,02	0,1935	0,3035	0,0847	0,2948	69,2	395	< 42	< 4,4	116,4	40	81,9	61,1	< 84
381	SFG	3	3,03	0,2572	0,3346	0,0879	0,2214	63,45	322	< 41	< 4,6	97,6	38	69,4	24,9	< 78
381	SFG	4	2,17	0,2462	0,4274	0,1166	0,35	81,98	431	< 48	< 5,3	163,6	72	80,4	42,1	< 89
381	SFG	5	3,16	0,2787	0,3454	0,116	0,2191	69,69	540	< 44	< 4,7	120,9	56	73,3	40,7	< 82
538	SFG	1	21,97	1,504	1,906	0,8208	< 0,00010	29,25	397	< 33	51,5	18,8	45	68,3	40,3	< 92
538	SFG	2	14,35	1,446	1,508	0,5663	< 0,0029	45,91	734	< 36	38	22,1	50	65,7	54,1	< 82
538	SFG	3	12,45	0,9265	1,307	0,611	< 0,0031	48,63	539	62,7	40,1	27,5	38	61,3	56,5	< 83
538	SFG	4	11,49	0,9299	2,266	0,2386	< 0,00010	52,29	596	< 37	46	31,3	38	65,7	311,5	< 88
538	SFG	5	8,98	1,138	1,608	0,482	< 0,0033	61,99	1022	< 41	32,3	37,8	38	55,9	31,1	< 86
610	SFG	1	6,18	0,8281	1,377	0,1737	1,812	62,03	705	13	< 5,0	77,2	38	113,5	45,1	< 88
610	SFG	2	5,02	0,8478	1,107	0,1987	2,053	66,29	622	1,5	< 5,1	95,8	62	116,4	57,1	< 92
610	SFG	3	5,65	0,77	0,9617	0,1719	1,019	51,68	496	< 38	< 4,3	53,4	38	91,6	50,6	< 82
610	SFG	4	5,49	0,8555	1,245	0,1859	1,042	65,64	362	< 42	< 4,7	57,6	69	94	66,4	< 91
610	SFG	5	6,19	0,7786	0,9953	0,1655	1,332	56,23	448	4,3	< 4,4	57,3	44	88,2	44,8	< 85
615	SFG	1	3,45	0,8138	2,192	0,1653	0,2575	66,35	412	< 40	< 4,8	87,9	39	73,7	34,7	< 80
615	SFG	2	4,44	0,8001	2,774	0,1739	0,1959	62,73	501	< 39	< 4,6	78,1	38	74,4	36,1	< 78
615	SFG	3	6,48	0,8706	1,827	0,1656	0,2445	45,81	247	< 33	< 4,0	40,1	38	64,3	16,7	< 66
615	SFG	4	5,81	0,913	2,687	0,1855	0,2527	53,46	386	< 36	< 4,3	52,2	38	75,7	32,4	< 75
615	SFG	5	4,96	1,136	1,973	0,1976	0,1974	56,82	224	< 37	< 4,4	56,8	40	75,1	35,4	< 79
710	SFG	1	3,59	0,2419	8,31	0,1287	0,0341	35,66	77	< 25	23,9	161	38	73,6	9,6	224
710	SFG	2	2,68	0,1549	3,727	0,03	0,0678	48,89	82	< 32	8,5	343,9	38	68,2	19,8	164
710	SFG	3	< 2,0	0,0965	2,225	0,021	< 0,0036	64,85	125	< 39	18,7	407,7	38	72,3	25	224
710	SFG	4	4,04	0,1638	5,28	0,021	0,103	49,84	98	< 32	8,3	344,9	38	74,7	12,3	< 84
710	SFG	5	2,42	0,118	2,773	0,0335	0,0871	59,22	94	< 37	< 7,2	490,8	38	66,9	21,6	175
725	SFG	1	8,14	2,002	3,969	0,3218	0,7662	59,58	708	< 39	< 5,1	105,5	52	107,8	68,8	< 93
725	SFG	2	10,38	2,133	3,596	0,3165	0,6439	47,85	569	< 35	< 5,1	58,6	40	101,5	52,7	< 85
725	SFG	3	8,39	2,145	3,671	0,3329	0,7832	53,48	517	11	< 4,7	81,4	55	112,7	66,8	< 91
725	SFG	4	6,43	1,804	4,545	0,3904	0,6422	56,95	888	< 37	< 4,7	71,6	51	108	51,7	< 87
725	SFG	5	6,53	1,451	2,795	0,2166	0,7221	56,38	460	< 37	< 4,6	48	38	104,4	74	< 89
726	SFG	1	12,08	1,486	2,162	6,497	0,1155	31,93	180	< 31	27,6	17	38	752,5	48,2	1242
726	SFG	2	9,94	1,244	2,063	6,352	0,1193	33,5	176	< 32	20,9	11,8	38	740,4	50,5	1556
726	SFG	3	8,8	1,374	2,087	6,407	0,1841	34,88	160	53	< 4,6	18,7	38	617,4	50,3	740
726	SFG	4	6,02	1,412	2,472	7,268	0,1706	40,58	< 63	< 35	< 5,1	31,7	38	653,9	61,8	912
726	SFG	5	8,53	1,25	2,264	6,72	0,1794	36,19	105	12	< 5,2	22,1	38	890,7	55,7	1991
737	SFG	1	17,71	0,7057	1,429	9,331	0,0294	27,8	< 65	< 27	34,5	10,5	38	63,9	< 2,1	< 86
737	SFG	2	18	0,4866	0,925	5,188	0,00575	27,53	307	< 27	30	14,8	38	64,4	< 1,0	< 78
737	SFG	3	15,48	0,6406	1,361	7,573	0,0409	27,15	< 60	< 27	35,2	11	38	70,7	6,3	96
737	SFG	4	24,51	0,5182	1,314	6,822	0,0331	25,79	204	< 26	34,5	14,7	38	73,3	4,2	< 81
737	SFG	5	15,28	0,85	1,98	10,64	0,0388	41,67	< 73	< 34	35,6	29,5	38	77,5	9,5	< 100
913	SFG	1	6,61	0,7934	2,457	0,2074	0,2586	62,82	312	< 40	< 5,0	47	40	70,6	99,3	< 1,0
913	SFG	2	5,9	0,763	2,452	0,1877	0,2729	64,33	250	< 40	< 4,6	45	55	79,2	93	< 87
913	SFG	3	5,7	0,7448	2,677	0,1746	0,2611	64,27	264	< 39	< 5,0	43	45	62,8	67,2	< 85
913	SFG	4	8,11	0,7778	2,731	0,2132	0,1725	58,28	323	< 38	< 4,7	37,1	47	74	110,2	< 87
913	SFG	5	9,7	0,9198	2,612	0,2381	0,1464	55,17	262	11	< 4,7	33	38	77	116	< 87
919	SFG	1	6,14	0,3995	1,368	0,1874	< 0,0035	64,29	560	< 41	32,4	47,5	38	53	66,4	< 85

919	SFG	2	6,18	0,4337	1,453	0,1955	< 0,0036	65,8	556	< 41	29	52,9	50	57,3	99,2	< 86
919	SFG	3	4,41	0,3108	1,198	0,1845	< 0,0037	71,28	603	< 43	22,3	50,4	49	46	283	< 87
919	SFG	4	4,94	0,4065	1,847	0,2076	< 0,0040	76,43	623	< 44	22,1	72,1	65	54	77,5	< 93
919	SFG	5	5,57	0,3839	1,354	0,1941	< 0,0037	69,11	530	< 43	26,8	51,5	39	49	325,8	< 88
930	SFG	1	8,07	1,152	3,519	0,338	0,1348	62,08	85	< 39	< 5,2	44,5	48	55,8	61,9	< 88
930	SFG	2	7,87	0,9866	3,13	0,2863	0,1382	58,74	111	< 38	< 4,8	45	53	61,2	64,4	< 86
930	SFG	3	11,64	1,385	2,469	0,3874	0,109	44,18	80	< 33	8,6	37,3	53	68	61,3	< 88
930	SFG	4	10,73	1,272	5,01	0,3587	0,1387	38,51	287	< 30	7,7	19,4	48	78,7	179,6	2,3
930	SFG	5	9,76	1,056	3,101	0,296	0,1185	52,68	108	< 36	< 4,6	28,5	38	54	57,4	< 82
987	SFG	1	5,27	0,713	1,007	0,1742	1,619	60,25	897	56	< 4,6	49,4	42	97,6	83,4	< 82
987	SFG	2	4,3	0,6727	1,122	0,1922	1,922	61,75	1049	52	< 4,6	58,1	49	112,2	103	< 84
987	SFG	3	4,81	0,6374	1,064	0,1531	1,772	66,4	1005	67	< 4,5	53,4	52	104,1	106,4	< 86
987	SFG	4	3,17	0,6419	1,016	0,1476	1,454	65,92	1015	< 43	< 4,8	55	38	86,7	84,1	< 85
994	SFG	1	22,78	0,5723	1,413	0,2722	0,02828	19,39	897	334	105,6	22,1	46,4	128,3	3681	< 80
994	SFG	2	30,98	0,2026	0,5381	0,2181	0,01625	11,45	667	297	122,9	10,9	38	98,6	1163	< 75
994	SFG	3	14,49	1,399	1,093	0,2453	0,0578	39,83	764	96,4	32,2	36,8	38	76,2	796,6	< 88
994	SFG	4	6,75	0,8063	0,7652	0,191	0,0893	55,3	746	77	6,8	40,2	38	73,4	1063	< 85
1087	SFG	1	10,44	0,8073	13,44	0,2189	0,4448	33,58	236	< 25	< 4,7	19,7	40	89,2	13,3	< 90
1087	SFG	2	9,82	0,8552	4,58	0,2859	0,6442	42,55	242	2,6	< 5,0	27,7	42	92	15,4	< 97
1087	SFG	3	11,36	0,6825	7,23	0,195	0,3791	26,33	253	< 24	< 4,3	13	44	85,9	25,4	< 84
1087	SFG	4	11,22	0,724	11	0,2001	0,3473	25,05	251	< 22	< 4,0	15,6	44,8	95,4	22,5	< 82
1087	SFG	5	10,75	0,8902	4,479	0,3148	0,6997	45,68	192	< 33	< 5,3	33,2	41	110,9	29,8	< 98
1277	SFG	1	7,83	0,7133	4,72	0,3098	0,3723	50,78	506	< 34	< 5,0	33,5	38	88,2	32,9	< 80
1277	SFG	2	7,43	0,7722	4,676	0,3071	0,3398	50,21	501	< 34	< 5,0	34,5	42	85,1	35,4	< 82
1277	SFG	3	9,67	0,7425	5,334	0,5325	0,3531	48,61	1277	< 34	< 5,1	35	38	109,7	20,7	< 81
1277	SFG	4	10,26	0,9656	3,167	0,5407	0,2671	48,35	854	< 35	< 5,3	50,8	47	86,4	16,6	< 81
1277	SFG	5	6,62	0,4487	4,004	0,252	0,286	50,15	726	< 34	< 5,1	45,2	38	72,5	18	< 78
1427	SFG	1	6,53	0,9809	9,648	0,1159	0,0844	28,27	240	56,5	27,1	7,7	39	251	214	117
1427	SFG	2	14,37	2,145	5,903	0,378	0,02221	21,99	200	26	47,7	8	48,2	306,7	280,9	114
1427	SFG	3	14,92	1,945	5,34	0,3899	0,00794	29,75	107	< 26	46,4	15,5	56	133,1	93,6	90
1427	SFG	4	13,02	1,55	5,189	0,3033	0,00972	26,87	117	< 25	42,8	13,6	50,7	119,2	79,2	< 87
1427	SFG	5	12,47	1,767	5,425	0,336	0,01901	33,28	140	< 27	43,4	22	53,2	125,7	115,5	< 90
1481	SFG	1	11,15	0,9269	1,773	0,3935	0,2691	46,37	325	< 35	< 5,2	111	64,6	73,3	56,7	< 93
1481	SFG	2	14	1,019	2,171	0,3966	0,2609	41,1	437	3,7	< 5,6	102,3	66	73,2	57,3	< 98
1481	SFG	3	15,32	0,8439	2,112	0,4976	0,2702	41,81	509	< 34	< 5,5	98	65,7	71,7	48,9	< 96
1481	SFG	4	13,19	0,7166	1,526	0,5899	0,1674	41,31	892	< 34	< 5,4	88,4	51	91,5	34,6	< 93
1481	SFG	5	10,81	1,019	2,018	0,3772	0,3256	49,35	452	< 36	< 5,9	125,6	62	60,5	49	< 97
1491	SFG	1	5,6	0,4915	1,202	0,2673	0,3326	62,97	400	42	< 4,8	41,1	38	77,3	64,9	< 87
1491	SFG	2	5,74	0,4745	1,272	0,2447	0,3079	62,26	272	52	< 4,5	40,2	38	79,8	94,3	< 89
1491	SFG	3	5,87	0,4179	1,085	0,3315	0,3414	60,45	703	50	< 4,6	34,3	45	84,1	108,4	< 89
1491	SFG	4	5,14	0,3969	1,501	0,2201	0,3425	64,95	489	48	< 4,6	53,1	52	82,3	135,1	< 87
1491	SFG	5	5,77	0,468	1,785	0,2304	0,3716	65,63	477	66	< 4,7	52,4	47	73,3	122,7	< 89
1493	SFG	1	5,59	0,8711	1,029	0,2966	0,8275	66,85	573	8,7	< 4,7	69,7	73	100,9	107,5	< 89
1493	SFG	2	5,03	0,7685	1,11	0,8678	0,7718	59,74	2283	< 43	< 4,9	51,3	46	165,8	69,3	< 85
1493	SFG	3	5,3	0,7789	1,928	0,4362	0,7566	64,08	1087	< 42	< 4,7	61,2	58	108,8	82	< 87
1493	SFG	4	5,85	0,8131	1,51	0,4663	0,7526	63,21	1123	< 42	< 4,7	55,9	58	112,3	109,3	< 85
1493	SFG	5	4,88	0,7736	1,071	0,252	0,7477	62,84	466	< 41	< 4,7	46,9	51	78,4	73,4	< 84
1526	SFG	1	7,51	0,6167	1,038	0,285	< 0,0034	66,24	312	12	27,8	39,8	38	52	81,4	< 86
1526	SFG	2	14,66	0,5866	1,293	0,3908	< 0,0026	37,3	77	79,6	40,6	15,5	46	122,6	104,2	557
1526	SFG	3	24,53	1,581	1,216	0,6284	< 0,0023	25,06	366	44,1	60	13,7	75,4	86,2	30,7	< 84
1526	SFG	4	8,6	0,5625	0,9457	0,2224	< 0,0031	56,02	304	< 38	30,1	28,5	38	50	21,9	83
1526	SFG	5	25,77	1,651	1,098	0,7532	< 0,0020	18,93	237	63,5	62,6	14,4	101,6	96,4	45,9	< 81
1552	SFG	1	5,8	0,5243	0,7576	0,3948	0,582	58,34	853	< 41	< 4,4	55,1	38	80	40,2	< 76
1552	SFG	2	7,46	0,614	0,8733	0,3937	0,4507	55,86	926	< 40	< 4,3	59	42	78,5	45,4	< 76
1552	SFG	3	6,78	0,7023	1,059	0,5941	0,5908	57,73	1628	< 41	< 4,8	49,8	44	73,2	19,3	< 81
1552	SFG	4	5,09	0,5923	0,8276	0,2236	1,152	68,64	655	< 44	< 4,7	67,9	62	75,2	55	< 84
1552	SFG	5	4,25	0,5518	0,7242	0,2716	0,4075	66,57	619	< 43	< 4,5	54,8	48	78,3	46,2	< 83
1566	SFG	1	16,94	0,9594	1,054	0,3222	0,0763	37,62	134	< 31	18,4	16,8	51	68,6	35	< 90
1566	SFG	2	19,92	0,98	1,127	0,3555	0,0913	32,11	151	< 29	20,5	18,1	51,5	82	43,5	< 93
1566	SFG	3	28,29	1,818	0,9106	0,4969	0,03401	11,19	171	< 18	50,9	3,9	79,1	84,5	27,4	< 98
1566	SFG	4	15,7	0,9049	1,067	0,2997	0,0772	41,22	141	1,5	18,2	23,7	48	75,7	53,4	< 87
1566	SFG	5	18,96	0,8515	1,118	0,3011	0,1213	35,11	118	< 31	9,9	13,7	42	65,4	26,1	< 96
1626	SFG	1	9,53	0,9338	1,606	0,3136	0,4042	56,26	173	7,5	< 4,4	34	40	96,4	45,2	< 89
1626	SFG	2	8,39	0,8784	1,629	0,2976	0,4465	58,04	212	< 39	< 4,5	41,7	52	90,3	43,5	< 90
1626	SFG	3	9,35	1,16	1,812	0,3596	0,2725	58,85	206	< 39	< 4,9	35,6	50	83,5	33	< 93
1626	SFG	4	9,45	1,158	2,067	0,3414	0,5729	58,23	195	< 39	< 4,8	37,6	56	101,1	41,8	< 95
1626	SFG	5	8,15	0,8587	1,498	0,2607	0,4372	54,38	194	< 38	< 4,4	33,1	44	85,3	35,2	< 86
1635	SFG	1	3,43	0,2399	3,114	0,0659	1,474	76,01	78	< 42	< 4,8	61,2	39	74,3	67,9	< 84
1635	SFG	2	2,66	0,2208	3,384	0,1363	2,137	76,31	309	< 42	< 4,8	67,8	43	107	95,7	< 81
1635	SFG	3	2,3	0,1871	3,253	0,1381	2,152	76,66	382	< 43	< 4,9	64,3	38	105,3	80,7	< 81
1635	SFG	4	3,21	0,2531	2,377	0,0797	1,463	76,91	112	< 43	< 4,9	61,2	65	91,7	73,2	< 89
1635	SFG	5	3,9	0,2667	2,571	0,0914	1,746	69,76	135	< 41	< 4,5	53,5	38	93,5	68,6	< 78
1637	SFG	1	7,18	1,045	1,916	0,3839	0,393	59,03	799	3,3	< 5,2	59,6	52	87,7	28,7	< 96
1637	SFG	2	6,94	0,9281	1,474	0,2862	0,4713	56,26	601	< 39	< 4,9	69,3	50	80,8	63,2	< 93

1637	SFG	3	7,22	0,9354	1,454	0,2758	0,4737	52,99	581	< 38	< 4,8	58,1	55	77,7	48,8	< 90
1637	SFG	4	6,96	1,028	1,919	0,4186	0,4039	58,8	869	6	< 5,1	54,6	51	90,5	27,5	< 97
1637	SFG	5	5,65	0,8944	1,376	0,2784	0,4736	54,48	578	< 38	< 4,9	59,7	53	76,6	48,4	< 92
1677	SFG	1	28,38	0,601	1,372	0,9734	< 0,0018	13,61	382	159	105,9	22,5	36,7	503,5	43,9	320
1677	SFG	2	28,21	0,6829	1,551	1,011	< 0,00010	16,82	377	59,6	94,4	26	38,3	529	35,6	153
1677	SFG	3	25,2	0,8524	1,647	0,833	< 0,0020	18,65	403	80,5	99,8	28,1	41,3	500,6	45,8	233
1677	SFG	4	30,92	0,6725	1,762	1,003	0,00241	10,06	381	120	117,3	12,4	25,9	536,7	50,7	296
1677	SFG	5	27,07	0,6655	1,523	1,036	< 0,0020	18,63	384	77,9	97,2	27	37,6	573,9	34,3	171
1699	SFG	1	6,99	1,47	1,755	0,2655	0,9099	58,04	491	< 39	< 5,2	127,8	45	87	34,8	< 84
1699	SFG	2	7,9	1,468	1,63	0,2312	0,6853	50,63	439	< 37	< 4,7	100,8	40	98	54,9	< 84
1699	SFG	3	8,13	1,73	1,739	0,7693	0,5223	52,56	1861	5,8	< 5,3	107,3	48	150,4	27,6	< 87
1699	SFG	4	7,56	1,425	1,48	0,2377	0,5964	51,79	373	< 37	< 4,9	101	38	97,5	62,3	< 84
1699	SFG	5	12,66	2,077	1,598	0,3182	0,1265	38,43	165	< 31	6,1	27,1	45	81,2	41,4	< 74
1737	SFG	1	8,9	1,967	1,684	0,26	0,1264	61,96	372	< 40	< 4,5	54,7	44	71,1	41,2	< 83
1737	SFG	2	10,79	1,932	1,626	0,2552	0,1472	54,92	256	9,1	< 4,2	48,4	59	66,3	43,6	< 78
1737	SFG	3	7,52	1,228	1,832	0,1773	0,7569	62,01	182	< 39	< 4,6	39,2	44	61,7	17,7	< 80
1737	SFG	4	8,96	1,778	1,595	0,2527	0,7164	60,1	343	5,1	< 4,8	56,3	57	60,6	20,8	< 81
1737	SFG	5	9,78	1,866	1,625	0,281	0,5238	58,06	353	5,4	< 4,7	46,5	46	62,3	22,8	< 79
1780	SFG	1	5,38	1,363	3,569	0,1677	0,8036	59,07	140	< 37	< 4,5	41,5	38	84,8	41,8	< 87
1780	SFG	2	5,24	1,429	2,871	0,1749	0,9335	60,28	78	< 38	< 4,5	46,4	42	86,7	43,7	< 86
1780	SFG	3	5,42	1,465	2,878	0,1552	0,9385	60,03	77	< 38	< 4,7	37	43	83,9	27,3	< 87
1780	SFG	4	4,24	1,377	2,288	0,1741	0,5749	60,74	109	< 39	< 4,6	41,8	42	83,7	27,6	< 86
1780	SFG	5	5,33	1,45	2,988	0,1639	0,9342	60,37	112	< 38	< 4,5	45,7	38	89,8	39,8	< 85
1845	SFG	1	5,81	0,6912	2,915	0,1295	0,4636	55,27	924	< 38	< 5,1	56,6	38	79,2	75,9	< 82
1845	SFG	2	5,82	0,7183	3,198	0,124	0,6434	51,21	880	< 1,0	< 4,9	52,3	38	88,3	78,2	< 81
1845	SFG	3	6,65	0,6915	3,459	0,2422	0,518	58,05	1360	44	< 4,9	65,6	48	91,5	80,8	< 84
1845	SFG	4	7,41	0,7731	5,652	0,1578	0,8236	57,28	1131	5,2	< 5,0	54,2	38	97,8	117,2	< 87
1845	SFG	5	6,84	0,7222	3,014	0,1372	0,498	55,32	888	1,4	< 4,8	59,5	51	97,1	106,1	< 86
1862	SFG	1	9,71	1,978	0,5841	0,3994	0,0413	47,25	294	6,9	20,6	22,6	64,2	60,1	77	< 85
1862	SFG	2	7,51	1,687	0,6858	0,3178	0,0332	56,74	241	5,4	17	34,1	60	46	37,7	< 88
1862	SFG	3	10,28	1,572	0,9751	0,2696	0,0184	55,28	336	54,2	25,9	26,2	52	55,6	147,3	< 84
1862	SFG	4	12	2,163	1,279	0,7935	0,0575	42,96	1638	42	20,6	24,5	57,5	72,6	26,4	129,2
1862	SFG	5	7,74	1,697	0,7184	0,3133	0,0322	57,66	230	< 39	15,8	31,6	55	50	41	131
1914	SFG	1	8,76	0,8518	2,675	0,2904	0,2172	56,19	202	< 37	< 4,6	49,6	70	71,1	71	< 86
1914	SFG	2	9,93	0,8557	2,579	0,306	0,3328	49,95	232	< 35	< 4,5	43,6	45	76,8	66,2	< 87
1914	SFG	3	12,35	1,091	2,792	0,3496	0,2072	45,44	216	< 34	< 4,8	34,9	39	68,7	57,2	< 92
1914	SFG	4	8,57	1,019	2,66	0,3108	0,5332	49,86	513	< 35	< 4,7	56,7	60,8	72,2	68,9	< 84
1914	SFG	5	22,27	2,387	3,194	0,4559	0,1046	16,81	148	< 21	25	13,7	87,9	63,5	35,6	< 87
1927	SFG	1	4,78	0,5853	1,292	0,1881	0,1618	66,89	534	< 42	< 4,9	127,3	50	76,6	58,4	< 86
1927	SFG	2	4,04	0,5686	1,908	0,1762	0,2486	67,87	572	< 42	< 5,1	153,6	54	75,1	56,7	< 85
1927	SFG	3	5,6	0,6786	2,187	0,363	0,1633	62,12	1058	3,3	< 5,1	112,9	48	151,9	23,2	191,4
1927	SFG	4	4,66	0,7507	2,35	1,7	0,1916	49,62	4081	50	< 5,2	79,6	38	1214	11	28210
1927	SFG	5	4,21	0,5218	1,188	0,1726	0,2217	68,28	564	< 43	< 4,9	168,3	47	85,8	72,5	< 87
1942	SFG	1	4,68	0,3585	1,292	0,147	0,1206	56,04	363	< 38	< 4,3	95,3	38	78,2	59,3	82
1942	SFG	2	4,76	0,4728	1,663	0,17	0,0977	56,05	381	< 38	< 4,3	86,8	38	65,4	31	115
1942	SFG	3	6,39	0,5861	2,779	0,1565	0,0452	31,83	183	< 27	17,7	36,4	38	63,2	18,8	183
1942	SFG	4	6,02	0,8211	1,345	0,1958	0,0679	61,9	301	13	6,2	100,1	48	70,4	35,8	102
1942	SFG	5	5,04	0,4496	1,554	0,1651	0,1291	64,6	416	< 41	< 4,7	123,2	39	80,5	40,4	< 85
1981	SFG	1	7,46	0,3782	5,47	0,2146	0,1634	38,28	347	< 1,0	< 5,0	33,1	38	67,7	12,6	82
1981	SFG	2	5,94	0,3206	4,077	0,1497	0,1222	39,47	347	< 30	9,7	31,1	38	66,5	11,8	92
1981	SFG	3	8,13	0,4962	8,077	0,2358	0,3154	44,06	430	< 30	< 5,5	34,1	38	106,8	30,7	< 88
1981	SFG	4	11,86	0,7368	10,08	0,3375	0,3448	28,78	428	< 24	< 5,6	20	43	112,4	13,4	< 82
1981	SFG	5	13,21	0,5711	5,519	0,3641	0,2386	16,55	576	6,1	< 4,7	12,1	44,1	86,5	< 2,0	< 67
2023	SFG	1	4,02	0,4991	0,9541	0,1412	0,0954	62,29	177	< 40	< 4,6	34,7	59	113,5	235,4	< 90
2023	SFG	2	6,36	0,6892	1,105	0,2013	0,0717	60,19	290	5,9	5,6	42,3	47	65,2	59,1	121
2023	SFG	3	3,61	0,6669	1,094	0,1897	0,2556	75,46	387	11	< 5,0	107,6	86	76,9	82,8	< 90
2023	SFG	4	5,23	0,7301	1,091	0,2844	0,1399	63,18	819	14	< 4,7	49,4	50	70,4	48,3	< 85
2023	SFG	5	5,43	0,8304	1,208	0,2298	0,1491	63,34	549	< 41	< 4,7	55,8	54	77,6	61,8	< 85
2063	SFG	1	6,32	1,142	5,12	0,2469	1,087	58,77	560	< 37	< 4,4	38,9	38	100,6	47	< 85
2063	SFG	2	6,05	1,143	6,163	0,2525	1,259	59,38	545	< 37	< 4,5	37,8	52	90,9	49,2	< 80
2063	SFG	3	5,48	1,005	1,498	0,1596	0,4838	62,91	359	< 40	< 4,7	43,7	51	78,4	56	< 88
2063	SFG	4	3,94	0,5066	1,011	0,1148	0,4948	63,38	363	1,4	< 4,6	53	49	105,4	124,4	< 90
2063	SFG	5	8,6	1,17	4,398	2,094	0,8326	44,26	5500	< 1,0	< 4,6	31	39	282	38,3	< 81
2067	SFG	1	5,31	0,7719	1,766	0,1681	0,1169	66,64	106	3,3	< 4,5	45,2	44	63,3	39,3	< 82
2067	SFG	2	5,12	0,7906	1,79	0,1696	0,1196	68,9	145	< 42	< 4,6	52	40	65	34,2	< 84
2067	SFG	3	5,17	0,6404	4,371	0,184	0,3654	72	354	66	< 5,0	54,3	40	84,3	124,1	< 91
2067	SFG	4	4,71	0,7098	2,507	0,1644	0,1293	69,18	145	< 41	< 4,5	53,7	42	66,7	55,9	< 85
2067	SFG	5	7,49	0,683	2,95	0,1756	0,285	54,47	265	< 36	< 4,2	36,1	38	74,2	55,1	< 76
2069	SFG	1	4,86	0,6702	3,242	0,1962	0,3056	65,34	1166	15	< 4,7	76,3	40	79,8	85,1	< 85
2069	SFG	2	4,77	0,6124	4,69	0,1863	0,1814	67,29	1176	97	< 4,9	46,4	38	67,2	104,7	< 88
2069	SFG	3	5,5	0,6373	3,084	0,2054	0,2839	65,01	1277	89	< 4,8	66,3	38	77,9	76,7	< 1,0
2069	SFG	4	6,25	1,142	2,684	0,1757	0,4189	59,75	1001	< 39	< 4,9	59	38	74,7	55,9	< 84
2069	SFG	5	6,75	0,9893	2,433	0,1782	0,3461	55,19	963	2,4	< 4,6	39,4	45	68,2	59,3	< 81
2091	SFG	1	13,76	0,6848	6,604	0,1358	0,3078	38,62	191	< 29	< 5,3	23,9	38	106,1	80,4	< 76

2091	SFG	2	11,68	0,6897	6,181	0,1582	0,2915	38,99	287	2,9	< 5,4	31	38	115,4	103,2	< 78
2091	SFG	3	10,13	0,6352	4,888	0,2246	0,1804	52,44	350	< 35	< 5,4	34	38	107,1	109,6	< 80
2091	SFG	4	12,84	0,6802	6,333	0,1366	0,2948	39,54	253	7,6	< 5,4	23,4	38	105	87,7	< 77
2091	SFG	5	10,23	0,6471	5,076	0,2193	0,1915	51	375	56,1	< 5,5	41,4	38	106	117,4	< 81
2159	SFG	1	4,2	0,422	0,4635	0,2453	< 0,0039	79,13	516	51	14,6	81	41	61	27,6	< 85
2159	SFG	2	5,97	0,5947	1,373	0,183	< 0,0036	71,41	175	< 43	23,3	77,5	45	93,3	117,6	< 94
2159	SFG	3	3,82	0,4818	0,8782	0,135	< 0,0038	78,02	140	< 45	14,3	72,7	64	53	47,2	< 90
2159	SFG	4	3,81	0,4417	0,7992	0,1271	< 0,0038	79,62	181	< 46	16,3	76	43	61	39,1	2,2
2159	SFG	5	7,86	0,6374	1,12	0,1901	< 0,0031	52,7	179	< 37	24,6	49,5	38	94,4	103,2	105
306	BFG	1	8,9	0,9853	1,522	0,3697	0,0536	51,68	586	< 37	18,7	61,4	44	60,2	50	94
306	BFG	2	18,49	1,778	1,937	0,5691	0,0705	29,48	625	115	30,4	43,3	72,2	63,3	36,7	< 87
306	BFG	3	19,33	1,922	2,105	0,6062	0,0908	29,18	620	101	23,9	55,6	83,2	70,3	42,6	< 89
306	BFG	4	19,96	2,038	2,123	0,6459	0,0808	28,5	633	126	27,8	55,2	80,5	59,6	25,1	< 89
306	BFG	5	11,21	1,101	1,492	0,4062	0,0418	47,62	617	53	23,1	58,8	54	55,5	42,8	< 89
521	BFG	1	9,14	0,605	1,513	0,1504	0,2019	51,36	86	< 36	< 4,6	32,1	40	87,7	82,1	< 92
521	BFG	2	8,45	0,5738	1,5	0,1573	0,1691	52,23	77	< 36	< 4,6	32,2	38	88,1	80,5	< 91
521	BFG	3	5,76	0,5561	1,527	0,2121	0,6532	58,37	732	< 40	< 4,6	62,5	38	112,1	79,3	< 87
521	BFG	4	6,13	0,5485	1,449	0,278	0,7023	57,93	921	4,9	< 4,7	70,9	40	125	76,6	< 88
901	BFG	1	9,77	1,149	2,27	0,2983	0,1218	44,67	296	45	5,5	68,9	38	1692	89,5	740
901	BFG	2	9,37	1,313	1,381	0,2051	0,1091	40,2	159	< 31	6,6	54,6	57,7	312,3	91,4	105
901	BFG	3	11,6	2,009	1,68	0,2514	0,0706	33,68	190	< 29	18,5	38,7	39,4	758,2	112,6	307
901	BFG	4	12,46	1,449	2,395	0,234	0,163	43,69	87	< 33	< 4,5	46,9	49	103,5	75,3	< 75
901	BFG	5	11,47	1,88	1,815	0,2326	0,1255	39,39	149	< 31	< 4,9	58,5	66,7	342,9	91,5	87
1499	BFG	1	11,03	0,8918	0,5744	0,2685	0,00402	44,62	104	< 34	28,6	13,6	39	66,5	45,8	< 87
1499	BFG	2	11,09	0,746	0,5601	0,2044	0,0191	46,53	79	< 35	25,9	23,8	39	72,2	78,5	< 86
1499	BFG	3	10,23	0,7458	1,169	0,2505	0,5006	47,38	454	48	< 4,4	41,8	45	94,3	128,5	< 87
1499	BFG	4	10,72	0,8709	1,702	0,2595	0,4953	52,02	413	< 37	< 4,8	47,4	52	84,5	70,7	< 88
1499	BFG	5	13,42	1,082	1,706	0,277	0,4342	44,39	387	< 34	< 4,9	42,5	52	91,3	90,1	< 92
1700	BFG	1	6,16	0,8086	1,413	0,3061	0,2901	64,3	552	14	< 5,0	71,1	39	79,2	56,9	< 90
1700	BFG	2	7,93	0,7181	1,203	0,2759	0,2233	52,77	470	< 38	< 4,6	48,8	38	65,8	38,9	< 81
1700	BFG	3	8,36	1,074	1,538	0,2334	0,1134	57,75	215	< 39	< 4,7	40,3	39	65,6	42,7	< 88
1700	BFG	4	10,65	1,335	1,27	0,2797	0,5776	47,57	109	< 36	< 4,9	20,5	52	70,7	36,5	< 94
1700	BFG	5	6,25	0,809	1,406	0,3166	0,293	65,72	577	< 42	< 4,9	67,7	40	78,1	61,5	< 90
1806	BFG	1	23,62	1,525	3,956	0,7734	0,2572	14,96	252	1,8	< 5,3	18,7	62,5	87,9	32	< 84
1806	BFG	2	9,25	0,8199	3,405	0,2092	0,5129	30,65	252	< 27	< 5,8	30,5	48	92,8	13,2	< 85
1806	BFG	3	9,99	0,6807	3,667	0,1632	0,4405	26,38	227	< 25	< 5,5	23,3	50,5	96,4	11,9	< 88
1806	BFG	4	10,07	0,6412	3,033	0,1667	0,5	34,99	295	< 29	< 5,6	36,6	40	84,8	17,5	< 84
1806	BFG	5	4,26	0,1839	1,128	0,1238	0,2579	51,05	296	< 37	< 4,9	34,3	38	103,6	43,9	< 90
2104	BFG	1	6,96	0,7253	1,499	0,1732	0,2368	53,6	275	84	< 4,4	25,7	38	88,8	157,5	< 1,0
2104	BFG	2	7,95	0,8236	1,483	0,1713	0,2748	57,6	183	< 39	< 4,8	30,2	38	59,2	65,1	< 86
2104	BFG	3	6,29	0,7093	1,235	0,1913	0,3188	58,09	249	< 39	< 4,4	36,3	45	69,6	77,8	< 86
2104	BFG	4	5,33	0,8655	1,51	0,2018	0,365	65,73	307	< 42	< 5,0	43,7	54	77,5	122	< 94
2104	BFG	5	7,72	0,7974	1,368	0,1705	0,3157	56,61	175	2,2	< 4,7	32	39	62,6	72,7	< 87
102	HFG	1	6,21	0,8705	1,073	0,2098	1,023	62,73	594	< 41	< 5,0	61,7	54	71,2	41,4	< 87
102	HFG	2	3,95	0,5093	1,386	0,1179	0,8824	71,84	615	< 44	< 4,8	76,2	54	71,3	48,1	< 88
102	HFG	3	7,17	0,9365	1,056	0,1518	0,5216	55,5	522	< 39	< 4,4	47,2	39	82,5	107,9	< 87
102	HFG	4	6,93	0,8312	1,072	0,1644	0,8804	54,94	410	< 39	< 4,9	63,3	38	70,6	68,9	< 84
102	HFG	5	7,37	0,9525	1,303	0,1798	0,3393	61,11	415	< 40	< 5,0	48,2	40	79	149,1	< 90
1419	HFG	1	4,34	0,3079	0,2566	0,0739	0,3324	67,66	625	< 43	< 4,8	149,4	38	64,6	177,5	< 70
1419	HFG	2	6,52	0,9839	1,127	0,2192	0,6239	58,53	792	< 40	< 4,7	61,6	38	72,1	23,7	< 82
1419	HFG	3	3,26	0,6978	0,7345	0,2998	0,402	81,67	879	54	< 5,2	108,6	56	67,4	35,6	< 89
1419	HFG	4	6,36	0,9986	1,088	0,2192	0,5861	58,31	731	53,7	< 4,5	62,3	38	75,7	34,4	< 80
1419	HFG	5	2,01	0,404	0,3273	0,1212	0,1046	70,92	514	< 44	< 4,5	93,8	38	77,4	30,8	< 75
1485	HFG	1	21,23	0,8306	0,6719	0,6093	0,01649	24,96	344	84,7	41,1	21,7	47,5	400,9	41,5	156
1485	HFG	2	7	1,023	0,4042	0,1853	< 0,0036	64,35	363	< 42	22,1	44,3	47	90,5	22,9	88
1485	HFG	3	6,71	1,074	0,2826	0,1821	< 0,0034	58,2	313	< 39	21,8	31,8	45	87,5	11,2	106
1485	HFG	4	2,32	0,1703	0,2353	0,0811	0,0461	84,33	386	< 48	< 5,0	85	48	77,9	20,4	< 85
1485	HFG	5	4,39	0,2046	0,2731	0,0799	0,0976	71,72	292	< 44	< 4,2	81,7	44	101,4	50,5	84
1585	HFG	1	6,68	0,6251	0,6842	0,1143	0,0698	65,37	532	< 42	< 4,6	80,4	38	63,7	52,3	< 76
1585	HFG	2	5,6	0,4586	0,4624	0,0734	0,0544	72,55	322	< 43	< 4,9	94,7	68	65,4	36,5	< 84
1585	HFG	3	5,85	0,5988	0,6234	0,1194	0,0626	69,35	562	< 43	< 4,6	93,4	38	72,2	84,3	< 80
1585	HFG	4	6,75	0,8154	0,6692	0,0998	0,0831	68,42	366	< 42	< 4,9	88,7	60	60,7	38,2	2,9
1585	HFG	5	8,4	0,8033	0,7289	0,0831	0,0809	60,68	288	< 39	< 4,6	70,4	55	66,1	35,4	< 82
1734	HFG	1	7,78	0,9803	1,194	0,218	0,3723	63,57	261	< 40	< 4,9	40,6	45	62,5	31,6	< 84
1734	HFG	2	7,27	0,7998	1,086	0,202	0,422	64,21	201	< 41	< 4,6	42,8	52	73,8	57,4	< 84
1734	HFG	3	8,63	1,018	0,9534	0,2127	0,2949	54,21	218	< 38	< 4,5	31,4	38	59,1	32	< 79
1734	HFG	4	12,42	1,849	1,511	0,2793	0,1371	48,31	435	< 36	< 4,5	39,7	43	73,8	35,9	< 76
1734	HFG	5	9,41	1,9	1,006	0,2934	0,2423	55,55	584	40	< 4,8	58,8	60	69,9	38,7	< 84
1752	HFG	1	10,14	0,8201	1,665	0,263	0,1116	56,98	230	< 38	< 5,0	115,4	65	62,9	34,4	< 83
1752	HFG	2	9,96	0,9032	1,382	0,3481	0,1565	57,04	420	< 39	< 4,9	128,7	65	77	49,3	< 1,0
1752	HFG	3	8,75	0,6558	1,227	0,2765	0,1672	59,07	360	< 39	< 4,7	121,1	45	70,5	52,5	< 81
1752	HFG	4	11,43	1,036	1,713	0,3073	0,1382	53,62	280	< 38	< 5,0	99,7	68	58	21,4	< 83
1752	HFG	5	9,14	0,984	1,466	0,3517	0,1812	57,18	587	< 39	< 5,3	133	52	67,9	20,6	< 83
2017	HFG	1	3,56	0,4311	0,5713	0,285	0,0085	69,67	409	11	14,4	63,4	46	50	486	< 76

2017	HFG	2	5,01	0,5868	0,5358	0,2909	< 0,0037	65,11	363	< 42	19,4	48,5	47	46	214,5	115
2017	HFG	3	4,99	0,4008	0,5135	0,1881	< 0,0039	72,14	461	7,1	15,8	72,5	46	56	128,1	83
2017	HFG	4	4,83	0,4699	0,6171	0,2909	0,0248	67,72	353	43	13	58,3	38	50	716,1	86
2017	HFG	5	4,28	0,4278	0,4987	0,1814	< 0,0040	74,46	398	< 45	12,8	73,4	66	46	617,6	95
2047	HFG	1	7,63	0,6241	0,6031	0,2086	0,022	64,82	1067	< 1,0	15,5	71,2	52	66,5	46,4	< 82
2047	HFG	2	7,92	0,5512	0,6118	0,2036	0,0087	63,25	1001	< 42	18,2	69,5	46	58,3	32	< 80
2047	HFG	3	6,54	0,4533	0,6877	0,2112	0,0163	67,95	1045	11	13,9	106,9	50	58,8	26	< 83
2047	HFG	4	8,87	0,6118	0,6843	0,2401	0,00832	61,34	1024	4,7	18,9	67,7	38	54,4	36	< 79
2047	HFG	5	6,42	0,7478	1,214	0,2188	0,075	71,04	1234	< 45	< 4,7	88,8	46	60,4	49,8	< 83
2282	HFG	1	4,31	0,4414	0,3613	0,3719	0,1909	73,72	1165	< 46	< 5,0	150	46	64	40,4	< 80
2282	HFG	2	3,52	0,3904	0,3317	0,3488	0,1883	74,83	1161	< 46	< 5,3	159,4	47	55	28,7	< 81
2282	HFG	3	2,74	0,3412	0,2538	0,3151	0,13	75,23	1140	< 46	< 4,7	132,7	60	63,5	42	< 80
2282	HFG	4	2,63	0,4052	0,2991	0,3842	0,1917	71,35	1140	< 45	< 4,9	115,6	51	56,2	14,3	< 77
2282	HFG	5	< 2,1	0,3194	0,272	0,1694	0,3172	86,48	1458	< 51	< 5,6	198,8	60	68,8	38,8	< 88
2375	HFG	1	2,65	0,3799	0,1438	0,1127	0,00514	81,67	627	< 48	5,5	99,7	69	56,9	40,2	91
2375	HFG	2	< 2,0	0,2963	0,1592	0,1176	0,0291	83,66	733	< 49	< 5,2	110,8	40	46	6,6	< 81
2375	HFG	3	< 2,0	0,2623	0,1421	0,1309	0,0194	82,35	717	50	< 4,6	108,6	59	62,4	43,5	199
2375	HFG	4	3,3	0,3267	0,2127	0,1165	0,00477	70,41	726	< 43	14	77,1	38	47	16,1	< 75
2375	HFG	5	< 2,0	0,245	0,1561	0,1355	0,0283	82,91	805	17	< 4,8	113,4	44	54	32,5	5,8
32	CG	1	13,64	0,7931	1,852	0,4413	< 0,00010	39,36	827	105	45,4	29,3	38	72,8	1,1	< 81
32	CG	2	11,94	1,114	1,974	0,6009	< 0,0029	41,88	757	126	50,1	28	38	72,9	< 2,2	< 84
32	CG	3	12,94	1,52	2,392	0,706	< 0,0030	44,15	793	112	66,6	41,2	38	109,8	19,5	< 96
32	CG	4	13,07	0,8088	1,573	0,736	< 0,0031	47,92	827	84	49	51,3	38	98,3	21	< 90
32	CG	5	15,37	0,6648	1,051	0,7927	< 0,0027	34,19	612	104	57,1	17,7	38	88,9	< 2,3	< 89
809	CG	1	16,01	0,7491	2,307	1,283	< 0,0029	41,86	758	78,1	49,9	44,1	38	84,1	41,8	< 87
809	CG	2	31,56	1,151	1,483	1,983	0,03451	8,429	525	111	112,1	7,4	41,4	229	46,1	< 90
809	CG	3	17,81	1,297	1,341	0,8427	0,01385	23,88	477	86,8	51,4	17,2	38	71,5	19,3	< 83
809	CG	4	18,64	1,274	1,382	0,8101	0,01281	22,57	468	96,9	45,5	13,1	38	67,5	11,3	< 80
809	CG	5	16,65	1,129	1,328	0,659	0,00861	30,41	661	106	45,9	26,9	38	68,7	12,2	< 81
1510	CG	1	8,66	0,5138	0,8266	0,1259	< 0,0023	25,4	287	70	57,5	17,1	40	70,8	6,6	243
1510	CG	2	24,57	0,906	1,269	0,3307	0,00648	24,55	1432	119	68,4	34,1	38	69,5	22,2	< 105
1510	CG	3	24,49	0,891	1,341	0,2547	< 0,0022	21,29	230	45,7	56,3	9,2	38	60,8	3,5	< 106
1510	CG	4	19,47	0,8695	1,301	0,2593	< 0,0022	23,38	285	73,2	60,6	16,5	45	71,8	17,5	< 88
1510	CG	5	12,54	0,3942	0,859	0,1594	< 0,0022	23,74	354	106	45,8	15,8	38	54,4	< 2,1	105
1580	CG	1	23,24	1,893	1,572	1,067	0,0721	24,29	1046	119	42	49,7	38	76,4	16,7	< 84
1580	CG	2	15,2	1,208	1,221	0,7762	0,0331	40,21	1277	117	33,7	48,1	38	63,3	10,4	< 92
1580	CG	3	16,61	1,621	1,152	0,7765	0,0518	41,21	1330	< 35	27,6	99	38	86,3	39,9	< 85
1580	CG	4	14,97	1,037	0,9813	0,7303	0,0532	39,91	884	120	22,8	52,4	38	92,6	19,2	< 78
1580	CG	5	17,32	1,232	1,089	0,446	0,1061	37,8	1313	< 34	12	82	38	62,3	30,5	< 79
1628	CG	1	28,88	1,914	2,033	0,2813	0,06626	15,02	170	42	55,9	6,9	45,8	80,7	23,7	< 107
1628	CG	2	29,21	1,746	1,879	0,2594	0,0708	14,49	185	52,2	44,9	7	44,2	82,2	10,1	< 105
1628	CG	3	26,3	1,978	2,068	0,298	0,0846	17,9	220	44	43,7	11,3	47,1	76,9	4,2	< 103
1628	CG	4	25,88	2,109	2,145	0,3083	0,0919	19,48	182	47,3	34,2	14,6	51,5	72,7	7,7	< 102
1628	CG	5	25,2	1,632	1,922	0,255	0,0912	17,53	208	51,2	39,3	14,4	54,7	64,1	21	< 101
1666	CG	1	22,71	1,109	1,928	0,159	< 0,0018	15,57	836	57,1	48,5	18	43,7	74,5	2,4	< 92
1666	CG	2	17,16	0,6873	1,465	0,1461	< 0,0022	22,8	1058	37,9	39,8	16,8	38	66,5	< 2,2	90
1666	CG	3	32,61	1,516	3,002	0,2191	< 0,0017	12,59	693	70,2	66,2	12,2	42	82	< 3,0	< 102
1666	CG	4	23,23	1,41	3,047	0,2652	< 0,0024	26,99	1569	85,6	54,4	24,8	38	94,9	< 2,9	< 108
1666	CG	5	18,06	1,06	2,344	0,1848	0,00277	29,04	1513	108	48,1	26,9	38	78,6	< 2,6	< 96
101	FS	1	41,52	0,5988	1,941	0,1193	0,07579	6,297	77	< 15	56,4	6,4	42,1	93,9	< 3,2	< 120
101	FS	2	41,93	0,427	1,673	0,0953	0,07472	6,146	87	< 14	58,1	5,4	38	83,7	< 3,1	< 118
101	FS	3	31,58	0,4646	5,064	0,046	0,1717	10,83	236	< 18	20,8	9,9	42,6	83,8	3,6	< 114
101	FS	4	38,92	0,6584	1,829	0,1101	0,0984	7,139	114	< 15	44,1	6,1	43,9	87,1	< 3,2	< 116
101	FS	5	41,17	0,5038	1,951	0,1115	0,08356	6,802	77	< 15	60,5	7,7	41	97,5	1,5	< 119
385	FS	1	13,62	1,987	1,509	0,36	0,2017	37,56	176	< 31	< 5,4	23,7	63,1	124,9	133,5	< 99
385	FS	2	14,9	1,859	1,443	0,319	0,1805	34,21	157	< 30	< 5,1	21,2	49	113,3	111,1	< 91
385	FS	3	19,34	2,862	2,76	0,5078	0,1596	25,41	177	27,5	6,1	14,4	65,3	178,8	162,4	< 91
385	FS	4	20,99	3,278	9,703	0,6457	0,5378	20,33	334	63,1	< 5,8	17,3	51,2	254,1	144,7	< 94
385	FS	5	17,6	2,424	1,737	0,4184	0,1537	26,05	105	< 26	7	12,3	58,9	164,4	121,9	< 91
965	FS	1	22,98	0,3331	1,128	0,1804	0,0897	26,7	548	98,7	28,9	20,1	38	98,7	24,9	< 93
965	FS	2	23,05	0,3486	0,7154	0,1396	< 0,0025	28,67	162	< 28	42,6	9,7	38	48	< 1,0	< 91
965	FS	3	21,91	0,3379	1,133	0,167	< 0,0025	30,56	172	< 28	40,2	7,7	38	46	1,5	< 91
965	FS	4	20,3	0,4299	1,834	0,1256	< 0,0026	33,43	167	< 29	36,4	12	38	46	8,5	< 91
965	FS	5	23,35	0,3905	1,358	0,2115	0,0981	27,91	539	75,9	25,2	25,3	38	84,6	2,7	< 96
973	FS	1	19,81	0,5651	1,963	0,1004	< 0,0023	27,82	205	< 27	37,5	8,8	38	52,4	15,1	< 91
973	FS	2	23,8	1,141	3,445	0,2699	< 0,0020	20,31	157	< 23	58,4	11,9	39	79,4	53,9	< 93
973	FS	3	26,37	0,904	3,054	0,1297	< 0,0021	21,3	139	< 23	49,6	10,4	38	66,1	44,7	< 99
973	FS	4	23,67	0,5168	2,013	0,1074	< 0,0023	26,39	202	< 26	51,8	12,1	38	59,2	24,9	< 94
973	FS	5	20,28	0,6838	2,213	0,1046	< 0,0022	25,14	136	< 25	45,9	12,3	38	61	24,9	< 87
1577	FS	1	36,41	0,6713	2,322	0,1878	0,0018	9,473	77	< 16	68,8	3,4	56,8	81,7	< 3,3	< 117
1577	FS	2	22,86	0,6374	0,9044	0,173	< 0,0022	22,76	163	< 25	56	7,9	55,5	68,8	195,8	< 110
1577	FS	3	27,1	1,109	1,277	0,2268	< 0,0021	19,79	206	< 24	58,5	3,4	43	66,1	5,5	< 111
1577	FS	4	25,48	1,058	0,9687	0,152	0,00345	17,37	116	< 22	59	4,2	56,3	55,9	< 2,8	< 103
1577	FS	5	31,67	0,4108	1,994	0,118	< 0,0016	11,19	80	< 18	75,5	4,2	48,9	60,2	14,7	< 115

1679	FS	1	43,72	0,5976	1,256	0,1037	< 0,0010	4,148	77	< 12	95,6	7,5	42,8	63,1	< 3,5	< 122
1679	FS	2	41,53	0,5306	1,182	0,1087	< 0,0011	4,52	77	< 13	95,6	6,2	43,7	66	13,9	< 124
1679	FS	3	41,17	0,1542	0,9886	0,118	< 0,0010	4,201	77	< 12	104,1	7,2	44,4	60,9	4,3	< 124
1679	FS	4	41,7	0,1141	1,221	0,166	< 0,00010	4,282	77	< 12	92,2	8,3	44,9	65,6	6,9	< 119
1679	FS	5	41,52	0,3901	1,51	0,2497	< 0,0012	5,376	77	< 14	119	9	47,7	65,7	14,6	< 131
436	PFG	1	32,57	0,1374	1,073	0,0581	< 0,0013	7,657	77	< 14	50	3,4	38	47	< 2,8	< 96
436	PFG	2	16,47	0,3777	2,075	0,1033	0,0446	21,96	77	1,8	31,3	3,7	38	48	4,8	< 92
436	PFG	3	7,88	0,4597	2,336	0,1905	< 0,0031	53,5	78	< 36	30,8	26,1	41	58,1	23,3	129
436	PFG	4	11,45	0,4601	2,644	0,1196	< 0,0027	40,38	83	< 30	38,1	15,4	46	55,9	21,3	75
436	PFG	5	21,54	0,3712	2,119	0,0928	0,04269	18,81	77	< 22	35,2	4,9	40	50	8,2	< 94
1281	PFG	1	3,82	0,8799	0,3442	0,9818	0,032	64,22	1517	< 44	15,5	92,9	34	2601	34,6	8600
1281	PFG	2	2,59	0,4396	0,3205	0,5834	0,0089	69,72	1434	< 45	18,2	82,1	40	1120	47,7	2160
1281	PFG	3	2,06	0,2934	0,3376	0,6722	0,0172	58,85	1202	< 41	18,3	60,4	38	1127	10,4	2580
1281	PFG	4	< 1,7	0,5574	0,3815	0,5616	0,0287	50,18	1534	< 39	24,2	64,9	38	2596	42,3	10620
1281	PFG	5	4,05	0,7623	0,3906	0,7018	0,00531	61,98	1498	< 43	24,5	80,1	35	1975	40,6	6710
1603	PFG	1	5,68	0,1339	1,109	6,363	0,5245	58,51	< 66	< 42	< 5,9	25,6	38	51	< 2,1	< 72
1603	PFG	2	4,55	0,1175	0,7834	1,343	0,0721	71,78	1312	61	< 6,0	33,4	38	46	25,1	< 74
1603	PFG	3	5	0,1765	0,7647	2,337	0,1416	65,01	1077	67	< 5,1	24,1	49	46	2,1	< 73
1603	PFG	4	2,94	0,0987	0,6645	1,478	0,1025	77,72	1374	79	< 5,5	39,6	46	46	< 2,2	< 79
1603	PFG	5	5,93	0,1226	0,8352	4,45	0,3094	66,68	377	< 45	< 6,0	35,7	38	46	< 2,4	< 74
1812	PFG	1	4,16	0,42	1,225	0,175	< 0,0030	55,88	294	< 38	22,2	24	38	46	8,7	236
1812	PFG	2	7,31	0,465	0,6686	0,1336	< 0,0028	47,77	156	< 35	28,3	20,6	38	54,8	29,3	171
1812	PFG	3	4,02	0,4337	0,8709	0,11	< 0,0037	73,05	165	< 44	16,6	50,4	48	52	30,7	< 79
1812	PFG	4	3,94	0,4629	0,7946	0,1301	< 0,0037	75,16	159	69	18,1	47,4	58	50	35,4	< 79
1812	PFG	5	5,2	0,4385	0,8538	0,1559	< 0,0034	65,42	257	< 41	23	35,9	40	58,3	18	262
1985	PFG	1	16,73	1,041	2,464	0,4236	0,0903	37,18	202	64,7	17,5	35,9	41	65	48,1	< 79
1985	PFG	2	5,69	0,77	2,434	0,0951	0,33	54,69	172	< 37	< 4,1	42,3	38	56,4	16,5	< 71
1985	PFG	3	11,57	1,145	2,62	0,4245	0,1589	51,65	300	44,1	< 4,9	51,1	40	60	29	< 84
1985	PFG	4	5,01	0,7146	2,461	0,0913	0,3597	56,03	145	12	< 4,0	41,5	38	64,9	23,4	< 73
1985	PFG	5	15,5	1,072	2,384	0,4441	0,094	40,43	228	72,2	13	34,5	38	60,6	22,4	< 80
XP1	Natural ochre	1	6,65	0,678	1,036	0,2064	0,3544	50,49	581	< 56	< 12	3,4	329,7	78,4	< 3,7	< 1,0
XP1	Natural ochre	2	11,5	1,263	0,7391	0,3955	0,0786	36,22	225	48	< 11	3,4	266,2	68,2	< 3,5	< 122
XP1	Natural ochre	3	6,49	0,856	0,7761	0,2411	0,4555	45,22	224	< 51	< 11	3,4	295,2	69,6	< 3,7	< 1,0
XP1	Natural ochre	4	6,75	0,87	1,062	0,2402	0,3943	53,74	325	< 57	< 13	3,4	332,6	79,7	< 3,9	< 1,0
XP1	Natural ochre	5	14,15	1,935	0,6751	0,4467	0,4545	31,88	367	105	< 12	3,4	235,5	75,9	< 3,5	< 109
XP1	Natural ochre	6	6,91	1,761	0,8272	0,2346	0,3655	45,67	207	< 51	< 13	3,4	305,3	71	< 3,8	< 116
XP3	Natural ochre	1	31,55	< 0,019	0,0435	1,528	< 0,0013	4,997	158	35,4	50,3	< 5,1	< 5,1	63,7	28,1	< 97
XP3	Natural ochre	2	11,63	1,158	16,9	0,3113	0,011	34,96	< 44	< 12	35,2	37,6	41,7	52,1	37,8	< 195
XP3	Natural ochre	3	29,51	0,321	0,1502	1,625	0,02306	7,003	183	46,5	78	< 5,1	< 5,1	81,2	38,6	< 106
XP4	Natural ochre	1	41,77	0,0455	0,2162	1,08	0,03558	3,882	266	56,6	83,2	3,4	42,9	117,4	11,3	< 134
XP4	Natural ochre	2	32,21	1,235	0,2168	1,211	0,07102	4,284	157	75,3	50,4	< 5,1	< 5,1	87,3	25,4	< 95
XP5	Natural ochre	1	36,88	0,487	0,2469	1,277	< 0,0014	6,335	156	< 14	93,1	< 5,1	8,4	45	12,1	< 96
XP5	Natural ochre	2	40,13	< 0,022	0,4076	1,292	< 0,0014	7,112	200	< 14	32,8	< 5,1	< 5,1	39,5	16,1	< 94
XP6	Natural ochre	3	11,8	1,301	16,44	0,3095	0,0121	33,26	< 43	< 12	29,7	27,8	36	45,8	43,7	< 187
XP6	Natural ochre	4	13,21	1,214	14,08	0,3148	0,0069	28,6	< 40	< 11	23,5	27,6	51,2	47,3	58,9	< 174
Geo1	Natural ochre	1	12,27	1,438	0,4864	0,3344	0,0804	36,3	164	< 44	< 11	3,4	272,5	68,2	< 3,4	2,4
Geo1	Natural ochre	2	10,19	0,985	1,637	0,2774	0,1599	43,38	531	123	< 11	3,4	271	80,8	41	< 1,0
Geo2	Natural ochre	1	9,71	1,1	0,798	0,3038	0,0152	45,93	166	< 52	< 11	3,4	324,8	61,8	< 3,7	< 128
Geo2	Natural ochre	2	11,04	1,102	0,4963	0,4007	0,1104	41,62	334	67	< 12	3,4	298	65,7	< 3,7	5,9
Geo4	Natural ochre	1	9,1	0,913	0,5933	0,2932	0,046	44,02	239	< 50	< 12	3,4	301,1	68,3	< 3,7	< 123
Geo5	Natural ochre	1	8,45	0,962	0,4747	0,3044	0,4082	43,44	316	< 51	< 12	3,4	299,2	67,2	< 3,6	< 116
Geo 6	Natural ochre	1	20,57	1,18	1,16	0,3899	0,0625	74,3	323	< 36	178,8	154,3	76	22,1	< 22	340
Geo 6	Natural ochre	2	3,47	0,294	0,5542	0,1467	0,0304	61,28	586	21	< 12	3,4	399	63,3	< 3,3	< 118
Geo 6	Natural ochre	3	13,4	0,927	0,759	0,3432	0,0341	54,91	345	< 36	128,5	103,9	69	24,2	< 16	< 213
Laga1	Natural ochre	1	60,3	< 0,015	0,2418	2,536	< 0,0011	4,492	150	21,4	234,6	4,3	38	155,1	20,6	303
Laga1	Natural ochre	2	63,6	< 0,017	0,4879	2,51	< 0,0019	14,93	259	29,8	167,2	14,5	38	103,1	24,2	< 101
Laga1	Natural ochre	3	69,8	0,1068	0,5916	3,796	< 0,0017	10,19	272	86	151,4	9,4	40	105,3	31,3	< 99
Laga2	Natural ochre	1	81,1	0,2402	1,32	1,574	< 0,0015	7,179	77	137	120,2	3,5	43,7	50	14,4	< 103
Laga2	Natural ochre	2	78,1	0,3198	0,9037	1,797	< 0,0015	7,798	77	158	114,2	3,4	45,7	53,3	12,7	< 101
Laga2	Natural ochre	3	73,6	0,3254	2,797	1,788	< 0,0016	8,502	120	169	117,8	5,7	46,9	60,3	22,5	< 102
Laga3	Natural ochre	1	14,7	< 0,020	0,8225	0,26	< 0,0039	67,72	569	< 35	27,9	32,2	38	59,5	< 1,9	266
Laga3	Natural ochre	2	5,8	< 0,017	0,2687	0,034	< 0,0040	67,03	136	< 33	24,6	25,8	38	47	8,1	232
Laga3	Natural ochre	3	31,15	< 0,017	0,282	0,5946	< 0,0034	52,28	582	< 31	42,2	26,8	38	72,9	12	102,7
Laga4	Natural ochre	1	44,1	1,689	33,22	0,33	0,0861	4,954	77	< 5,9	54,2	3,4	53,5	86,8	6,4	< 98
Laga4	Natural ochre	2	37,75	1,778	38,85	0,4687	0,1765	5,654	77	< 5,8	40,1	3,4	54,4	107,5	19,4	< 94
Laga4	Natural ochre	3	41,54	2,247	40,26	0,4661	0,262	5,81	77	< 6,7	24,1	3,4	58,3	92,9	14,2	< 92
Laga5	Natural ochre	1	79	< 0,016	0,3486	1,709	< 0,0013	5,783	81	41,2	143,8	7,4	38	92,7	1,6	< 107
Laga5	Natural ochre	2	85	< 0,016	0,3357	2,198	0,02008	6,002	124	14,7	140,6	3,4	41,9	124,2	26,5	< 109
Laga5	Natural ochre	3	85,8	< 0,016	0,5036	2,34	0,01084	6,779	132	24,1	156,4	3,4	40	123,5	15,3	< 107
Laga6	Natural ochre	1	85,3	0,3182	4,341	0,7519	0,02319	10,57	129	< 14	70,4	11,2	43,4	83,8	17,4	< 99
Laga6	Natural ochre	2	84	0,3363	1,911	0,653	0,00292	9,022	88	< 13	60,8	10,3	40	75,8	7,1	< 94
Laga6	Natural ochre	3	76,6	0,1661	1,307	0,7307	< 0,0018	12,14	168	< 15	73,3	15,4	39	75	7,5	< 93
Laga7	Natural ochre	1	36,53	0,0782	9,427	4,079	0,2408	34,8	821	< 22	13,2	10,2	38	72,9	31	< 78
Laga7	Natural ochre	2	37,78	< 0,018	4,817	5,272	0,1961	42,51	761	< 27	12	18,9	38	67,3	28,1	< 75

Laga7	Natural ochre	3	35,95	0,0908	12,31	2,291	0,2741	27,27	593	*< 19*	9,7	6,8	38	62,2	2,5	*< 77*
Laga8	Natural ochre	1	74,2	5,011	1,123	0,1462	0,01928	7,117	77	*< 11*	88,2	3,4	184,5	52,3	75,4	*< 100*
Laga8	Natural ochre	2	65	4,184	0,8092	0,149	0,02033	6,334	77	*< 11*	84,6	3,4	169,8	52	67,3	143,6
Laga8	Natural ochre	3	67,1	4,582	0,5884	0,1593	0,0355	6,874	77	*< 10*	82,7	4,6	168,2	53,2	66,3	*< 84*
Laga9	Natural ochre	1	59,5	*< 0,014*	1,115	1,672	0,125	11,94	292	*< 16*	63,8	3,4	38	59,3	2,6	*< 82*
Laga9	Natural ochre	2	59,8	0,1029	0,9687	1,917	0,1641	20,45	469	*< 21*	39,4	3,4	38	67	6,8	*< 89*
Laga9	Natural ochre	3	55,9	*< 0,016*	0,6096	2,21	*< 0,0023*	21,16	384	*< 21*	95,9	9,1	38	53,5	*< 2,4*	*< 86*
Laga10	Natural ochre	1	32,25	0,0307	0,603	4,261	0,4734	37,99	900	75,1	*< 4,6*	14,2	38	70,2	19,9	*< 72*
Laga10	Natural ochre	2	31	*< 0,017*	0,4404	4,821	0,1034	29,46	709	97,6	46,3	6,8	38	66,1	26,7	129
Laga10	Natural ochre	3	37,24	0,0567	0,6626	4,838	0,3318	30,26	761	106	*< 4,2*	10,2	38	70,4	33,1	*< 75*
Laga11	Natural ochre	1	72,4	0,0735	0,8662	2,787	*< 0,0015*	7,461	188	19,1	189	7,2	40,2	150,3	18	*< 106*
Laga11	Natural ochre	2	71,2	0,3673	2,086	2,565	0,00252	7,708	178	25,4	178,3	6,9	41,8	134,2	19,7	*< 107*
Laga11	Natural ochre	3	74,9	0,1279	3,025	2,387	0,00279	7,506	181	22,5	166	8,2	39	130,1	11,1	*< 107*
Laga12	Natural ochre	1	89,6	*< 0,015*	0,5607	0,8761	0,02982	9,244	100	*< 13*	59	5,5	40	132,1	34,9	*< 100*
Laga12	Natural ochre	2	85,3	*< 0,016*	1,31	1,335	0,04164	11,95	163	*< 16*	58,5	7,9	40	127	30,7	*< 102*
Laga12	Natural ochre	3	86,2	*< 0,014*	0,5045	1,051	0,00356	8,33	97	*< 14*	69,8	3,4	39	144,9	23,5	*< 99*
Laga13	Natural ochre	1	76	0,0525	0,8315	2,03	0,01773	6,029	112	109	136,1	3,4	42,6	106,5	18,8	*< 108*
Laga13	Natural ochre	2	85,1	0,0394	1,032	2,103	*< 0,0014*	6,107	132	50,2	154,3	3,4	40	124	25,4	*< 114*
Laga13	Natural ochre	3	76,8	*< 0,017*	0,4821	2,04	*< 0,0013*	6,046	103	28,3	162,1	3,4	38	121,7	11,3	*< 109*
Laga14	Natural ochre	1	54,5	*< 0,015*	0,3067	2,748	*< 0,0013*	5,937	191	*< 13*	212,7	3,5	39	151,9	21,4	344
Laga14	Natural ochre	2	49,5	*< 0,015*	0,2504	2,585	*< 0,0012*	5,215	168	12,6	218,2	3,4	39	123,8	22,7	471
Laga14	Natural ochre	3	74	0,0252	0,7561	2,807	*< 0,0013*	6,463	173	*< 13*	232,3	3,4	40,3	183,6	38,3	*< 109*
Laga15	Natural ochre	1	61,5	*< 0,0001*	0,5017	0,9405	0,00192	10,87	231	88,8	143,3	5,6	41	61,7	15,3	*< 98*
Laga15	Natural ochre	2	41,68	*< 0,018*	0,5057	1,087	*< 0,0027*	37,31	759	89,8	67	21,9	38	65,2	28,6	*< 84*
Laga15	Natural ochre	3	50,9	*< 0,014*	0,2573	2,087	*< 0,0021*	21,11	552	86,4	94,1	14	38	66,3	10,2	*< 79*
Laga16	Natural ochre	1	35,9	0,2149	0,7582	4,287	*< 0,0020*	18,84	620	*< 21*	139,7	39	37	574	24,4	1218
Laga16	Natural ochre	2	39,5	0,0686	0,438	3,523	*< 0,0024*	27,65	429	31,3	107,3	26,6	38	55,1	30,9	109
Laga16	Natural ochre	3	42,1	0,0709	0,4066	3,601	*< 0,0021*	22,9	450	*< 22*	107,6	29,5	45,2	60,7	39,7	100
Laga17	Natural ochre	1	34,94	0,1204	6,295	1,285	0,0486	33,42	897	140	52	39,1	38	560,5	17,1	590
Laga17	Natural ochre	2	26,31	0,1741	14,49	1,334	0,0319	45,67	1104	137	36,2	40,8	38	335,8	22,8	206
Laga17	Natural ochre	3	28,23	0,3116	9,815	1,162	*< 0,0035*	59,69	2966	135	37,9	81,6	38	209,2	8,5	*< 70*
Laga18	Natural ochre	1	70,4	*< 0,013*	0,0968	0,423	*< 0,0017*	11,02	77	*< 14*	80,6	6,1	38	65,6	6,6	*< 95*
Laga18	Natural ochre	2	74,7	*< 0,015*	0,3917	0,3942	*< 0,0019*	17,82	77	*< 19*	52,9	4,2	38	52,9	14,9	*< 91*
Laga18	Natural ochre	3	72,3	*< 0,014*	0,1019	0,4259	*< 0,0019*	15,33	77	*< 16*	69,2	7,8	40	62,7	19,5	*< 95*
Laga19	Natural ochre	1	91,1	0,0309	1,805	1,415	*< 0,0015*	8,725	109	*< 14*	82,9	8	40	66,6	6,4	*< 93*
Laga19	Natural ochre	2	97,6	*< 0,016*	0,2695	1,212	*< 0,0012*	5,32	77	*< 11*	123,2	4,6	38	58,3	3,2	*< 104*
Laga19	Natural ochre	3	86,4	0,1134	9,616	2,105	*< 0,0013*	7,125	96	*< 11*	98,1	4,2	41	75,9	12,4	*< 106*
Laga20	Natural ochre	1	64,6	*< 0,015*	0,3093	2,629	*< 0,0018*	12,76	272	39,4	154	6,6	38	98,1	19,6	*< 93*
Laga20	Natural ochre	2	53,3	0,04	0,3209	2,86	*< 0,0018*	11,56	252	*< 16*	149,8	7,4	41,5	103,1	35,6	210,4
Laga20	Natural ochre	3	64,1	*< 0,017*	0,2743	2,936	0,02676	5,596	156	28,4	221,2	4,1	38	123,3	33,6	*< 103*
Laga21	Natural ochre	1	60,7	*< 0,015*	1,541	1,155	*< 0,0018*	14,28	406	42,9	99,4	5,8	38	74,3	6,2	*< 81*
Laga21	Natural ochre	2	49,3	*< 0,015*	0,6407	0,8383	*< 0,0023*	25,29	1042	73,2	61,3	13,8	38	63,1	9,1	*< 77*
Laga21	Natural ochre	3	55,7	0,1181	5,713	1,33	*< 0,0019*	19,28	635	72,7	118	10,2	38	81,8	1,5	*< 92*
Laga22	Natural ochre	1	46,6	*< 0,017*	0,6	1,099	0,00725	24,3	666	155	79,1	7	38	60,7	15,6	*< 88*
Laga22	Natural ochre	2	52,7	*< 0,017*	0,5982	2,242	0,00652	10,57	251	40,2	184,4	5,8	38	114,3	21,1	266
Laga22	Natural ochre	3	52,6	0,0878	0,6367	1,787	*< 0,0020*	17,92	452	94,8	129	12,8	38	86,1	10,1	*< 88*
Laga23	Natural ochre	1	70,1	*< 0,017*	0,6192	2,896	0,151	6,126	237	*< 14*	115,9	3,4	39,9	210,6	37	*< 112*
Laga23	Natural ochre	2	70,6	*< 0,017*	0,5516	2,965	0,04707	6,29	171	22,4	190,7	3,4	40,9	202,8	39,1	*< 110*
Laga23	Natural ochre	3	69	*< 0,016*	0,4311	2,71	*< 0,0011*	4,925	150	*< 12*	223,6	3,6	38	177,5	21,7	*< 107*
Laga24	Natural ochre	1	60,3	*< 0,017*	0,4789	2,984	0,08814	5,928	204	27,7	166,7	3,4	38	199,8	30,9	149
Laga24	Natural ochre	2	64,6	*< 0,015*	0,7063	2,305	*< 0,0012*	5,065	117	*< 11*	201,5	3,4	39	147,9	20,2	145,2
Laga24	Natural ochre	3	62,1	*< 0,016*	0,4927	2,729	0,09116	5,324	178	14,3	161,4	3,4	40,4	180,1	30,3	*< 104*
Laga25	Natural ochre	1	29,91	0,1404	0,8461	0,5543	*< 0,0033*	45,7	939	102	33,8	30,3	38	74,8	3,4	119,5
Laga25	Natural ochre	2	41,32	0,1814	1,181	1,013	*< 0,0033*	44,28	887	94,8	40,1	27,7	38	106,6	7,5	*< 74*
Laga25	Natural ochre	3	48,81	0,5672	2,508	1,64	0,0426	28,85	976	156	54,4	34,9	40	131,8	11	*< 72*
Laga26	Natural ochre	1	72,4	0,1097	0,9462	2,642	*< 0,0012*	5,16	167	*< 12*	216,3	3,4	39	166,9	36,3	*< 111*
Laga26	Natural ochre	2	72,1	0,1325	1,212	2,694	*< 0,0012*	5,125	160	2,9	235,1	5	38	184,5	27,1	*< 114*
Laga26	Natural ochre	3	69,6	0,0773	0,8288	2,66	*< 0,0012*	5,126	128	16,2	233,3	3,4	39,5	195	33,3	*< 106*
Laga27	Natural ochre	1	62,3	*< 0,016*	0,4535	3,121	0,02077	7,985	212	38,2	196,1	6,9	38	159,3	18,5	136
Laga27	Natural ochre	2	63,4	*< 0,017*	0,3002	3,244	*< 0,0012*	5,772	180	24	251,2	4,2	38	142,3	31	171,6
Laga27	Natural ochre	3	61,3	*< 0,016*	0,3168	3,079	*< 0,0013*	6,571	208	*< 13*	224,4	4,3	38	161,4	17	201,1
Laga28	Natural ochre	1	61	0,0954	1,507	2,255	0,01646	18,14	335	2	100,1	3,4	42,4	76,6	14,6	*< 92*
Laga28	Natural ochre	2	60,8	0,1132	1,634	2,229	0,03312	17,49	280	*< 19*	89,8	8,3	41	73,6	15,2	*< 91*
Laga28	Natural ochre	3	52,03	0,0316	0,6041	1,876	0,0433	31,71	478	31,3	59,4	16	42	70,4	23,5	*< 87*
Laga29	Natural ochre	1	74	*< 0,017*	0,7717	2,164	*< 0,0014*	6,748	127	39,3	175,4	3,4	39	124,1	9,7	*< 108*
Laga29	Natural ochre	2	77	*< 0,015*	0,6075	1,713	*< 0,0012*	5,177	113	*< 12*	139,1	3,7	38	101,5	8,9	*< 111*
Laga29	Natural ochre	3	80,7	0,021	0,6694	1,582	0,02183	5,125	78	19,4	136,5	3,4	41,3	115	25	*< 107*

Figures in italics indicate concentrations below the limit of detection. Num: number; raw mat: raw material; SFG: soft fine-grained; BFG: banded fine-grained; HFG: hard fine-grained; CG: coarse-grained; FS: ferruginous sandstone; PFG: platy fine-grained.

S5 Supplementary Table 2. Detailed results of SEM-EDS analyses conducted on ochre pieces from Porc-Epic Cave.

Num	Raw mat	Num of an	Description of analysed item				Semi-quantitative EDS analyses **				Interpretation***
			Grain morph	BSE cont*	Length range (µm)	Width range (µm)	>10%	10-3%	3-1%	<1%	
17	SFG	3	Irr	W	1-2	1	**Fe**	(Si, Ca)	(Al, K)	(Mg, Cl) Mn (P, Ti, S, Na)	Iron ox (+ clay min + silicate)
17	SFG	2	Plat	G	3-4	2-3	(Fe) Si	Al, Ca	K, Mg (Cl)	P, S (Ti) Na	Clay min (+ iron ox)
17	SFG	2	Irr	DG	Submicr	Submicr	Si (Fe)	-	(Ca, K, Al)	(Mg, Cl, S, P, Na)	Silicate (+ iron ox + clay min)
17	*SFG*	*3*	*-*	*W/DG*	*-*	*-*	*Fe, Si*	*Ca, K*	*Al*	*Mg (Cl) Ti, S, Na, P*	*Mixture of iron ox and alum*
295	SFG	3	Irr/aggl	W	1-3	submicr-1	Fe	(Si, Ca, S, Al)	(K)	(Cl, Mg, P, Ti)	Iron ox (+ clay min)
295	SFG	1	Irr	G	6	5	(Fe) Si	(Al)	(K, Ca, Cl, Mg)	(Ti, S, P)	Silicate (+ iron ox)
295	SFG	3	Irr/subcirc	G	4-11	2-11	Ca, S (Fe)	-	(Si, Al)	(P, K, Cl, Mg, Na)	Calcium sulph (+ iron ox + clay min)
295	*SFG*	*3*	*-*	*G/LG*	*-*	*-*	*Fe*	*Si, Al, Ca*	*(Cl) K, S, Na*	*Mg, P, Ti, Na*	*Mixture of iron ox and alum*
538	SFG	1	Plat	LG	Submicr	Submicr	**Fe**	(Si)	(Al, Ca)	(K, Cl, Ti, Mg, P)	Iron ox (+ clay min)
538	SFG	3	Irr/ang	W/LG	4-8	1-7	**Fe**	(Si, Al)	(Ca)	(K, Cl, Mg, Ti, Na, S)	Iron ox (+ clay min)
538	SFG	1	Irr	LG	29,92	21,64	Si (Fe)	(Al)	(Ca)	(K, Cl Mg, Ti, Na)	Silicate (+ iron ox)
538	SFG	1	Aggl	G	Submicr	Submicr	Si, Al	Fe, Na, K	Ca	(Cl) Mg	Clay min (+ iron ox)
538	SFG	1	Plat	G	28,24	12,84	Si, Al	Fe, K	Ca (Cl)	Mg (Ti) Na	Mica (+ iron ox)
538	SFG	1	Subcirc	W	1,84	1,81	Ni (Fe)	(P)	(Si, Al)	(Ca, Cl, K, Ti, Mg)	Undet Ni-rich particle (+ iron ox)
538	*SFG*	*4*	*-*	*W/G*	*-*	*-*	*Fe (Si)*	*(Al)*	*(Ca, K, Cl)*	*(Mg, Ti, Na, P, S)*	*Mixture of iron ox and alum*
913	SFG	2	Irr	W	Submicr	Submicr	**Fe**	(Si, Ca, Cl)	(Al, K)	(P, Mg) Mn (Ti, S)	Iron ox (+ clay min + silicate)
913	SFG	2	Irr/aggl	W	4-11	4-10	Fe (Si)	(Cl, Ca, Al)	(K)	(Mg, P, S, Ti) Mn (Na)	Iron ox (+ clay min + carbonates)
913	SFG	1	Plat	G	26,83	16,67	(Fe) Si	(Cl, Ca, Al)	(K, S)	(Mg, Na, P, Ti)	Silicate (+ iron ox)
913	SFG	1	Aggl	LG	8,07	5,93	(Fe) Si	(Cl) Ca, Al	K	Mg, Ti, P	Silicate (+ iron ox)
913	SFG	1	Irr	LG	19,36	8,94	Si (Fe)	Al, Ca (Cl)	K, Mg	(Ti, P) Na	Mica (+ iron ox)
913	SFG	1	Ang	G	12,9	11,41	Si, Al	(Fe) Na, Ca	K (Cl)	Ti, Mg, P	Na-rich feldspar (+ iron ox)
913	*SFG*	*4*	*-*	*G*	*-*	*-*	*Fe, Si*	*(Cl) Ca, Al*	*K*	*Mg, P, S, Ti, Na*	*Mixture of iron ox and alum*
919	SFG	2	Irr/subcirc	W	Submicr-1	Submicr-1	**Fe**	(Si, Al)	(Cl, Ca, P, K)	(Mg, Ti, S)	Iron ox (+ silicate)
919	SFG	3	Irr	DG	7-8	3-5	**(Fe)**	Si (Al)	(Ca, Cl, P)	(K, Mg, Ti, Na, S)	Silicate (+ iron ox)
919	SFG	1	Aggl	W	Submicr	Submicr	**Ba** (Fe)	S	(Si, Al, Ca)	(Cl, K, P, Na, Mg)	Ba sulph (+ iron ox + alum)
919	SFG	1	Ang	G	27,65	6,92	(Fe) Ca, S	(Si)	(Al, Cl, P, K)	(Mg)	Calcium sulph (+ iron ox)
919	*SFG*	*3*	*-*	*G/LG*	*-*	*-*	*Fe, Si*	*Al, Ca (Cl)*	*P, K*	*Mg, Ti*	*Mixture of iron ox and alum*
919	*SFG*	*1*	*-*	*W*	*-*	*-*	*(Fe) Ba*	*(Si, Al)*	*(Cl) S (Ca, P)*	*(Ti, K, Mg, Na)*	*Mixture of Ba sulph, iron ox and alum*
930	SFG	2	Irr	W	2-3	1-2	Fe (Si)	(Al, Ca, Cl)	(K)	(Mg, P, Ti, Na)	Iron ox (+ clay min)
930	SFG	1	Aggl	W	7,05	5,38	(Si) Fe	(Ca, Al, Cl)	(K)	(Mg, P, Na)	Iron ox (+ alum)
930	SFG	1	Acic	W	4	0,8	**Fe**	(Si, Ca, Al)	(Cl, K)	(Mg, P, Ti)	Iron ox (+ clay min)
930	SFG	2	Irr/Aggl	G/DG	Submicr	Submicr	Si (Fe)	Al, Ca, K	(Cl) P	Mg, Ti	Clay min (+ iron ox)
930	SFG	1	Irr	DG	27,15	11,85	Si (Fe)	Al, K, Ca	(Cl)	P, Mg, Na, S	K-rich feldspar (+ clay min)
930	*SFG*	*2*	*-*	*LG/DG*	*-*	*-*	*Fe, Si*	*Al, Cl (Ca)*	*K*	*Mg, P, Ti, Na*	*Mixture of iron ox and alum*
987	SFG	1	Aggl	LG	Submicr	Submicr	**Fe** (Si)	-	Ba, Al, Ca) Mn (C	(K, S, Mg, P)	Iron ox (+ silicate + Ba sulph)
987	SFG	1	Ang	W	8,28	5,36	**Fe** (Si)	-	Al, Ba) Mn (Ca, C	(K, Mg, S, P, Na)	Iron ox (+ silicate)
987	SFG	3	Ang/plat	DG/B	6-37	3-14	(Fe) Si	-	(Al, Ba, Ca)	(Cl, K, S, Mg, P)	Silicate (+ iron ox + Ba sulph)
987	SFG	2	Tab	W	7-10	5-10	**Ba**	(Fe) S (Si)	Ca, Al	(Cl, K, Mn, Na, Mg)	Ba sulph (+ iron ox + clay min)
987	*SFG*	*3*	*-*	*W/DG*	*-*	*-*	*Fe, Si, Ba*	*-*	*Ca, Al, S (Cl) Mn*	*K, P, Mg, Na*	*Mixture of iron ox, alum and Ba sulph*
994	SFG	4	Plat/aggl	W/LG	Submicr	Submicr	**Fe** (Si)	(Al)	(K, Ca)	(Cl, Mg, Na, Ti, P, S)	Iron ox (+ clay min + silicate)
994	SFG	5	Irr	W	2-4	1-2	**Fe** (Si)	(Al)	(Ca, K)	(Cl, Mg, Na, P, Ti, S)	Iron ox (+ clay min)
994	SFG	1	Irr	DG	Submicr	Submicr	(**Fe**) Si	Al	K, Ca	(Cl) Mg, P	Clay min (+ iron ox)
994	SFG	1	Plat	LG	2	2	(**Fe**) Si	Al	K, Ca	(Cl) Mg, P	Clay min (+ iron ox)
994	SFG	1	Plat	LG	16,32	9,39	(**Fe**) Si	Al	K, Ca	(Cl) Mg, Na, P	K-rich mica (+ iron ox)
994	*SFG*	*4*	*-*	*W/G*	*-*	*-*	*Fe, Si*	*Al*	*K, Ca*	*Mg, (Cl), Ti, Na, P, S*	*Mixture of iron ox and alum*
1635	SFG	5	Irr/aggl	W/G	1-4	1-2	**Fe**	(Ca, Si)	(Cl) Mn (Al, S)	(K, Mg, P, Ti, Na)	Iron ox (+ clay min + calcium sulph)
1635	SFG	1	Irr	LG	20,96	10,62	**Fe**	-	(Ca, Si) Mn (Cl)	(Al, S, K, Mg, Ti, P)	Iron ox (+ clay min)
1635	SFG	2	Aggl	G/LG	2	2	(Fe) Si, Ca	Al	(Cl) K, P	Mg, Na (Mn) Ti, S	Clay min (+ iron ox)
1635	SFG	1	Irr	LG	4,77	4,1	**(Fe)**	Si (Ca)	(Cl, Al, Ba, Mn)	(K, S, Mg, P)	Silicate (+ iron ox)
1635	SFG	3	Aggl	W/G	3-4	2-3	(Fe) Ca, S	-	(Si, Cl)	(Al, P, Mn, K, Mg, Na, Ti)	Calcium sulph (+ iron ox)
1635	*SFG*	*3*	*-*	*W/G*	*-*	*-*	***Fe***	*Ca, Si (Cl)*	*Al, Mn*	*K, P, Mg, S, Na, Ti*	*Mixture of iron ox and alum*
1914	SFG	1	Aggl	W	1,82	1,01	Fe	(Ba, Si, Ca)	(S, Al, Cl, K)	(Mg, P, Na)	Iron ox (+ Ba sulph + clay min)
1914	SFG	2	Aggl	G	5	3-4	Fe	(Ca, Si, S, Cl)	(Al, K, Ba)	(Mg, Ti, P, Na)	Iron ox (+ Ba sulph + clay min)
1914	SFG	3	Irr	G	2-4	2-4	(Fe) Si	(Al, Ca)	(Cl, K, Mg)	(Ti, S, Na, P)	Silicate (+ clay min)
1914	SFG	3	Plat/aggl	W	1-4	1-4	Ba (Fe)	S (Si, Ca)	(Al, Cl)	(K, Na, Mg, P, Mn)	Ba sulph
1914	*SFG*	*3*	*-*	*G*	*-*	*-*	*Fe, Si*	*Ca (Cl) Al*	*K*	*Mg, S, P, Ti, Na, Mn*	*Mixture of iron ox and alum*
1914	*SFG*	*1*	*-*	*W*	*-*	*-*	*Fe, Si*	*Ba, Ca (Cl)*	*Al, S, K*	*Ti, Mg, P, Na*	*Mixture of Ba sulph, iron ox and alum*
1927	SFG	2	Aggl	W	2	2	**Fe**	(Si)	(Al, Ca, Cl)	(K, Ti) Mn (Mg, S, Na, P)	Iron ox (+ clay min)
1927	SFG	3	Irr/aggl	W/G	5-8	4-8	Fe	(Ca, Si, Al Cl)	(K, P)	(Mg, S, Ti, Na)	Iron ox (+ alum + clay min)
1927	SFG	3	Aggl	G	3-4	2-3	Ca (Fe, Cl)	(Si)	(Al)	(K, Mg, P, S, Na, Mn, Ti)	Carbonate (+ iron ox + clay min)
1927	*SFG*	*3*	*-*	*W/G*	*-*	*-*	***Fe***	*Si, Ca (Cl) Al*	*K*	*Mg, Mn, P, Ti, S, Na*	*Mixture of iron ox and alum*

306	BFG	2	Irr/plat	W	Submicr	Submicr	Fe (Si)	(Al, Cl)	(Ca, K, Mg)	Mn (P, Ti, S)	Iron ox (+ clay min)
306	BFG	1	Tab	W	22,85	8,52	**Fe**	(Si, Al, Cl)	(Ca, K)	(Mg, P, Ti)	Iron ox (+ clay min)
306	BFG	1	Irr/plat	G	Submicr	Submicr	(**Fe**) Si	Al (Cl)	Ca, K, Mg	Mn, P, Ti, S	Clay min (+ iron ox)
306	BFG	2	-	G/LG	-	-	**Fe**	Si, Al (Cl)	Ca, Mg	K, Mn, Ti, P, S	Mixture of iron ox and alum
521	BFG	2	Aggl	W/LG	Submicr	Submicr	**Fe**	-	(Si, Ca)	n (Al, Cl, K, Mg, P, Ti, S, N	Iron ox (+ clay min)
521	BFG	4	Irr	W	1-3	1-2	**Fe**	(Si, Ca)	(Al, Cl, K)	(Mg, P, Ti) Mn (S)	Iron ox (+ clay min)
521	BFG	1	Plat	G	Submicr	Submicr	(Fe) Si	Al	Ca (Cl)	K, Mg, Ti, P	Clay min (+ iron ox)
521	BFG	1	Plat	DG	Submicr	Submicr	(**Fe**) Si	(Ca)	(Cl, K, Al)	(Mn, P, Ti, Mg, S)	Silicate (+ iron ox + clay min)
521	BFG	2	Subcirc	G	1-2	Submicr-1	(Fe) Si	(Al, Ca)	(K, Cl)	(Mg, P, Na, S)	Silicate (+ iron ox + alum)
521	BFG	2	Plat	LG	9-17	7-8	(Fe) P	Ca (Si, Cl)	(Al, K)	(Mg, Ti, Na, S)	Calcium phosphate (+ iron ox)
521	BFG	1	Irr	DG	16,16	12,95	(Fe) Ca (Cl	(Si)	(Al, K, S)	(P, Ti, Mg, Na)	Carbonate (+ iron ox + alum)
521	BFG	3	-	W/G	-	-	**Fe**	Si, Ca	(Cl) Al	K, Mg, P, S, Mn, Ti, Na	Mixture of iron ox and alum
102	HFG	2	Irr	W	Submicr	Submicr	**Fe**	(Si)	(Al)	Mn (Ca, K, Mg, Cl, Ti, P)	Iron ox (+ clay min)
102	HFG	5	Irr	W	4-7	2-4	**Fe** (Si)	(Al)	(K, Mg)	(Ca) Mn (Cl, Ti, P)	Iron ox (+ clay min)
102	HFG	1	Irr	G	15	10	Si	(Fe)	(Al)	(K, Ca, Mg, Cl)	Silicate (+ iron ox)
102	HFG	5	Ang/plat	G/LG	18-80	8-21	Si (Fe)	-	Al	K, Ca, Cl, Mg	Mica (+ iron ox)
102	HFG	2	-	LG/G	-	-	**Fe**	Si	Al, K, Ca	(Cl) Mn, Mg, Ti, S, P	Mixture of iron ox and alum
1419	HFG	2	Irr	G	2-3	1	**Fe** (Si)	(Al)	(K, Ca)	(Mg) Mn (Cl, Ti, P)	Iron ox (+ clay min)
1419	HFG	5	Plat	W	4-5	1-3	**Fe**	(Si)	(Al)	Ca, Mg) Mn (Cl, Ti, Na, P	Iron ox (+ clay min)
1419	HFG	1	Ang	G	8	6,40	Si (Fe)	-	(Al, K)	(Mg, Ca, Cl, P)	Silicate (+ iron ox)
1419	HFG	1	-	W	-	-	**Fe**	Si, Al	-	; Mg, Mn, Ca (Cl) Ti, Na, I	Mixture of iron ox and alum
1419	HFG	1	-	G	-	-	**Fe**	Si, Al	K	Ca, Mg, Mn (Cl) Ti, P, Na	Mixture of iron ox and alum
1419	HFG	1	-	DG	-	-	**Fe**	Si, Al	K	Mg, Ca, Mn (Cl) Ti, P, Na	Mixture of iron ox and alum
1752	HFG	2	Aggl	W	Submicr	Submicr	**Fe**	(Si)	(Al, Ca)	(K, Cl, Mg, P, Ti, Na)	Iron ox (+ clay min)
1752	HFG	2	Plat	W	7	5-6	Fe (Si)	(Al)	(K, Ca, Mg, Cl)	(Ti, P, Na)	Iron ox (+ clay min)
1752	HFG	1	Aggl	DG	Submicr	Submicr	Si (Fe)	Ca, Al	K, P (Cl) Mg	Ti, S, Na	Clay min (+ iron ox)
1752	HFG	2	Aggl	G	7-14	5-9	Si (Fe)	(Al)	(Ca, K, Cl)	(Mg, P, Ti, Na)	Silicate (+ iron ox)
1752	HFG	1	-	LG	18,72	0,74	(Si)	(Fe, Al, K, Ca)	(Cl, Mg)	(Ti, P)	Fibrous material (*contamination*)
1752	HFG	4	-	W	-	-	Si, Fe	Al, Ca	K (Cl) Mg	Ti, P, Na	Mixture of iron ox and alum
1510	CG	7	Aggl /Subcirc	W/LG	Submicr	Submicr	**Fe**	(Si, Al)	(Ca, Cl)	(S, K, Ti, Mg, P) Mn	Iron ox (+ silicate)
1510	CG	1	Plat	W	15,06	10,24	**Fe**	(Si)	(Al, Ca)	(Cl, K, S, Ti, Mg)	Iron ox + (silicate)
1510	CG	1	Subcirc	DG	28,85	20,88	Si (Fe)	(Al)	(Ca)	(K, Cl, Mg, S)	Silicate (+ iron ox)
1510	CG	2	Plat	LG	16-17	5-11	(**Fe**)	Ca, S (Si)	(Al)	(Cl, K, Ti, Mg, Mn, P)	Calcium sulph (+ iron ox + silicate)
1510	CG	3	-	W/DG	-	-	**Fe**, Si	Al	Ca (Cl)	S, K, Mg, Ti, P	Mixture of iron ox and silicates
1628	CG	2	Plat	W	1-2	1	**Fe**	(Si)	(Al)	(Ca, K, S, Mg, Ti, Na, P)	Iron ox (+ silicate)
1628	CG	2	Plat	G	2	1-2	Si	(Fe) Al, Ca	K (Cl) Mg	Na, Ti, P	Clay min (+ iron ox + silicate)
1628	CG	2	Subcirc	G	23-1126	16-169	Si	(Fe, Al)	(K, Ca, Mg, Cl)	(Ti, Na)	Silicate (+ iron ox + clay min)
1628	CG	1	Plat	G	17,41	11,58	Si, Al	Ca, Na	(Fe)	K (Cl) Mg	Ca-rich feldspar (+ iron ox + silicate)
1628	CG	1	-	G	-	-	**Si**	-	(Fe, Al)	(K, Ca, Cl, Mg)	Silicate
1628	CG	3	-	W/LG	-	-	Si, Fe	(Al)	(Ca, Cl, K)	(Mg, Ti, Na, P, S)	Mixture of iron ox and silicates
101	FS	2	Aggl	W	Submicr	Submicr	Fe (Si)	(Ca)	(Cl, Al)	(K) Mn (Mg, Na, P)	Iron ox (+ clay min + silicate)
101	FS	3	Irr/plat	W	3-4	1-3	Fe (Si)	(Ca)	(Cl, Al)	Mn (Mg, K, Ti, P)	Iron ox (+ clay min)
101	FS	3	Irr/plat	G	38-52	18-38	Si (Fe)	-	(Al, Ca, Cl)	(K, Na, Mg)	Silicate (+ iron ox + clay min)
101	FS	2	Tab	G	10	7-8	Si (Fe)	-	(Al, Ca, Cl)	(K, Mg, P)	Silicate (+ iron ox + clay min)
101	FS	2	-	W/DG	-	-	Fe, Si	-	Ca (Cl) Al	K, Mg, Mn, Na, P, Ti, S	Mixture of iron ox and silicates
973	FS	3	Plat	W	1-3	1	**Fe**	(Si, Ca)	(Al, Cl, K)	(Mg, Ti, S, Na, P)	Iron ox (+ silicate)
973	FS	2	Aggl	W	8-9	1-3	Fe (Si)	-	(Ca, Al)	(Cl, K, S, Ti, Mg, P)	Iron ox (+ silicate)
973	FS	1	Aggl	LG	Submicr	Submicr	Si	Ca, Fe (Cl)	Al, K	Mg, Ti, Na, S	Clay min (+ iron ox)
973	FS	3	Irr/ang	G	8-38	5-24	Si	(Fe)	(Ca, Al)	(Cl, S, Mg, K, Na)	Silicate (+ iron ox)
973	FS	1	Ang	LG	14,03	9,54	Ca, S	(Fe, Si)	(Al)	(Cl, K, Mg, P, Na)	Calcium sulph (+ iron ox + silicate)
973	FS	2	-	W/G	-	-	Fe, Si	Al, Ca	(Cl) K	S, Mg, P, Ti, Na	Mixture of iron ox and silicates/alum
436	PFG	2	Plat	W	2	Submicr	**Fe**	(Si)	-	(Ca, Cl, Al, S, Mg, P, K)	Iron ox (+ silicate)
436	PFG	2	Plat	W/LG	23-34	20	**Fe**	(Si)	(Ca, Cl, Al)	(K, S, Mg, P)	Iron ox (+ clay min)
436	PFG	2	Aggl	G	1-3	1-2	Si (Fe)	Ca (Cl) Al	-	K, Mg, S	Clay min (+ iron ox)
436	PFG	2	Irr	DG	2-3	1-2	**Si**	(Fe)	-	(Ca, Al, Cl, Mg)	Silicate (+ iron ox)
436	PFG	2	-	G	-	-	**Fe**	Si	Ca (Cl) Al	S, K, Mg, Ti, P	Mixture of iron ox and clay min
1812	PFG	3	Irr/aggl	G	Submicr	Submicr	**Fe**	(Si)	(Al, Ca)	(Mg, K, Cl, S, Ti, Ba, P)	Iron ox (+ clay min)
1812	PFG	4	Plat/tab	W/G	45-78	11-48	**Fe**	(Si)	(Al, Ca)	Mg, K, Cl, S, Ti, P, Ba, Na	Iron ox (+ clay min)
1812	PFG	1	Acic	LG	32,67	1,59	**Fe**	(Si)	(Al, Ca, Mg)	(K, S, Cl, P, Ti, Na)	Iron ox (+ clay min)
1812	PFG	2	Tab	W	11-18	2-4	**Ba (Fe)**	S	(Si)	(Al, Ca, Na, K, Mg)	Ba sulph (+ iron ox)
1812	PFG	3	-	W/G	-	-	**Fe**	Si	Al, Ca	Mg, K, S (Cl) Ti, P, Na	Mixture of iron ox and alum

Num: number; raw mat; raw material; an: analyses; morph: morphology; cont: contrast; EDS: energy dispersive X-ray spectroscopy; SFG: soft fine-grained; BFG: banded fine-grained; HFG: hard fine-grained; CG: coarse-grained; FS: ferruginous sandstone; PFG: platy fine-grained; irr: irregular; plat: platy; aggl: agglomerate; subcirc: subcircular; acic: acicular; tab: tabular; clay min: clay mineral; W: white; G: grey; DG: dark grey; LG: light grey; submicr: submicrometric; ox: oxide; min: mineral; sulph: sulphate.
* BSE cont. refers to the contrast observed on backscattered electron images.
** Elements in brackets play no role in the mineralogical composition of the analysed items, elements in bold are present in a proportion equal to or greater than 40%.
*** Text in brackets indicates the interpretation of detected elements surrounding the analysed area.

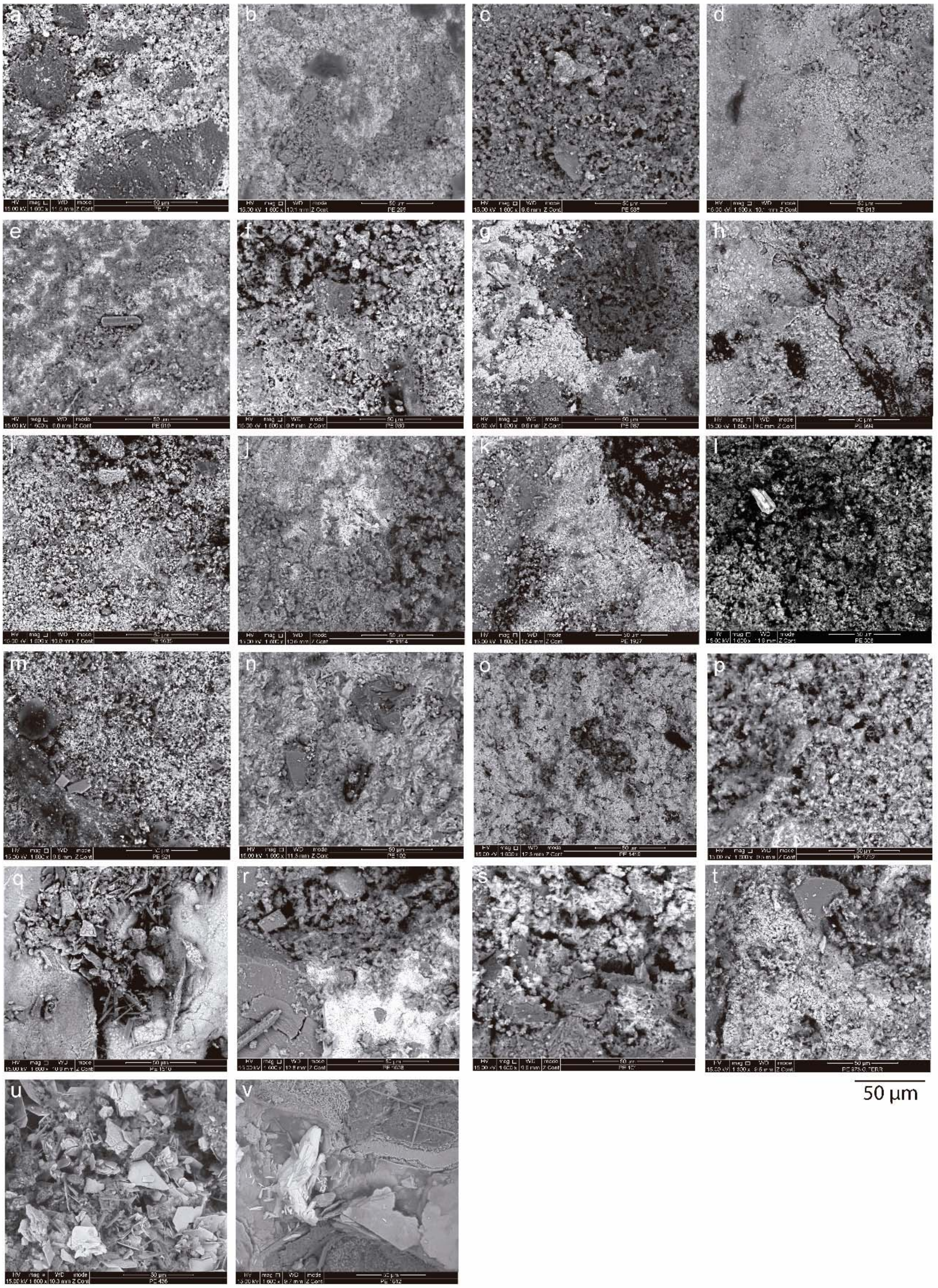

S5 Supplementary Figure 1. SEM images of ochre pieces from Porc-Epic Cave.
All figures are in backscattered electron (BSE) mode. a: ochre piece PE17, SFG; b: ochre piece PE295, SFG; c: ochre piece PE538, SFG; d: ochre piece PE913, SFG; e: ochre piece PE919, SFG; f: ochre piece PE930, SFG; g: ochre piece PE987, SFG; h: ochre piece PE994, SFG; i: ochre piece PE1635, SFG; j: ochre piece PE1914, SFG; k: ochre piece PE1927, SFG; l: ochre piece PE306, BFG; m: ochre piece PE521, BFG; n: ochre piece PE102, HFG; o: ochre piece PE1419, HFG; p: ochre piece PE1752, HFG; q: ochre piece PE1510, CG; r: ochre piece PE1628, CG; s: ochre piece PE101, FS; t: ochre piece PE973, FS; u: ochre piece PE436, PFG; v: ochre piece PE1812, PFG. Scales = 50 µm.

Elemental composition and texture by raw materials

Pieces attributed to HFG (Fig. 4n-p; Table 2; S5 Supplementary Fig. 1n-p; S5 Supplementary Table 2) show a matrix rich in small Fe particles (submicrometric to 7 µm in length) containing few larger agglomerates or irregular grains (7-14 µm in length) rich in Si and submicrometric grains rich in Si, Al and Ca, that were interpreted as silicates and clay minerals respectively.

PFG ochre pieces (Fig. 4u,v; Table 2, S5 Supplementary Fig. 1u,v; S5 Supplementary Table 2) are mostly composed of particles with a very high proportion of Fe. These particles, interpreted as iron oxides, mainly come in three shapes: (1) platy particles, the most frequent, variable in size (2-78 µm in length); (2) large acicular particles (32 µm in length); (3) agglomerates of irregular submicrometric particles. Other less frequent compounds found in the Fe-rich matrix include: agglomerates of small (1-3 µm in length) Si, Al and Ca-rich grains interpreted as clay minerals, small (1-2 µm in length) irregular grains rich in Si interpreted as silicates, and tabular grains (11-18) rich in Ba and S interpreted as Ba sulphates.

CG ochre pieces (Fig. 4q,r; Table 2, S5 Supplementary Fig. 1q,r; S5 Supplementary Table 2) are characterised by large particles different in morphology and composition: (1) subcircular grains rich in Si (23-1126 µm in length), (2) platy particles rich in Si, Al, Ca (17 µm in length), and (3) platy particles (16-17 µm in length) rich in Ca and S. We interpret them as silicates, feldspars and calcium sulphates respectively. Smaller platy grains (2 µm in length) rich in Si, Al and Ca, are interpreted as clay minerals. Fe-rich grains, interpreted as iron oxides, appearing in the form of agglomerates of submicrometric grains and larger platy particles (1-15 µm in length), were also identified.

FS ochre pieces (Fig. 4s,t; Table 2, S5 Supplementary Fig. 1s,t; S5 Supplementary Table 2) feature irregular, angular and platy grains of variable size (8-30 µm in length), rich in Si, interpreted as silicates. These grains are surrounded by agglomerates of Fe-rich particles of variable size (submicrometric to 9 µm in length) featuring in some cases a platy morphology. Other identified particles include submicrometric agglomerates of particles rich in Si, Ca, Fe and Al interpreted as clay minerals and, more rarely, angular grains (14 µm in length) rich in Ca and S, interpreted as calcium sulphates.

The analysis of **SFG pieces** (Fig. 4a-k; Table 2, S5 Supplementary Fig. 1a-k; S5 Supplementary Table 2) revealed that this raw material, which appears homogeneous macroscopically, is in fact the most heterogeneous in composition and texture. Distinctive

features are agglomerates of small particles of iron oxides (submicrometric to 4 µm), mostly irregular in shape but also including platy, and more rarely, acicular morphologies. Many pieces also feature agglomerates of larger iron oxide particles (5 to 30 µm in length), irregular or angular in shape. These agglomerates are often embedded in or surrounded by other particles. Among these particles, those composed of Si are the most frequent. They generally appear in the form of irregular and more rarely platy or angular particles of variable size (submicrometric to 37 µm). Agglomerates of small platy and irregular grains rich in Si and Al, interpreted as clay minerals, are also detected. Other less frequent components include platy or irregular particles rich in Al and K with a length ranging from 16 to 28 µm, interpreted as micas. A few pieces are rich in irregular or angular grains (12-27 µm in length), mostly composed of Si, Al, K, interpreted as feldspars. Agglomerates of Ca sulphates (3-27 µm in length) in the form of small grains or larger angular grains, and Ba sulphates (submicrometric to 10 µm in length) were also identified. A single specimen (ochre piece PE538) shows the presence of an undetermined Ni-rich particle, interpreted as contamination.

BFG pieces (Fig. 4l,m; Table 2, S5 Supplementary Fig. 1l,m; S5 Supplementary Table 2) fall into the compositional and textural variability of SFG pieces. The main difference lies in a macroscopic feature: BFG pieces show layers of different colours. BFG also lacks micas, feldspars and sulphates. Such a difference, however, could be attributed to the few pieces of BFG analysed. As already mentioned for SFG, this raw material is characterised by the presence of iron oxides in the form of agglomerates of submicrometric grains, small (1-2 µm in length) irregular grains or larger (22 µm in length) tabular particles. Submicrometric agglomerates of grains interpreted as clay minerals, rich in Si, Al, Ca, and small platy and subcircular (submicrometric to 2 µm in length) silicates are also identified. On a single piece, large grains (9-16 µm) rich in Ca, P and Ca respectively are interpreted as Ca phosphates and carbonates.

S6 – Ochre pieces: mineralogical data

S6 Supplementary Table 1. Results of XRD and μ-XRD analyses on Porc-Epic Cave's ochre pieces and natural samples from the surrounding area.

Num	Raw mat	Type of an	ab	an	cal	gp	gth	hem	kln	mgh	mnt	ms	qz	sa
37	SFG	μ-XRD			•			•	•	•	•	•	•	
282	SFG	μ-XRD			•			•	•		•		•	
304	SFG	μ-XRD					•	•	•		•	•	•	
334	SFG	μ-XRD			•	•	•	•			•	•	•	
363	SFG	μ-XRD						•				•	•	
386	SFG	μ-XRD			•			•	•		•		•	
392	SFG	μ-XRD			•			•			•	•	•	
615	SFG	XRD	•					•			•		•	•
710	SFG	XRD	•	•	•		•						•	
994	SFG	XRD			•			•	•				•	
1277	SFG	XRD						•					•	
1635	SFG	XRD	•		•		•	•	•				•	
1981	SFG	XRD	•					•					•	
177	BFG	μ-XRD			•		•	•	•	•	•	•	•	
934	BFG	XRD	•				•	•					•	
857	HFG	μ-XRD						•					•	
512	CG	μ-XRD						•	•		•	•	•	
1510	CG	XRD			•		•	•	•				•	
1628	CG	XRD						•					•	
366	FS	μ-XRD						•	•			•	•	
1679	FS	XRD	•					•					•	
436	PFG	XRD					•	•					•	
1812	PFG	XRD	•				•	•					•	•
Geo1	Natural ochre	μ-XRD			•		•	•	•	•	•	•	•	
Geo2	Natural ochre	μ-XRD					•		•			•	•	
Geo3	Natural ochre	μ-XRD					•	•	•			•	•	
Geo4	Natural ochre	μ-XRD					•	•	•			•	•	
Geo5	Natural ochre	μ-XRD						•	•			•	•	
Geo6	Natural ochre	μ-XRD						•	•			•	•	

Num: number; mat: material; type of an: type of analysis; SFG: soft fine-grained; BFG: banded fine-grained; HFG: hard fine-grained; CG: coarse-grained; FS: ferruginous sandstone; PFG: platy fine-grained; XRD: X-ray diffraction; μ-XRD: micro-X-ray diffraction. Abbreviations of minerals are based on the nomenclature suggested by [116].

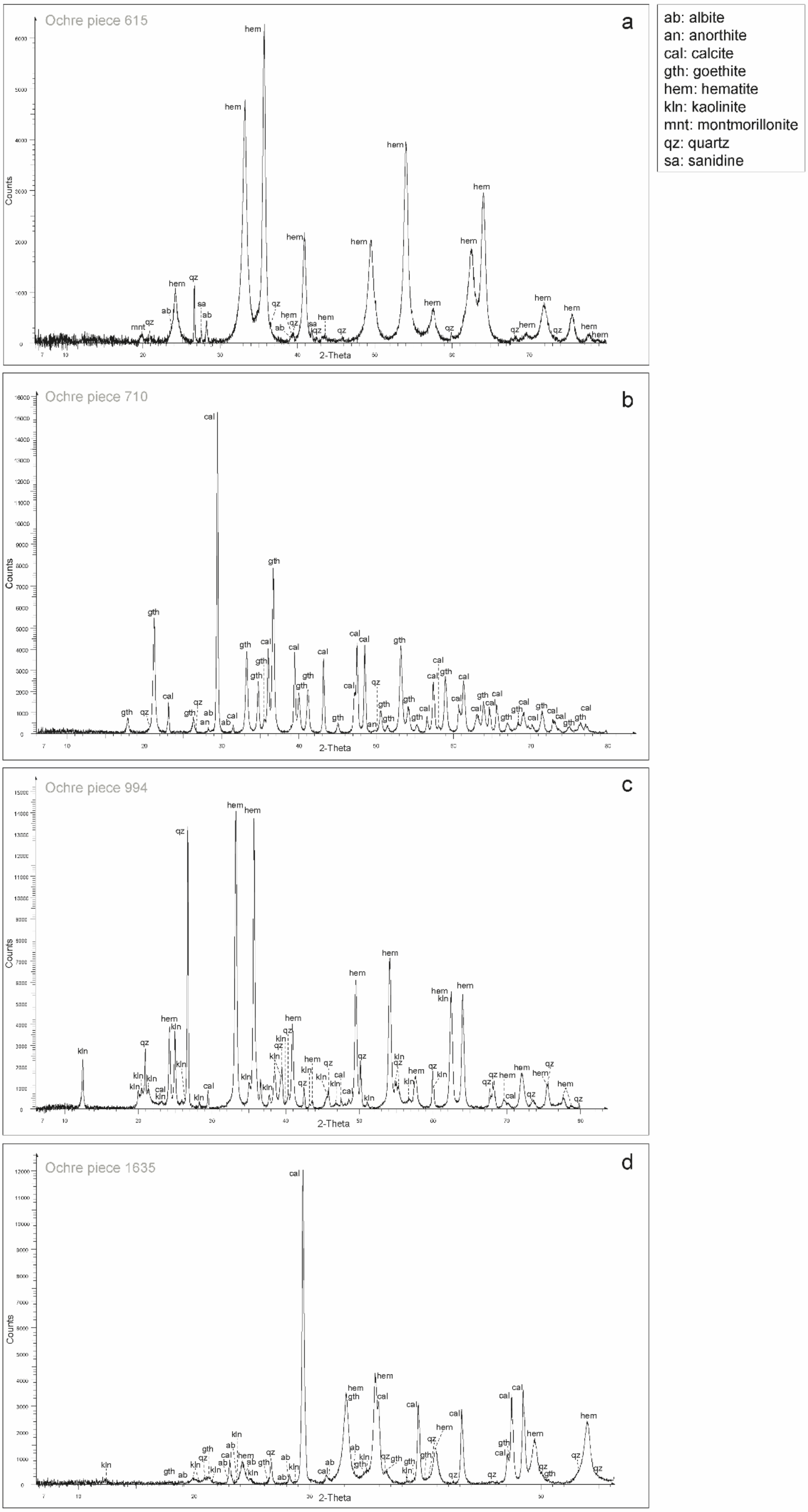

S6 Supplementary Figure 1. Representative X-ray diffractograms obtained from the analysis of Porc-Epic Cave ochre pieces.

a: ochre piece PE615; b: ochre piece PE710; c: ochre piece PE994; d: ochre piece PE1635.

S6 Supplementary Table 2. Summarised results of μ-Raman analyses conducted on Porc-Epic Cave's ochre pieces.

N	Raw mat	ab	an	brt	cal	fluo	gth	gp	hp	hem	ilm	kln	lep	mag	man	mc	mnt	ms	nt	phl	qz	rt	un Mn ox	C
9	SFG				•		•			•			•					•			•			
17	SFG		•				•	•		•											•			
51	SFG			•						•														
133	SFG									•		•												
295	SFG									•											•			
381	SFG									•			•								•			
538	SFG						•			•	•				•			•			•			
610	SFG							•		•														
615	SFG									•														•
710	SFG						•			•						•								
725	SFG									•														
726	SFG					•				•														
737	SFG		•							•											•			
913	SFG				•			•		•														
919	SFG						•	•		•														
930	SFG				•	•	•			•								•						
987	SFG									•														
994	SFG				•		•			•											•			•
1087	SFG	•			•					•			•					•			•			
1277	SFG									•														
1427	SFG		•			•				•														
1481	SFG		•			•				•														
1491	SFG		•			•				•							•	•						•
1493	SFG									•											•			
1526	SFG	•		•			•			•											•			
1552	SFG									•													•	
1566	SFG									•											•			
1626	SFG		•	•			•			•										•				
1635	SFG	•	•					•		•											•			
1637	SFG	•					•			•														
1677	SFG	•								•		•												
1699	SFG	•	•		•					•											•			
1737	SFG						•			•								•						
1780	SFG						•			•														
1845	SFG								•	•				•				•			•		•	
1862	SFG		•		•	•		•		•														
1914	SFG		•							•						•								
1927	SFG		•							•				•										
1942	SFG									•														
1981	SFG						•			•								•	•		•			
2023	SFG		•				•			•								•						
2063	SFG						•	•		•														•
2067	SFG									•														
2069	SFG									•														
2091	SFG						•			•											•	•		•
2159	SFG			•			•			•											•	•		
306	BFG						•			•														•
521	BFG						•			•								•						

901	BFG						•			•														
1499	BFG						•			•			•	•							•			
1700	BFG	•					•			•		•												
1806	BFG						•			•				•							•			
2104	BFG						•			•											•			
102	HFG					•	•			•											•			
1419	HFG									•													•	
1485	HFG	•						•		•											•			
1585	HFG						•			•														
1734	HFG									•														
1752	HFG									•														
2017	HFG							•		•														
2047	HFG									•														
2282	HFG		•				•	•		•														
2375	HFG									•														
32	CG		•				•			•						•					•			
809	CG									•								•			•			
1510	CG	•			•		•			•						•		•			•			
1580	CG									•			•								•		•	
1628	CG						•	•		•					•						•		•	
1666	CG				•		•			•											•			
101	FS		•				•			•								•			•			
385	FS						•			•				•							•			
965	FS									•											•			
973	FS						•			•											•			
1577	FS						•			•											•			
1679	FS	•	•			•				•								•			•			
436	PFG							•		•		•									•			
1281	PFG					•		•		•				•										
1603	PFG									•														
1812	PFG		•				•			•								•						
1985	PFG						•			•														

N: number; mat: material; un: undetermined; ox: oxide; C: Carbon. Abbreviations of minerals are based on the nomenclature suggested by [116], except for hp (hydroxyapatite); fluorapatite (fluo), lepidocrocite (lep), manganite (man) and natrojarosite (nt); SFG: soft fine-grained; BFG: banded fine-grained; HFG: hard fine-grained; CG: coarse-grained; FS: ferruginous sandstone; PFG: platy fine-grained.

S6 Supplementary Table 3. Detailed results of µ-Raman analyses conducted on Porc-Epic Cave's ochre pieces.

Num	Raw mat	Num of an	Morph	Grain colour	Identified compounds*
9	SFG	1	Aggl	B	hem (lep)
9	SFG	1	Aggl	B	hem (cal)
9	SFG	1	Irr	R	hem (ms)
9	SFG	1	Sub	Y	gth (hem)
9	SFG	1	Ang	T	qz (cal, hem, ms)
17	SFG	1	Aggl	R	hem
17	SFG	1	Irr	R	hem (an, gth)
17	SFG	2	Aggl	R/DR	hem (gp)
17	SFG	1	Aggl	B	hem (an, qz)
17	SFG	3	Ang/irr	T	qz (hem)
17	SFG	1	Aggl	W	qz (hem)
51	SFG	1	Aggl	B	hem
51	SFG	2	Aggl	W	brt
133	SFG	4	Aggl	R/DR	hem
133	SFG	1	Sub	B	hem (kln)
295	SFG	1	Aggl	R/DR	hem
295	SFG	1	Aggl	DR	hem
295	SFG	1	Aggl	DR	hem (qz)
381	SFG	3	Aggl	R/DR	hem
381	SFG	1	Aggl	DR	hem (qz, lep)
538	SFG	3	Aggl	DR	hem
538	SFG	1	Irr	B	hem (gth, ilm, man, qz)
538	SFG	1	Tab	Y	hem (ms)
610	SFG	4	Aggl	R/DR	hem
610	SFG	1	Aggl	DR	hem (gp)
615	SFG	3	Aggl	R	hem
615	SFG	2	Ang	DR/B	hem
615	SFG	1	Aggl	DR	hem
615	SFG	1	Ang	B	C (hem)
710	SFG	2	Aggl/sub	R	hem
710	SFG	1	Sub	B	hem
710	SFG	1	Aggl	G	gth
710	SFG	1	Aggl	Y	gth
710	SFG	1	Aggl	B	gth (mc)
725	SFG	3	Aggl	DR	hem
725	SFG	1	Ang	B	hem
726	SFG	1	Aggl	R	hem
726	SFG	2	Aggl	DR	hem (fluo)
737	SFG	1	Aggl	R	hem (an)
737	SFG	3	Aggl	DG	hem (an)
737	SFG	1	Aggl	R	hem (an, qz)
913	SFG	1	Aggl	DR	hem
913	SFG	3	Aggl	R/DR	hem (cal, gp)
913	SFG	1	Sub	B	hem (cal, gp)
919	SFG	2	Aggl	R/DR	hem
919	SFG	1	Sub	B	hem
919	SFG	2	Aggl	DR	hem (gth)
919	SFG	1	Aggl	DR	hem (gp)
930	SFG	1	Irr	DR	hem
930	SFG	2	Ang	B	gth
930	SFG	5	Aggl	Y	gth
930	SFG	1	Aggl	Y	gth (fluo)
930	SFG	2	Aggl	Y	gth (cal)
930	SFG	1	Plat	B	ms (gth)
987	SFG	2	Aggl	R/DR	hem
987	SFG	1	Sub	DR	hem
994	SFG	1	Sub	DR	hem
994	SFG	1	Aggl	R	hem (cal, C)
994	SFG	1	Aggl	B	gth (qz)
1087	SFG	1	Aggl	R	hem
1087	SFG	1	Aggl	B	hem (cal, qz)
1087	SFG	1	Sub	B	hem (cal, qz)
1087	SFG	1	Sub	B	hem (ab, lep, ms, qz)
1087	SFG	1	Ang	T	qz (hem)
1277	SFG	4	Aggl	DR	hem
1277	SFG	1	Aggl	DR	hem
1427	SFG	6	Aggl	R/DR	hem
1427	SFG	1	Aggl	R	hem (an)
1427	SFG	1	Sub	B	hem (an)
1427	SFG	2	Aggl	R	hem (fluo)

1481	SFG	3	Aggl	R	hem
1481	SFG	3	Ang/sub	B	hem
1481	SFG	1	Aggl	B	hem (fluo)
1481	SFG	1	Aggl	B	hem (an)
1491	SFG	1	Aggl	R	hem
1491	SFG	1	Sub	B	hem
1491	SFG	1	Ang	B	hem (an, fluo, mnt, C)
1491	SFG	2	Aggl	R/B	hem (C, ms)
1493	SFG	2	Aggl	R	hem
1493	SFG	1	Ang	DR	hem (qz)
1493	SFG	2	Ang/irr	B	hem (qz)
1526	SFG	1	Sub	R	hem (gth, ab)
1526	SFG	2	Aggl	B /Y	hem (gth, qz)
1526	SFG	1	Sub	DR	hem (gth, qz)
1526	SFG	1	Aggl	T	brt
1526	SFG	1	Aggl	T	brt (hem, gth)
1552	SFG	3	Aggl	DR	hem
1552	SFG	2	Aggl	DG	hem
1552	SFG	1	Aggl	DR	hem (und. Mn ox)
1566	SFG	2	Aggl	R/B	hem
1566	SFG	2	Ang	T/B	qz (hem)
1626	SFG	2	Aggl	DR	hem
1626	SFG	4	Aggl	R/DR	hem (gth)
1626	SFG	1	Aggl	R	hem (an, gth)
1626	SFG	1	Ang	B	hem (an, gth)
1626	SFG	1	Aggl	R	hem (brt)
1626	SFG	1	Aggl	R	hem (phl)
1635	SFG	1	Aggl	B	hem
1635	SFG	1	Aggl	DR	hem (an)
1635	SFG	1	Aggl	R	hem (ab, qz)
1635	SFG	1	Sub	W	qz (an, hem, gp)
1635	SFG	1	Ang	DG	qz (hem)
1637	SFG	1	Aggl	DR	hem
1637	SFG	2	Aggl	DR	hem (ab)
1637	SFG	1	Sub	DR	hem (ab)
1637	SFG	1	Aggl	DR	hem (ab, gth)
1677	SFG	4	Aggl	R/DR	hem
1677	SFG	1	Aggl	DG	hem
1677	SFG	1	Aggl	DR	hem (kln)
1677	SFG	1	Aggl	DR	hem (ab, kln)
1699	SFG	1	Aggl	DR	hem
1699	SFG	2	Irr/sub	B	hem
1699	SFG	3	Aggl	DR/B	hem (an)
1699	SFG	1	Aggl	R	hem (an, cal)
1699	SFG	1	Ang	B	qz (ab, hem)
1737	SFG	3	Aggl	R	hem
1737	SFG	1	Ang	B	hem
1737	SFG	3	Aggl	R	hem (gth)
1737	SFG	1	Aggl	DR	hem (ms)
1780	SFG	1	Aggl	DR	hem (gth)
1780	SFG	3	Aggl	R/DR	hem
1780	SFG	2	Ang	B	hem
1845	SFG	4	Aggl	R	hem
1845	SFG	1	Aggl	R	hem (mag)
1845	SFG	1	Aggl	R	hem (und. Mn ox)
1845	SFG	1	Aggl	R	hem (hp, qz)
1845	SFG	1	Aggl	R	hem (qz)
1845	SFG	1	Sub	B	hem (qz, ms)
1862	SFG	1	Aggl	DR	hem (fluo)
1862	SFG	1	Sub	B	hem (an)
1862	SFG	1	Aggl	DR	hem (cal, gp)
1914	SFG	6	Aggl	R/DR	hem
1914	SFG	1	Aggl	O	hem
1914	SFG	1	Sub	B	hem
1914	SFG	1	Aggl	DR	hem (an)
1914	SFG	1	Aggl	B	hem (mc)
1927	SFG	2	Aggl	R/DR	hem
1927	SFG	1	Sub	B	hem
1927	SFG	1	Aggl	R	hem (an, mag)
1942	SFG	4	Aggl	R/DR	hem
1942	SFG	1	Sub	B	hem

1981	SFG	4	Aggl	R/DR	hem
1981	SFG	3	Ang/irr	B	hem
1981	SFG	2	Aggl/sub	R	hem (gth)
1981	SFG	1	Ang	B	nt (hem, gth, ms)
1981	SFG	1	Ang	B	qz (hem, gth)
1981	SFG	1	Irr	T	qz (hem)
2023	SFG	2	Aggl	R/DR	hem
2023	SFG	1	Aggl	Y	gth
2023	SFG	1	Aggl	DG	gth (an)
2023	SFG	1	Aggl	DG	gth (ms)
2063	SFG	1	Aggl	R	hem
2063	SFG	1	Aggl	R	hem (gth, gp)
2063	SFG	3	Aggl	B	gth (hem, gp)
2063	SFG	1	Ang	B	C (hem)
2067	SFG	4	Aggl	R /DG	hem
2069	SFG	3	Aggl	R	hem
2069	SFG	1	Sub	B	hem
2091	SFG	2	Aggl	R	hem
2091	SFG	1	Sub	B	hem
2091	SFG	1	Aggl	DR	hem (gth)
2091	SFG	1	Aggl	B	gth (qz)
2091	SFG	1	Ang	W	rt (hem, gth)
2091	SFG	1	Ang	B	C
2159	SFG	2	Aggl	R/DG	hem
2159	SFG	1	Aggl	DG	hem (brt)
2159	SFG	1	Aggl	Y	gth (hem)
2159	SFG	1	Ang	B	qz (hem)
2159	SFG	1	Ang	W	rt (hem)
306	BFG	2	Aggl	R	hem (gth)
306	BFG	1	Sub	DR	hem (gth)
306	BFG	1	Aggl	Y	gth (hem)
306	BFG	3	Aggl	Y	gth
306	BFG	1	Sub	B	C
521	BFG	2	Aggl	R	hem (ms)
521	BFG	1	Ang	B	gth
521	BFG	2	Aggl	Y	gth
521	BFG	2	Aggl	Y	gth (hem)
901	BFG	1	Aggl	R	hem
901	BFG	2	Aggl/sub	B	gth
901	BFG	1	Aggl	DG	gth
901	BFG	1	Aggl	Y	gth
1499	BFG	1	Aggl	R	hem
1499	BFG	1	Aggl	DR	hem (gth)
1499	BFG	1	Ang	B	gth
1499	BFG	1	Aggl	Y	gth
1499	BFG	1	Subcirc	B	qz (hem, gth)
1499	BFG	1	Ang	T	qz (hem, mag)
1499	BFG	1	Aggl	B	qz (hem, gth, mag)
1499	BFG	1	Irr	B	lep
1700	BFG	3	Aggl	DR	hem
1700	BFG	1	Aggl	R	hem (ab)
1700	BFG	5	Aggl	Y	gth
1700	BFG	1	Aggl	B	gth
1700	BFG	1	Ang	B	gth (kln)
1806	BFG	2	Aggl	R	hem
1806	BFG	1	Sub	B	hem (qz)
1806	BFG	3	Aggl	Y	gth
1806	BFG	1	Aggl	B	gth
1806	BFG	1	Irr	B	gth (mag)
1806	BFG	1	Aggl	Y	gth (mag, hem)
2104	BFG	1	Aggl	R	hem
2104	BFG	2	Sub	B	hem
2104	BFG	5	Aggl	R/DR	hem (gth)
2104	BFG	1	Aggl	B	hem (gth, qz)
2104	BFG	4	Aggl	Y/O	gth (hem)
2104	BFG	1	Aggl	Y	gth (hem, qz)
102	HFG	1	Aggl	B	hem (fluo)
102	HFG	2	Aggl	B	gth
102	HFG	1	Aggl	Y	gth
102	HFG	1	Ang	T	qz (fluo, hem)
1419	HFG	2	Aggl	R	hem
1419	HFG	1	Sub	DR	hem
1419	HFG	1	Aggl	B	hem
1419	HFG	2	Aggl	B	hem (und. Mn ox)

1485	HFG	2	Aggl	R	hem
1485	HFG	1	Aggl	R	hem (gp)
1485	HFG	1	Aggl	B	hem (ab)
1485	HFG	1	Aggl	DG	hem (qz)
1585	HFG	1	Aggl	R	hem
1585	HFG	2	Aggl	DG	hem
1585	HFG	1	Aggl	R	hem (gth)
1734	HFG	5	Aggl	R/DR	hem
1752	HFG	2	Aggl	DR	hem
1752	HFG	2	Aggl	B	hem
2017	HFG	4	Aggl	DG	hem
2017	HFG	1	Aggl	R	hem (gp)
2047	HFG	3	Aggl	R/DG	hem
2047	HFG	1	Aggl	DG	hem
2282	HFG	2	Aggl	DG	hem
2282	HFG	1	Aggl	DG	hem (an)
2282	HFG	1	Aggl	R	hem (gp)
2282	HFG	1	Aggl	DG	hem (gth)
2375	HFG	4	Aggl	DG	hem
32	CG	1	Aggl	R	hem
32	CG	2	Aggl	B	hem
32	CG	1	Sub	B	qz (an, gth)
32	CG	1	Aggl	Y	mc (hem)
809	CG	1	Aggl	DG	hem
809	CG	1	Sub	DG	hem (qz)
809	CG	1	Aggl	R	hem (qz, ms)
809	CG	2	Ang	DG	qz (hem)
1510	CG	3	Aggl	B	hem
1510	CG	1	Sub	DR	hem
1510	CG	1	Aggl	B	hem (gth, ab)
1510	CG	1	Aggl	Y	gth
1510	CG	1	Aggl	Y	gth (cal)
1510	CG	1	Ang	B	qz (hem, mc)
1510	CG	1	Ang	W	qz (ms)
1510	CG	1	Aggl	W	cal (hem)
1580	CG	1	Aggl	DG	hem
1580	CG	1	Aggl	DR	hem (und. Mn ox)
1580	CG	1	Aggl	R	hem (lep)
1580	CG	1	Sub	T	qz (hem)
1628	CG	4	Aggl	R/DR	hem
1628	CG	1	Aggl	O	hem
1628	CG	1	Aggl	B	hem
1628	CG	1	Sub	B	hem (gth, und. Mn ox)
1628	CG	1	Aggl	O	gth
1628	CG	1	Aggl	Y	gth (gp, man)
1628	CG	2	Ang	T	qz (hem)
1666	CG	2	Aggl	R	hem
1666	CG	1	Aggl	Y	gth
1666	CG	1	Ang	T	qz
1666	CG	1	Aggl	B	qz (cal)
101	FS	1	Ang	R	hem
101	FS	1	Sub	B	hem
101	FS	5	Aggl/sub	R/DR	hem (qz)
101	FS	1	Aggl	DR	hem (an, qz)
101	FS	1	Aggl	R	hem (an, gth, hem, qz, ms)
101	FS	3	Aggl/irr	B	gth, hem
101	FS	1	Aggl	T	qz
385	FS	4	Aggl	R	hem (gth)
385	FS	1	Aggl	R	hem
385	FS	2	Aggl	Y	gth
385	FS	1	Aggl	BL	gth (qz)
385	FS	1	Ang	B	mag
385	FS	1	Ang	B	mag (hem)
965	FS	3	Aggl/ang	B	hem (qz)
965	FS	1	Ang	T	qz
965	FS	1	Ang	T	qz (hem)
973	FS	1	Sub	R	hem
973	FS	1	Sub	Y	gth
973	FS	2	Irr/sub	W	qz (hem)
1577	FS	2	Aggl	DR/B	hem
1577	FS	1	Aggl	DG	qz (hem, gth)
1577	FS	1	Ang	T	qz (hem, gth)

1679	FS	2	Aggl	R	hem
1679	FS	1	Sub	B	hem
1679	FS	1	Irr	DR	hem (ab)
1679	FS	1	Ang	T	qz
1679	FS	1	Irr	B	qz (ms)
1679	FS	1	Aggl	W	qz (hem, an, fluo)
436	PFG	2	Plat	B	hem
436	PFG	1	Aggl	B	hem
436	PFG	1	Plat	B	hem (gp, kln, qz)
1281	PFG	2	Aggl	R/DR	hem
1281	PFG	1	Aggl	B	hem
1281	PFG	1	Aggl	B	hem (mag, fluo)
1281	PFG	1	Plat	B	hem (mag, gp)
1603	PFG	2	Aggl	R/B	hem
1603	PFG	1	Aggl	DG	hem
1603	PFG	2	Plat	DG	hem
1812	PFG	2	Aggl	R/B	hem
1812	PFG	1	Ang	DR	hem (gth)
1812	PFG	1	Aggl	O	hem (gth)
1812	PFG	1	Aggl	B	hem (gth)
1812	PFG	1	Aggl	B	hem (an, gth, ms)
1985	PFG	2	Plat	DG	hem
1985	PFG	1	Aggl	DG	hem
1985	PFG	1	Plat	DG	hem (gth)

Num: number; mat: material; an: analyses; morph: morphology; SFG: soft fine-grained; BFG: banded fine-grained; HFG: hard fine-grained; CG: coarse-grained; FS: ferruginous sandstone; PFG: platy fine-grained; aggl: agglomerate; irr: irregular; sub: subcircular; ang: angular; plat: platy; tab: tabular; B: black; R: red; Y: yellow; T: translucent; DR: dark red; W: white; G: grey; DG: dark grey; O: orange. Abbreviations of minerals are based on the nomenclature suggested by [116], except for hp (hydroxylapatite); fluorapatite (fluo), lepidocrocite (lep), manganite (man) and natrojarosite (nt). Und: undetermined; ox: oxide; C: carbon.
* Minerals between brackets come from grains surrounding the analysed grain.

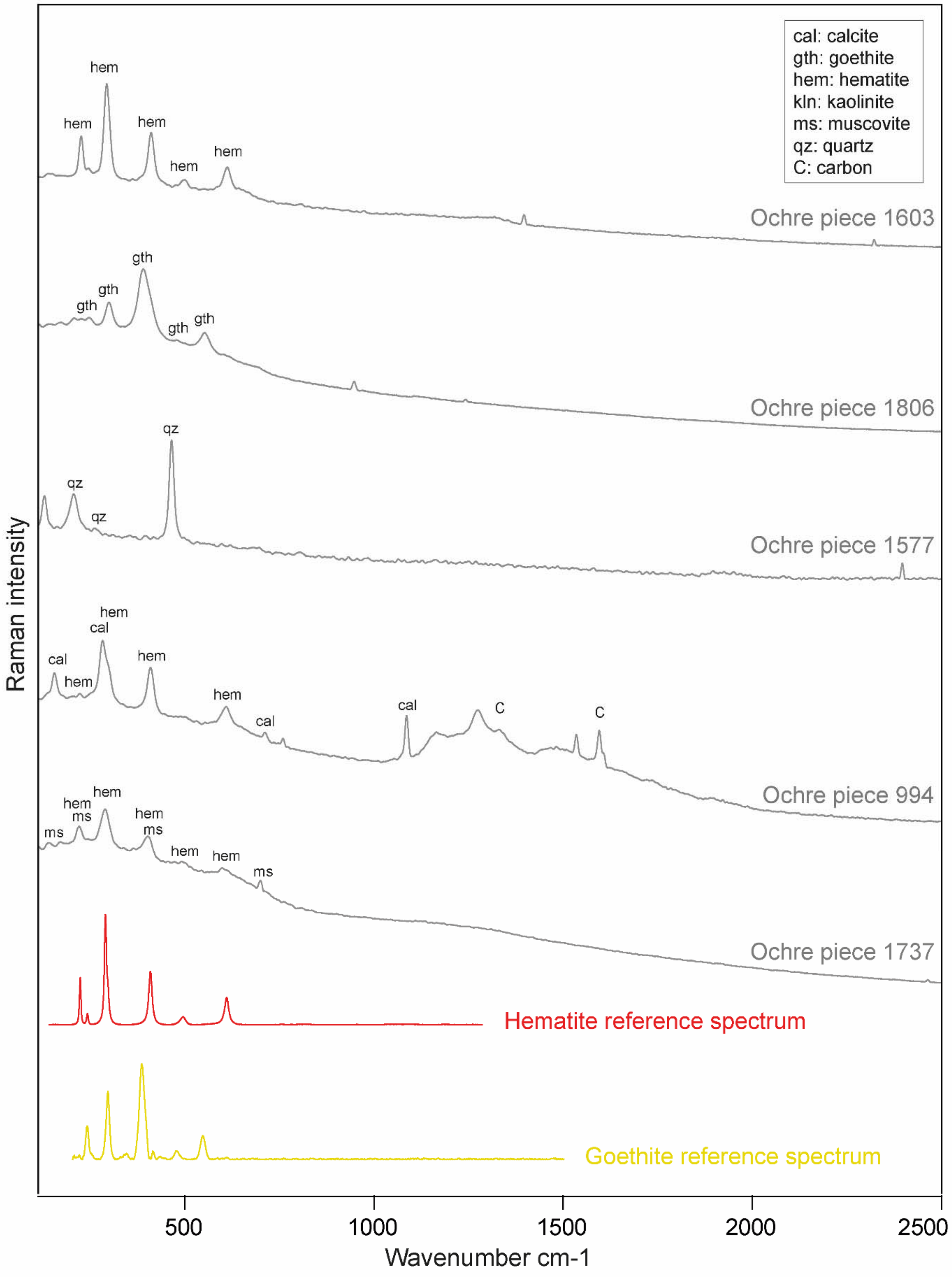

S6 Supplementary Figure 2. Representative µ-Raman spectra obtained from the analysis of Porc-Epic Cave ochre pieces.

The hematite and goethite reference spectra are indicated in red and yellow respectively: RRUFF ID R110013 and X050091[121].

S7 – Methods

S7 Supplementary Table 1. Number of analysed ochre pieces, ochre residues from ochre processing tools and ochre-stained artefacts from Porc-Epic Cave and natural ochre found in the wadi Laga Dächatu.

Artefact category	µ-RS	XRD	µXRD	SEM-EDS	EDXRF
Ochre pieces	80	12*	11	22	80
Ochre residue from OPT / OSA	20	11	-	12	-
Natural ochre pieces	-	-	6	-	39

µ-RS: micro-Raman spectroscopy; XRD: X-ray diffraction; µXRD: micro X-ray diffraction; SEM-EDS: Scanning electron microscopy coupled with energy dispersive X-ray spectroscopy; EDXRF: energy dispersive X-ray fluorescence; OPT: ochre processing tool; OSA: ochre-stained artefact.

* Analyses conducted on ochre powder samples obtained from grinding loose ochre microfragments.

S7 Supplementary Table 2. Contextual, macroscopic and technological data of analysed pieces and detailed account of analyses conducted on ochre pieces, ochre residues from ochre processing tools and ochre-stained artefacts from Porc-Epic Cave and natural ochre found in the wadi Laga Dächatu.

Artefact category*	Piece num**	Square	Depth	Accumulation area	Modifications	Colour***	Raw mat	μ-RS	XRD****	μ-XRD	XRF	SEM-EDS
OP	9	03N-08W	150-160	-	-	R	SFG	5			5	
OP	17	03N-11W	90-100	-	FK	R	SFG	9			5	10
OP	32	03N-12W	100-110	-	-	R	CG	3			5	
OP	37	03N-12W	100-110	-	-	R	SFG			1		
OP	51	04N-04W	70-80	SEA	-	R	SFG	3			5	
OP	101	04N-04W	80-90	SEA	-	O	FS	7			5	12
OP	102	04N-04W	80-90	SEA	G+SM	R + O	HFG	5			5	15
OP	133	04N-04W	80-90	SEA	FK	R	SFG	4			5	
OP	177	04N-05W	60-70	SEA	-	R + Y + O	BFG			1		
OP	282	04N-05W	70-80	SEA	G	R	SFG			1		
OP	295	04N-05W	80-90	SEA	-	R	SFG	3			5	10
OP	304	04N-05W	80-90	SEA	FK+G	R	SFG			1		
OP	306	04N-05W	80-90	SEA	FK+G+SC	Y	BFG	8			5	6
OP	334	04N-05W	100-110	-	P+G	R + G	SFG			1		
OP	363	04N-05W	110-120	-	FK	R	SFG			1		
OP	366	04N-05W	110-120	-	-	R + BR	FS			1		
OP	381	04N-05W	110-120	-	-	R + G	SFG	4			5	
OP	385	04N-05W	110-120	-	-	Y	FS	10			5	
OP	386	04N-05W	120-130	-	-	R + G	SFG			1		
OP	392	04N-05W	120-130	-	-	R	SFG			1		
OP	436	04N-07W	60-70	SEA	-	R + G	PFG	4	1		5	10
OP	512	04N-07W	60-70	SEA	-	R + G	CG			1		
OP	521	04N-07W	60-70	SEA	FK	R + Y	BFG	4			4	16
OP	538	04N-07W	60-70	SEA	-	R + G	SFG	5			5	12
OP	610	04N-07W	90-100	SEA	-	R + BL	SFG	5			5	
OP	615	04N-07W	90-100	SEA	-	R + BL	SFG	7	1		5	
OP	710	04N-07W	150-160	-	-	Y	SFG	6	1		5	
OP	725	04N-08W	60-70	-	FK	R	SFG	4			5	
OP	726	04N-08W	60-70	-	-	R	SFG	3			5	
OP	737	04N-08W	60-70	-	-	R	SFG	5			5	
OP	809	07N-15W	120-130	-	-	R + G	CG	5			5	
OP	857	08N-08W	100-110	NEA	FK+G+SC	R + G	HFG			1		
OP	901	08N-08W	110-120	NEA	-	Y	BFG	5			5	
OP	913	08N-08W	110-120	NEA	FK+G+SC	R	SFG	5			5	12
OP	919	08N-08W	110-120	NEA	-	R + G	SFG	6			5	11
OP	930	08N-08W	110-120	NEA	FK	Y	SFG	12			5	9
OP	934	08N-08W	120-130	NEA	G+SC	R + Y	BFG		1			
OP	965	04N-04W	70-80	SEA	-	R + G	FS	5			5	
OP	973	04N-07W	60-70	SEA	G	R	FS	4			5	12
OP	987	07N-13W	210-220	-	G+SC	R	SFG	3			4	10
OP	994	03N-04W	60-70	-	-	R + G	SFG	3	1		4	16
OP	1087	05N-07W	100-110	-	-	P	SFG	5			5	
OP	1277	06N-07W	180-190	-	G	R	SFG	5	1		5	
OP	1281	06N-07W	180-190	-	G	G	PFG	5			5	
OP	1419	05N-04W	70-80	-	FK+G+SC	G	HFG	6			5	11
OP	1427	05N-04W	100-110	-	SC	R + O	SFG	10			5	
OP	1481	05N-05W	100-110	-	-	R	SFG	8			5	
OP	1485	05N-05W	100-110	-	FK	R + G	HFG	5			5	
OP	1491	05N-05W	110-120	-	G	R	SFG	5			5	
OP	1493	05N-05W	110-120	-	FK+G	R	SFG	5			5	
OP	1499	05N-05W	130-140	-	FK+G	R + Y	BFG	8			5	
OP	1510	05N-05W	140-150	-	-	R + Y	CG	10	1		5	14
OP	1526	05N-07W	110-120	-	-	G	SFG	6			5	
OP	1552	05N-06W	160-170	-	FK	R	SFG	6			5	
OP	1566	05N-08W	100-110	-	-	R + P	SFG	4			5	
OP	1577	05N-08W	110-120	-	-	BR	FS	4			5	
OP	1580	05N-08W	110-120	-	FK	R + G	CG	4			5	
OP	1585	05N-08W	110-120	-	FK	G	HFG	4			5	
OP	1603	05N-08W	110-120	-	-	R + G	PFG	5			5	
OP	1626	05N-08W	120-130	-	G	R	SFG	10			5	
OP	1628	05N-08W	120-130	-	-	R	CG	11	1		5	11
OP	1635	05N-08W	140-150	-	G+SM	P	SFG	5	1		5	15
OP	1637	05N-08W	140-150	-	G	R	SFG	5			5	

OP	1666	05N-09W	120-130	-	FK	G	CG	5			5	
OP	1677	05N-09W	140-150	-	G	R	SFG	7			5	
OP	1679	05N-09W	140-150	-	-	R	FS	7	1		5	
OP	1699	05N-11W	130-140	-	G+SC	P	SFG	8			5	
OP	1700	05N-11W	130-140	-	G	R + O	BFG	11			5	
OP	1734	05N-14W	170-180	-	FK+G	R + G	HFG	5			5	
OP	1737	05N-14W	170-180	-	G	R	SFG	8			5	
OP	1752	05N-14W	190-200	-	G	P + G	HFG	4			5	12
OP	1780	06N-08W	120-130	-	FK+G	R	SFG	6			5	
OP	1806	06N-08W	130-140	-	FK+G	R + Y	BFG	9			5	
OP	1812	06N-08W	130-140	-	G	R + G	PFG	6	1		5	13
OP	1845	06N-08W	190-200	-	FK	R	SFG	9			5	
OP	1862	06N-09W	170-180	-	G	R	SFG	3			5	
OP	1914	07N-08W	120-130	-	FK+G	R	SFG	10			5	13
OP	1927	07N-08W	140-150	-	G	R	SFG	4			5	11
OP	1942	07N-08W	180-190	-	-	R	SFG	5			5	
OP	1981	07N-09W	200-210	-	-	R	SFG	12	1		5	
OP	1985	07N-09W	?	-	-	R + G	PFG	4			5	
OP	2017	07N-11W	130-140	-	G	R	HFG	5			5	
OP	2023	07N-11W	140-150	-	FK+G	R + P	SFG	4			5	
OP	2047	07N-11W	160-170	-	FK+G	R	HFG	4			5	
OP	2063	07N-11W	170-180	-	FK+G	R + O	SFG	6			5	
OP	2067	07N-11W	170-180	-	FK	R	SFG	4			5	
OP	2069	07N-11W	170-180	-	FK+G	R	SFG	4			5	
OP	2091	07N-11W	170-180	-	FK+G	P	SFG	7			5	
OP	2104	07N-12W	140-150	-	FK+G	R + P	BFG	14			5	
OP	2159	07N-14W	140-150	-	FK+SM	P	SFG	6			5	
OP	2282	08N-13W	110-120	-	FK+G	G	HFG	5			5	
OP	2375	08N-13W	150-160	-	FK+G	P	HFG	4			5	
OR (OPT)	1	04N-12W	130-140	-	NA	R + (Y)	NA	5	1			14
OR (OPT)	2	07N-09W	170-180	-	NA	R + Y	NA	7				14
OR (OPT)	3	10N-07W	160-170	NEA	NA	R	NA	5	1			12
OR (OPT)	4	08N-07W	150-160	NEA	NA	R+(Y)	NA	6				8
OR (OSA)	5	06N-07W	110-120	-	NA	R + B + (Y)	NA	4	1			11
OR (OPT)	6	08N-13W	150-160	-	NA	R + (Y)	NA	3	1			9
OR (OPT)	7	09N-07W	110-120	NEA	NA	R + (Y)	NA	12	1			13
OR (OPT)	8	-	120-130	-	NA	R + (Y)	NA	5	1			
OR (OPT)	9	08N-08W	110-120	NEA	NA	R + (B +Y)	NA	8	1			15
OR (OPT)	10	10N-07W	120-130	NEA	NA	R + (Y)	NA	8	1			
OR (OPT)	11	-	?	-	NA	R	NA	9				
OR (OPT)	12	-	150-160	-	NA	R	NA	5	1			6
OR (OPT)	13	08N-07W	150-160	NEA	NA	BRR + Y	NA	4				9
OR (OPT)	14	09N-07W	120-130	NEA	NA	R + (Y)	NA	7				
OR (OSA)	15	09N-07W	120-130	NEA	NA	R + (Y)	NA	7				9
OR (OPT)	16	09N-07W	120-130	NEA	NA	R + (Y)	NA	6				
OR (OPT)	17	09N-07W	120-130	NEA	NA	R + (Y)	NA	9	1			
OR (OPT)	18	09N-07W	120-130	NEA	NA	R + (Y + O)	NA	7	1			
OR (OPT)	19	09N-07W	120-130	NEA	NA	R + (Y)	NA	7				
OR (OPT)	22	07N-16W	200-210	-	NA	R + (Y)	NA	2				18
Nat	Geo1	NA	NA	NA	NA	R+Y	Nat			1	2	
Nat	Geo2	NA	NA	NA	NA	R+Y	Nat			1	2	
Nat	Geo3	NA	NA	NA	NA	R+Y	Nat			1		
Nat	Geo4	NA	NA	NA	NA	R+Y	Nat			1	1	
Nat	Geo5	NA	NA	NA	NA	R	Nat			1	1	
Nat	Geo6	NA	NA	NA	NA	G	Nat			1	3	
Nat	XP1	NA	NA	NA	NA	R	Nat				6	
Nat	XP3	NA	NA	NA	NA	R	Nat				3	
Nat	XP4	NA	NA	NA	NA	R	Nat				2	
Nat	XP5	NA	NA	NA	NA	R	Nat				2	
Nat	XP6	NA	NA	NA	NA	R	Nat				2	
Nat	Laga1	NA	NA	NA	NA	R	Nat				3	
Nat	Laga2	NA	NA	NA	NA	R	Nat				3	
Nat	Laga3	NA	NA	NA	NA	R	Nat				3	
Nat	Laga4	NA	NA	NA	NA	R	Nat				3	
Nat	Laga5	NA	NA	NA	NA	R	Nat				3	
Nat	Laga6	NA	NA	NA	NA	R	Nat				3	

Nat	Laga7	NA	NA	NA	NA	R	Nat	3
Nat	Laga8	NA	NA	NA	NA	R	Nat	3
Nat	Laga9	NA	NA	NA	NA	R	Nat	3
Nat	Laga10	NA	NA	NA	NA	R	Nat	3
Nat	Laga11	NA	NA	NA	NA	R	Nat	3
Nat	Laga12	NA	NA	NA	NA	R	Nat	3
Nat	Laga13	NA	NA	NA	NA	R	Nat	3
Nat	Laga14	NA	NA	NA	NA	R	Nat	3
Nat	Laga15	NA	NA	NA	NA	R	Nat	3
Nat	Laga16	NA	NA	NA	NA	R	Nat	3
Nat	Laga17	NA	NA	NA	NA	R	Nat	3
Nat	Laga18	NA	NA	NA	NA	R	Nat	3
Nat	Laga19	NA	NA	NA	NA	R	Nat	3
Nat	Laga20	NA	NA	NA	NA	R	Nat	3
Nat	Laga21	NA	NA	NA	NA	R	Nat	3
Nat	Laga22	NA	NA	NA	NA	R	Nat	3
Nat	Laga23	NA	NA	NA	NA	R	Nat	3
Nat	Laga24	NA	NA	NA	NA	R	Nat	3
Nat	Laga25	NA	NA	NA	NA	R	Nat	3
Nat	Laga26	NA	NA	NA	NA	R	Nat	3
Nat	Laga27	NA	NA	NA	NA	R	Nat	3
Nat	Laga28	NA	NA	NA	NA	R	Nat	3
Nat	Laga29	NA	NA	NA	NA	R	Nat	3

Num: number; raw mat: raw material; μ-RS: micro-Raman spectroscopy; XRD: X-ray diffraction; μ-XRD: micro X-ray diffraction; XRF: X-ray fluorescence; SEM-EDS: scanning electron microscopy coupled with energy dispersive X-ray spectroscopy; OP: ochre piece; OR: ochre residue; OPT: ochre processing tool; OSA: ochre-stained artefact; nat: natural ochre pieces; SEA: southeastern area; NEA: northeastern area; FK: flaking; G: grinding; SM: smoothing; SC: scraping; P: pitting; NA: not applicable; R: red; O: orange; Y: yellow; BR: brown; G: grey; BL: black; P: purple; SFG: soft fine-grained; BFG: banded fine-grained; HFG: hard fine-grained; CG: coarse-grained; FS: ferruginous sandstone; PFG: platy fine-grained. Numbers indicate the number of analyses conducted on the ochre pieces and residues.

(*) Analyses on ochre processing tools (OPT) and ochre-stained artefacts (OSA) were conducted on ochre residues sampled from their surface.

(**) In the case of OPT and OSA, piece numbers correspond to the artefact number where the ochre residue was found, not the ochre sample number.

(***) In brackets, coloured residue visible under microscopy.

(****) Analyses conducted on ochre powder samples obtained from grinding loose ochre microfragments originally attached to the indicated ochre piece or on ochre residues sampled from ochre processing tools and ochre-stained artefacts.

Supplementary references

1. d'Errico, F. & Stringer, C. B. Evolution, revolution or saltation scenario for the emergence of modern cultures? *Philos. Trans. R. Soc. B Biol. Sci.* **366**, 1060–1069 (2011). 10.1098/rstb.2010.0340
2. Henshilwood, C. S. & Marean, C. W. The origin of modern human behavior: Critique of the models and their test implications. *Curr. Anthropol.* **44**, 627–651 (2003).
3. McBrearty, S. & Brooks, A. S. The revolution that wasn't: a new interpretation of the origin of modern human behavior. *J. Hum. Evol.* **39**, 453–563 (2000). 10.1006/jhev.2000.0435
4. d'Errico, F. Le rouge et le noir: implications of early pigment use in Africa, the Near East and Europe for the origin of cultural modernity. *South Afr. Archaeol. Soc. Goodwin Ser.* **10**, 168–174 (2008).
5. Wadley, L. Recognizing Complex Cognition through Innovative Technology in Stone Age and Palaeolithic Sites. *Camb. Archaeol. J.* **23**, 163–183 (2013). 10.1017/S0959774313000309
6. Zilhão, J. The emergency of language, art and symbolic thinking in *Homo Symbolicus: The dawn of language, imagination and spirituality* (eds. Henshilwood, C. S. & D'Errico, F.) 111–131 (John Benjamins Publishing Company, 2011).
7. Brooks, A. S. *et al.* Long-distance stone transport and pigment use in the earliest Middle Stone Age. *Science* **360**, 90–94 (2018). 10.1126/science.aao2646
8. Conard, N. J. An overview of the patterns of behavioral change in Africa and Eurasia during the Middle and Late Pleistocene in *From Tools to Symbols from Early Hominids to Humans.* (eds. d'Errico, F. & Backwell, L.) 294–332 (Wits University Press, 2005).
9. d'Errico, F. The invisible frontier. A multiple species model for the origin of behavioral modernity. *Evol. Anthropol. Issues News Rev.* **12**, 188–202 (2003). 10.1002/evan.10113
10. d'Errico, F., García Moreno, R. & Rifkin, R. F. Technological, elemental and colorimetric analysis of an engraved ochre fragment from the Middle Stone Age levels of Klasies River Cave 1, South Africa. *J. Archaeol. Sci.* **39**, 942–952 (2012). 10.1016/j.jas.2011.10.032
11. d'Errico, F. & Banks, W. E. Identifying Mechanisms behind Middle Paleolithic and Middle Stone Age Cultural Trajectories. *Curr. Anthropol.* **54**, S371–S387 (2013). 10.1086/673388
12. Dubreuil, B. & Henshilwood, C. S. Material culture and language in *New Perspectives on the Origins of Language* (eds. Lefebvre, C., Comrie, B. & Cohen, H.) 147–170 (Studies in Language Companion Series 144, John Benjamins Publishing Company, 2013). 10.1075/slcs.144.06dub
13. Watts, I. The pigments from Pinnacle Point Cave 13B, Western Cape, South Africa. *J. Hum. Evol.* **59**, 392–411 (2010). 10.1016/j.jhevol.2010.07.006

14. Watts, I. Red ochre, body painting, and language: interpreting the Blombos ochre in *The Cradle of Language* (eds. Botha, R. & Knight, C.) 62–92 (Oxford University Press, 2009).
15. Watts, I. Ochre in the Middle Stone Age of Southern Africa: Ritualised Display or Hide Preservative? *South Afr. Archaeol. Bull.* **57**, 1–14 (2002). 10.2307/3889102
16. Zilhão, J. The Emergence of Ornaments and Art: An Archaeological Perspective on the Origins of "Behavioral Modernity". *J. Archaeol. Res.* **15**, 1–54 (2007). 10.1007/s10814-006-9008-1
17. Zilhão, J. *et al.* Symbolic use of marine shells and mineral pigments by Iberian Neandertals. *Proc. Natl. Acad. Sci.* **107**, 1023–1028 (2010). 10.1073/pnas.0914088107
18. Havenga, D. *et al.* From Himba indigenous knowledge to engineered Fe2O3 UV-blocking green nanocosmetics. *Sci. Rep.* **12**, 2259 (2022). 10.1038/s41598-021-04663-0
19. Kozowyk, P. R. B., Langejans, G. & Poulis, J. A. Lap Shear and Impact Testing of Ochre and Beeswax in Experimental Middle Stone Age Compound Adhesives. *PLoS ONE* **11**, e0150436 (2016). 10.1371/journal.pone.0150436
20. Rifkin, R. Assessing the efficacy of red ochre as a prehistoric hide tanning ingredient. *J. Afr. Archaeol.* **9**, 131–58 (2011). 10.3213/2191-5784-10199
21. Rifkin, R. F. Processing ochre in the Middle Stone Age: Testing the inference of prehistoric behaviours from actualistically derived experimental data. *J. Anthropol. Archaeol.* **31**, 174–195 (2012). 10.1016/j.jaa.2011.11.004
22. Rifkin, R. F., d'Errico, F., Dayet-Bouillot, L. & Summers, B. Assessing the photoprotective effects of red ochre on human skin by in vitro laboratory experiments. *South Afr. J. Sci.* **111**, 1–8 (2015). 10.17159/sajs.2015/20140202
23. Rifkin, R. F. *et al.* Evaluating the Photoprotective Effects of Ochre on Human Skin by In Vivo SPF Assessment: Implications for Human Evolution, Adaptation and Dispersal. *PLOS ONE* **10**, e0136090 (2015). 10.1371/journal.pone.0136090
24. Rossano, M. J. Making friends, making tools, and making symbols. *Curr. Anthropol.* **51**, S89–S98 (2010). 10.1086/650481
25. Velo, J. Ochre as Medicine: A Suggestion for the Interpretation of the Archaeological Record. *Curr. Anthropol.* **25**, 674–674 (1984). 10.1086/203205
26. Wadley, L. Compound-Adhesive Manufacture as a Behavioral Proxy for Complex Cognition in the Middle Stone Age. *Curr. Anthropol.* **51**, S111–S119 (2010). 10.1086/649836
27. Wadley, L. Putting ochre to the test: replication studies of adhesives that may have been used for hafting tools in the Middle Stone Age. *J. Hum. Evol.* **49**, 587–601 (2005). 10.1016/j.jhevol.2005.06.007

28. Wadley, L., Hodgskiss, T. & Grant, M. Implications for complex cognition from the hafting of tools with compound adhesives in the Middle Stone Age, South Africa. *Proc. Natl. Acad. Sci.* **106**, 9590–9594 (2009). 10.1073/pnas.0900957106

29. Wadley, L., Williamson, B. & Lombard, M. Ochre in hafting in Middle Stone Age southern Africa: a practical role. *Antiquity* **78**, 661–675 (2004). 10.1017/S0003598X00113298

30. Wynn, T. & Coolidge, F. L. Beyond Symbolism and Language: An Introduction to Supplement 1, Working Memory. *Curr. Anthropol.* **51**, S5–S16 (2010). 10.1086/650526

31. Zipkin, A. M., Wagner, M., McGrath, K., Brooks, A. S. & Lucas, P. W. An Experimental Study of Hafting Adhesives and the Implications for Compound Tool Technology. *PLOS ONE* **9**, e112560 (2014). 10.1371/journal.pone.0112560

32. Rifkin, R. F. Ethnographic and experimental perspectives on the efficacy of red ochre as a mosquito repellent. *South Afr. Archaeol. Bull.* **70**, 64–75 (2015).

33. Rosso D.E. Aproximación etnoarqueológica al uso de colorantes para el tratamiento del cabello: el caso de los Hamar (Etiopía). *Pyrenae* **48**, 123–149 (2017). 10.1344/Pyrenae2017.vol48num2.4

34. Hodgskiss, T. & Wadley, L. How people used ochre at Rose Cottage Cave, South Africa: Sixty thousand years of evidence from the Middle Stone Age. *PLOS ONE* **12**, e0176317 (2017). 10.1371/journal.pone.0176317

35. Barham, L. S. Systematic Pigment Use in the Middle Pleistocene of South-Central Africa. *Curr. Anthropol.* **43**, 181–190 (2002). 10.1086/338292

36. Dayet, L., Texier, P.-J., Daniel, F. & Porraz, G. Ochre resources from the Middle Stone Age sequence of Diepkloof Rock Shelter, Western Cape, South Africa. *J. Archaeol. Sci.* **40**, 3492–3505 (2013). 10.1016/j.jas.2013.01.025

37. Henshilwood, C. S. *et al.* A 100,000-Year-Old Ochre-Processing Workshop at Blombos Cave, South Africa. *Science* **334**, 219–222 (2011). 10.1126/science.1211535

38. Hodgskiss, T. An investigation into the properties of the ochre from Sibudu, KwaZulu-Natal, South Africa. *South. Afr. Humanit.* **24**, 99–120 (2012).

39. Mackay, A. & Welz, A. Engraved ochre from a Middle Stone Age context at Klein Kliphuis in the Western Cape of South Africa. *J. Archaeol. Sci.* **35**, 1521–1532 (2008). 10.1016/j.jas.2007.10.015

40. Marean, C. W. *et al.* Early human use of marine resources and pigment in South Africa during the Middle Pleistocene. *Nature* **449**, 905–908 (2007). 10.1038/nature06204

41. Shipton, C. *et al.* 78,000-year-old record of Middle and Later Stone Age innovation in an East African tropical forest. *Nat. Commun.* **9**, 1832 (2018). 10.1038/s41467-018-04057-3

42. Rosso, D. E. The first uses of colour: what do we know? *J. Anthropol. Sci.* **100**, 45–69 (2022). 10.4436/JASS.10005

43. Dapschauskas, R., Göden, M. B., Sommer, C. & Kandel, A. W. The Emergence of Habitual Ochre Use in Africa and its Significance for The Development of Ritual Behavior During The Middle Stone Age. *J. World Prehistory* **35**, 233–319 (2022). 10.1007/s10963-022-09170-2
44. Bodu, P. *et al.* An open-air site from the recent Middle Palaeolithic in the Paris Basin (France): Les Bossats at Ormesson (Seine-et-Marne). *Quat. Int.* **331**, 39–59 (2014). doi:10.1016/j.quaint.2013.10.029
45. Dayet, L. *et al.* Manganese and iron oxide use at Combe-Grenal (Dordogne, France): A proxy for cultural change in Neanderthal communities. *J. Archaeol. Sci. Rep.* **25**, 239–256 (2019). 10.1016/j.jasrep.2019.03.027
46. Peresani, M., Vanhaeren, M., Quaggiotto, E., Queffelec, A. & d'Errico, F. An Ochered Fossil Marine Shell from the Mousterian of Fumane Cave, Italy. *PLOS ONE* **8**, e68572 (2013). 10.1371/journal.pone.0068572
47. Peyrony, D. Le Moustier : ses gisements, ses industries, ses couches géologiques. *Rev. Anthropol.* **40**, 48–76, 155–176 (1930).
48. Soressi, M. *et al.* Pech-de-l'Azé I (Dordogne, France) : nouveau regard sur un gisement moustérien de tradition acheuléenne connu depuis le XIXème siècle. *Mém. Société Préhistorique Fr.* **47**, 95–132 (2008).
49. Bar-Yosef Mayer, D. E., Vandermeersch, B. & Bar-Yosef, O. Shells and ochre in Middle Paleolithic Qafzeh Cave, Israel: indications for modern behavior. *J. Hum. Evol.* **56**, 307–314 (2009). 10.1016/j.jhevol.2008.10.005
50. d'Errico, F., Salomon, H., Vignaud, C. & Stringer, C. Pigments from the Middle Palaeolithic levels of Es-Skhul (Mount Carmel, Israel). *J. Archaeol. Sci.* **37**, 3099–3110 (2010). 10.1016/j.jas.2010.07.011
51. Hovers, E., Ilani, S., Bar-Yosef, O. & Vandermeersch, B. An Early Case of Color Symbolism: Ochre Use by Modern Humans in Qafzeh Cave. *Curr. Anthropol.* **44**, 491–522 (2003). 10.1086/375869
52. Salomon, H. *et al.* Selection and heating of colouring materials in the Mousterian level of Es-Skhul (c. 100 000 years BP, Mount Carmel, Israel). *Archaeometry* **54**, 698–722 (2012). 10.1111/j.1475-4754.2011.00649.x
53. Paddayya, K. Excavation of an Acheulian Site at Hunsgi, South India. *Curr. Anthropol.* **17**, 760–761 (1976).
54. Petraglia, M. *et al.* Middle Paleolithic Assemblages from the Indian Subcontinent Before and After the Toba Super-Eruption. *Science* **317**, 114–116 (2007). 10.1126/science.1141564
55. Wang, F.-G. *et al.* Innovative ochre processing and tool use in China 40,000 years ago. *Nature* **603,** 284–289 (2022). 10.1038/s41586-022-04445-2.

56. Dayet, L., Le Bourdonnec, F.-X., Daniel, F., Porraz, G. & Texier, P.-J. Ochre Provenance and Procurement Strategies During The Middle Stone Age at Diepkloof Rock Shelter, South Africa. *Archaeometry* **58**, 807–829 (2016). 10.1111/arcm.12202
57. Hodgskiss, T. Ochre Use in the Middle Stone Age at Sibudu, South Africa: Grinding, Rubbing, Scoring and Engraving. *J. Afr. Archaeol.* **11**, 75–95 (2013). 10.3213/2191-5784-10232
58. Moyo, S. *et al.* Blombos Cave: Middle Stone Age ochre differentiation through FTIR, ICP OES, ED XRF and XRD. *Quat. Int.* **404**, 20–29 (2016). 0.1016/j.quaint.2015.09.041
59. Popelka-Filcoff, R. S. & Zipkin, A. M. The archaeometry of ochre sensu lato: A review. *J. Archaeol. Sci.* **137**, 105530 (2022). 10.1016/j.jas.2021.105530
60. d'Errico, F. *et al.* Trajectories of cultural innovation from the Middle to Later Stone Age in Eastern Africa: Personal ornaments, bone artifacts, and ocher from Panga ya Saidi, Kenya. *J. Hum. Evol.* **141**, 102737 (2020). 10.1016/j.jhevol.2019.102737
61. Rosso, D. E., d'Errico, F. & Queffelec, A. Patterns of change and continuity in ochre use during the late Middle Stone Age of the Horn of Africa: the Porc-Epic Cave record. *PLOS ONE* **12**, e0177298 (2017). 10.1371/journal.pone.0177298
62. Clark, J. D. & Kurashina, H. An Analysis of Earlier Stone Age Bifaces from Gadeb (Locality 8E), Northern Bale Highlands, Ethiopia. *South Afr. Archaeol. Bull.* **34**, 93–109 (1979). 10.2307/3887867
63. Leakey, L. S. B. Recent discoveries at Olduvai Gorge, Tanganyika. *Nature* **181**, 1099–1103 (1958). 10.1038/1811099a0
64. Mirazón Lahr, M. & Foley, R. A. Human Evolution in Late Quaternary Eastern Africa in *Africa from MIS 6-2 - Population Dynamics and Paleoenvironments* (eds. Jones, S. C. & Stewart, B. A.) 215–231 (Springer, 2016). 10.1007/978-94-017-7520-5_12
65. Tryon, C. A. & Faith, J. T. Variability in the Middle Stone Age of Eastern Africa. *Curr. Anthropol.* **54**, S234–S254 (2013). 10.1086/673752
66. Chavaillon, J. & Berthelet, A. The archaeological sites of Melka Kunture in *Studies on the Early Paleolithic site of Melka Kunture, Ethiopia.* (eds. Chavaillon, J. & Piperno, M.) 25–80 (Origines, 2004).
67. Deino, A. L. & McBrearty, S. 40Ar/39Ar dating of the Kapthurin Formation, Baringo, Kenya. *J. Hum. Evol.* **42**, 185–210 (2002). 10.1006/jhev.2001.0517
68. McBrearty, S. The Middle Pleistocene of East Africa in *Human roots: Africa and Asia in the Middle Pleistocene.* (eds. Barham, L. & Robson Brown, K.) 81–92 (Western Academic and Specialist Press, 2001).
69. Van Peer, P., Rots, V. & Vroomans, J.-M. A story of colourful diggers and grinders. *Farming* **2004**, 1–28 (2004). 10.3828/bfarm.2004.3.1

70. Van Peer, P. *et al.* The Early to Middle Stone Age transition and the emergence of modern human behaviour at site 8-B-11, Sai Island, Sudan. *J. Hum. Evol.* **45**, 187–193 (2003). 10.1016/S0047-2484(03)00103-9
71. Conard, N. J. World Heritage and the Middle Stone Age: examples from East Africa and South Africa in *Human Origins Sites and the World Heritage Convention in Africa.* (ed. Sanz, N.) 18–30 (UNESCO, 2012).
72. Diez-Martín, F. *et al.* The Middle to Later Stone Age technological transition in East Africa. New data from Mumba Rockshelter Bed V (Tanzania) and their implications for the origin of modern human behavior. *J. Afr. Archaeol.* **7**, 147–173 (2009). 10.3213/1612-1651-10136
73. Gliganic, L. A., Jacobs, Z., Roberts, R. G., Domínguez-Rodrigo, M. & Mabulla, A. Z. P. New ages for Middle and Later Stone Age deposits at Mumba rockshelter, Tanzania: Optically stimulated luminescence dating of quartz and feldspar grains. *J. Hum. Evol.* **62**, 533–547 (2012). 10.1016/j.jhevol.2012.02.004
74. Mehlman, M. J. Late Quaternary archaeological sequences in northern Tanzania (Doctoral dissertation, University of Illinois, 1989).
75. Prendergast, M. E. *et al.* New Excavations at Mumba Rockshelter, Tanzania. *J. Afr. Archaeol.* **5**, 217–243 (2007). 10.3213/1612-1651-10093
76. Tryon, C. A. & Ranhorn, K. New Radiocarbon Dates from Nasera Rockshelter (Tanzania): Implications for Studying Spatial Patterns in Late Pleistocene Technology. *J. Afr. Archaeol.* **16**, (2018). 10.1163/21915784-20180011
77. Inskeep, R. R. The age of the Kondoa rock paintings in the light of recent excavations at Kisese II rock shelter in *Actes du IVe Congrès Panafricain de Préhistoire et l'Étude du Quaternaire* (eds. Mortelmans, G. & Nenquin, J.) 249–256 (Musée royal de l'Afrique centrale, 1962).
78. Leakey, M. *Africa's vanishing art: the rock paintings of Tanzania*. (Doubleday, 1983).
79. Tryon, C. A. *et al.* Middle and Later Stone Age chronology of Kisese II rockshelter (UNESCO World Heritage Kondoa Rock-Art Sites), Tanzania. *PLOS ONE* **13**, e0192029 (2018). 10.1371/journal.pone.0192029
80. Ambrose, S. H. Chronology of the Later Stone Age and Food Production in East Africa. *J. Archaeol. Sci.* **25**, 377–392 (1998). 10.1006/jasc.1997.0277
81. Brandt, S., Hildebrand, E., Vogelsang, R., Wolfhagen, J. & Wang, H. A new MIS 3 radiocarbon chronology for Mochena Borago Rockshelter, SW Ethiopia: Implications for the interpretation of Late Pleistocene chronostratigraphy and human behavior. *J. Archaeol. Sci. Rep.* **11**, 352–369 (2017). 10.1016/j.jasrep.2016.09.013
82. Brandt, S. A. *et al.* Early MIS 3 occupation of Mochena Borago Rockshelter, Southwest Ethiopian Highlands: Implications for Late Pleistocene archaeology, paleoenvironments and modern human dispersals. *Quat. Int.* **274**, 38–54 (2012). 10.1016/j.quaint.2012.03.047

83. Kelsey, B., Brandt, S., Zipkin, A. M. & Wilson, E. Provenance of ochre at Mochena Borago Rockshelter, SW Ethiopia: an interdisciplinary approach. East African Association for Paleoanthropology and Paleontology (EAAPP) seventh biennial conference, Nairobi. https://eaappinfo.wordpress.com/2019-2/ (2019).
84. Clark, J. D. The Middle Stone Age of East Africa and the beginnings of regional identity. *J. World Prehistory* **2**, 235–305 (1988). 10.1007/BF00975618
85. Leakey, L. S. B. The Industries of the Gorgora rock shelter, lake Tana. *J. East Afr. Nat. Hist. Soc.* **XVII**, 199–203 (1943).
86. Moysey, F. Excavation of a rockshelter at Gorgora, Lake Tana, Ethiopia. *J. East Afr. Uganda Nat. Hist. Soc.* **17**, 196–198 (1943).
87. Yellen, J. *et al.* The Archaeology of Aduma Middle Stone Age Sites in the Awash Valley, Ethiopia. *PaleoAnthropology* **10**, 25–100 (2005).
88. Leplongeon, A. Microliths in the Middle and Later Stone Age of eastern Africa: New data from Porc-Epic and Goda Buticha cave sites, Ethiopia. *Quat. Int.* **343**, 100–116 (2014). 10.1016/j.quaint.2013.12.002
89. Pleurdeau, D. *et al.* Cultural change or continuity in the late MSA/Early LSA of southeastern Ethiopia? The site of Goda Buticha, Dire Dawa area. *Quat. Int.* **343**, 117–135 (2014). 10.1016/j.quaint.2014.02.001
90. Tribolo, C. *et al.* Across the Gap: Geochronological and Sedimentological Analyses from the Late Pleistocene-Holocene Sequence of Goda Buticha, Southeastern Ethiopia. *PLOS ONE* **12**, e0169418 (2017). 10.1371/journal.pone.0169418
91. Rosso, D. E., Martí, A. P. & d'Errico, F. Middle Stone Age Ochre Processing and Behavioural Complexity in the Horn of Africa: Evidence from Porc-Epic Cave, Dire Dawa, Ethiopia. *PLOS ONE* **11**, e0164793 (2016). 10.1371/journal.pone.0164793
92. Rosso, D. E., d'Errico, F. & Zilhão, J. Stratigraphic and spatial distribution of ochre and ochre processing tools at Porc-Epic Cave, Dire Dawa, Ethiopia. *Quat. Int.* **343**, 85–99 (2014). 10.1016/j.quaint.2013.10.019
93. Teilhard de Chardin, P. Le Paléolithique en Somalie française et en Abyssinie. *Anthropol.* **40**, 331–334 (1930).
94. Teilhard de Chardin, P., Breuil, H. & Wernert, P. Les Industries lithiques de Somalie française. *Anthropol.* **49**, 497–522 (1940).
95. Clark, J. D. & Williamson, K. D. A Middle Stone Age occupation site at Porc Epic Cave, Dire Dawa (east-central Ethiopia), Part I. *Afr. Archaeol. Rev.* **2**, 37–64 (1984). 10.1007/BF01117225
96. Breuil, H. Peintures rupestres préhistoriques du Harrar (Abyssinie). *L'Anthropologie* **44**, 473–483 (1934). 10.3406/crai.1934.86588

97. Clark, J. D. & Williams, M. A. J. Recent Archaeological Research in Southeastern Ethiopia. 1974 - 1975. *Ann. Ethiop.* **11**, 19–44 (1978). 10.3406/ethio.1978.902

98. Pleurdeau, D. *Gestion des matières premières et comportements techniques dans le Middle Stone Age africain : les assemblages lithiques de la grotte du Porc-Épic, Dire Dawa, Éthiopie* (British Archaeological Reports International Series;1317: Archaeopress, 2004).

99. Assefa, Z. Faunal remains from Porc-Epic: Paleoecological and zooarchaeological investigations from a Middle Stone Age site in southeastern Ethiopia. *J. Hum. Evol.* **51**, 50–75 (2006). 10.1016/j.jhevol.2006.01.004

100. Michels, J. W. & Marean, C. A. A Middle Stone Age occupation site at Porc Epic Cave, Dire Dawa (east-central Ethiopia), Part II. *Afr. Archaeol. Rev.* **2**, 64–71 (1984). 10.1007/BF01117225

101. Anovitz, L. M., Elam, J. M., Riciputi, L. R. & Cole, D. R. The Failure of Obsidian Hydration Dating: Sources, Implications, and New Directions. *J. Archaeol. Sci.* **26**, 735–752 (1999). 10.1006/jasc.1998.0342

102. Ridings, R. Where in the World Does Obsidian Hydration Dating Work? *Am. Antiq.* **61**, 136–148 (1996). 10.2307/282308

103. Assefa, Z., Lam, Y. M. & Mienis, H. K. Symbolic Use of Terrestrial Gastropod Opercula during the Middle Stone Age at Porc-Epic Cave, Ethiopia. *Curr. Anthropol.* **49**, 746–756 (2008). 10.1086/589509

104. Bronk Ramsey, C. Radiocarbon calibration and analysis of stratigraphy; the OxCal program. *Radiocarbon* **37**, 425–430 (1995).

105. Breuil, H., Teilhard de Chardin, P. & Wernert, P. Le Paléolithique du Harrar. *L'Anthropologie* **55**, 219–230 (1951).

106. Leplongeon, A. La transition Middle Stone Age / Later Stone Age en Afrique de l'Est (Ethiopie) (Doctoral dissertation, Muséum National d'Histoire Naturelle, 2013).

107. Negash, A. & Shackley, M. S. Geochemical provenance of obsidian artefacts from the MSA site of Porc Epic, Ethiopia. *Archaeometry* **48**, 1–12 (2006). 10.1111/j.1475-4754.2006.00239.x

108. Perlès, C. Réexamen Typologique de L'Industrie du Porc-Épic Éthiopie : Les Pointes et Pièces Pointues. *Anthropol.* **78**, 529–551 (1974).

109. Pleurdeau, D. Human Technical Behavior in the African Middle Stone Age: The Lithic Assemblage of Porc-Epic Cave (Dire Dawa, Ethiopia). *Afr. Archaeol. Rev.* **22**, 177–197 (2005). 10.1007/s10437-006-9000-7

110. Pleurdeau, D. The lithic assemblage of the 1975-1976 excavation of the Porc-Epic Cave, Dire-Dawa, Ethiopia. Implications for the East African Middle Stone Age. *J. Afr. Archaeol.* **3**, 117–126 (2005). 10.3213/1612-1651-10040

111. Pleurdeau, D. Le Middle Stone Age de la grotte du Porc-Épic (Dire Dawa, Éthiopie) : gestion des matières premières et comportements techniques. *Anthropol.* **107**, 15–48 (2003). 10.1016/S0003-5521(02)00003-1

112. Vogel, N., Nomade, S., Negash, A. & Renne, P. R. Forensic 40Ar/39Ar dating: a provenance study of Middle Stone Age obsidian artifacts from Ethiopia. *J. Archaeol. Sci.* **33**, 1749–1765 (2006). 10.1016/j.jas.2006.03.008

113. Robinson, J. R. Thinking locally: Environmental reconstruction of Middle and Later Stone Age archaeological sites in Ethiopia, Kenya, and Zambia based on ungulate stable isotopes. *J. Hum. Evol.* **106**, 19–37 (2017). 10.1016/j.jhevol.2017.01.013

114. Robinson, J. R. Investigating habitat heterogeneity of Late Pleistocene archaeological sites in eastern Africa from stable isotopes. *Hist. Biol.* **34**, 674–693 (2022). 10.1080/08912963.2021.1942465

115. Vallois, H. V. La Mandibule Humaine Fossile de la Grotte du Porc-Épic près de Diré-Daoua Abyssinie. *Anthropol.* **55**, 231–238 (1951).

116. Whitney, D. L. & Evans, B. W. Abbreviations for names of rock-forming minerals. *Am. Mineral.* **95**, 185–187 (2010).

117. Hodgskiss, T. Cognitive Requirements for Ochre Use in the Middle Stone Age at Sibudu, South Africa. *Camb. Archaeol. J.* **24**, 405–428 (2014). 10.1017/S0959774314000663

118. Hodgskiss, T. Identifying grinding, scoring and rubbing use-wear on experimental ochre pieces. *J. Archaeol. Sci.* **37**, 3344–3358 (2010). 10.1016/j.jas.2010.08.003

119. Adams, J. *et al.* Functional analysis of macro-lithic artefacts in *Non-flint Raw Material Use in Prehistory: Old Prejudices and New Directions. Proceedings of the XV Congress of the UISPP; 2006 Sept 4–9; Lisbon, Portugal* (eds. Sternke, F., Eigeland, L. & Costa, L.-J.) 43–66 (British Archaeological Reports, International Series;1939: Archaeopress, 2009).

120. de Beaune, S. *Pour une archéologie du geste : Broyer, moudre, piler, des premiers chasseurs aux premiers agriculteurs* (CNRS Editions, 2000).

121. Downs, R. T. The RRUFF Project: an integrated study of the chemistry, crystallography, Raman and infrared spectroscopy of minerals in *Program and Abstracts of the 19th General Meeting of the International Mineralogical Association in Kobe, Japan* 3–13 (2006).
